# Supplementary material for: Reviewing the 95 sociotechnical barriers to the decarbonization of buildings
Source: Nat Commun. 2025 Nov 13;16:9983. doi: 10.1038/s41467-025-64923-9 (PMC12615690; doi:10.1038/s41467-025-64923-9)
Supplement: Supplementary file 1 — Supplementary Information [file 41467_2025_64923_MOESM1_ESM.pdf]

Supplementary Information

Reviewing the 95 sociotechnical barriers to the decarbonization of buildings

Erin Heinz[1*], Benjamin K. Sovacool[1], Thomas Kwan[2], and Vincent Petit[2]

[1] Institute for Global Sustainability, Boston University, Boston, United States

[2] Sustainability Research Institute, Schneider Electric [TM], Boston, United States

*Correspondence to Erin Heinz, eheinz@bu.edu

Supplementary Note:

This file contains additional materials supporting the main text of this study. Included are Supplementary Methodology Tables which provide the search review methodology and syntax, examples of qualitative codes, as well as a list of citations of relevant academic, political, and media source material used to inform the literature review.

Contents

Supplementary Table 1. Literature Review Search Methodology Utilized for Study 3

Supplementary Table 2. Inclusion and Exclusion Criteria for Literature Review 4

Supplementary Table 3. Examples of Qualitative Codes grouped by Themes 5

Supplementary References: Academic Literature 9

Supplementary References: Legislative Sources 54

Supplementary References: News Media 64

Supplementary Table 1. Literature Review Search Methodology Utilized for Study

<table>
<tr><th>Core Search Terms</th><th>Database Emphasis</th><th>Initial Return</th><th>Add. Filters</th><th>Records Export</th><th>Final Corpus</th></tr>
<tr><td rowspan="8">("barriers" OR "challenges" OR "obstacles" OR "hurdles" OR "constraints" OR “impediments”)) AND ("buildings" OR "construction" OR "green building" OR "low-carbon construction" OR "sustainable architecture" OR "energy-efficient buildings" OR "zero-carbon buildings" OR "net-zero construction" OR "decarbonized buildings" OR "eco-friendly construction")) AND AB=(("policy" OR "regulatory" OR "legal" OR "government regulations" OR "environmental policy" OR "climate policy" OR "building legislation" OR "zoning laws" OR "building codes" OR "institutional inertia" OR "bureaucratic hurdles" OR "regulatory uncertainty" OR "governmental resistance" OR "policy uncertainty" OR "administrative bottlenecks" OR "cultural resistance" OR "carbon lock-in" OR "public resistance" OR "social barriers" OR "community opposition" OR "behavioral resistance" OR "risk aversion" OR "knowledge gaps" OR "lack of awareness" OR "information barriers" OR "technical knowledge barriers" OR "skill shortages" OR "market challenges" OR "market barriers" OR "economic barriers" OR "market constraints" OR "lobbyist groups" OR "financial constraints" OR "cost barriers" OR "investment challenges" OR "funding gaps" OR "capital shortages" OR "economic hurdles" OR "upfront costs" OR "lack of incentives" OR "insufficient subsidies" OR "stakeholder alignment" OR "stakeholder collaboration" OR "stakeholder engagement" OR "misinformation" OR "industry lobby" OR "supply chain issues" OR "material shortages" OR "supply constraints" OR "supply chain disruptions" OR "policy uncertainty" OR "policy misalignment" OR "policy fragmentation" OR "regulatory enforcement" OR "financing challenges" OR "investment shortages" OR "credit barriers" OR "tenant attitudes" OR "consumer behavior" OR "end-user preferences" OR "public perception" OR "awareness barriers" OR "collaboration challenges" OR "multi-stakeholder barriers" OR "inter-agency barriers" OR "coordination issues" OR "behavioral barriers" OR "habitual obstacles" OR "resistance to change" OR "attitudinal barriers"))</td>
<td>Environmental Complete:
EBSCO full-text database for environmental studies, ecosystem ecology, energy, environmental policy, sustainability

Added Value: high relevance, some specialty journals not captured in WoS, recommended by libraries</td><td>571</td><td>31</td><td rowspan="3">Total: 3927

Screened for Relevance/ Duplicates: N=880</td><td rowspan="3">N=880
Global Academic Studies</td></tr>
<tr><td>Web of Science Core:
Multidisciplinary database of 92 million records, emphasis on journals

Added Value: Broad scope, used widely in systematic reviews, more comprehensive than SCOPUS in social science coverage</td><td>4989</td><td>1000</td></tr>
<tr><td>ProQuest Central:
Covers both academic journals and grey literature.

Added Value: Provides additional coverage from third “big database;” includes specialty Political Science Database</td><td>6687</td><td>1373</td></tr>
<tr><td></td><td></td><td></td><td></td><td></td></tr>
<tr><td rowspan="4">Nexus Uni: news, legal, and business content (last 5 years for current discussion and political testimonies) 2019-2024

Added Value: Large grey literature database, legal and industry literature</td><td>Document Type</td><td>Add. Filters</td><td>Records Export</td><td>Final Corpus</td></tr>
<tr><td>Statutes and Legislation (US)</td><td>60</td><td rowspan="2">174</td><td rowspan="2">N=175
US Statutes, Legislative Documents, and Political</td></tr>
<tr><td>Political Testimonies (US)</td><td>114</td></tr>
<tr><td>News
(Top 3000+ global sources)</td><td>192</td><td>192</td><td>N=192
Global News Media</td></tr>
</table>

Summary of literature search methodology, databases, and filtering process used to compile the final corpus for the study's literature review.

Supplementary Table 2. Inclusion and Exclusion Criteria for literature review

**Inclusion: Must be present to qualify for literature review**	
Relevance	The study specifically addresses barriers to decarbonization in commercial or residential building sector.
Type of Study	Includes empirical studies (quantitative and qualitative), case studies, which discuss social, behavioral, economic, and/or political barriers to decarbonization.
Publication Date	Academic studies published within the last 15 years (e.g., 2009 2024), and political and media resources in the last 5 years (e.g., September 2019-2024), to ensure relevance to current technologies and policies.
Geographical Focus	Global with comparative categories of United States, Europe, China, and Other
Language	Studies published in English or with available English translations.
Focus on Barriers	Studies focus on identifying, analyzing, or discussing barriers, impediments, challenges in the decarbonization process.
**Exclusion: Disqualifies the study from literature review**	
Irrelevance	Studies do not focus on barriers to decarbonization, or those that address general climate change impacts without specific attention to decarbonization challenges.
Outdated Sources	Academic studies published more than 15 years ago (e.g., before 2009), or 5 years (e.g., before 2019) for political and media resources.
Language	Studies in languages for which reliable English translations are not available.
Focus on Solutions	Studies focus primarily on solutions or strategies for overcoming barriers without discussing the barriers themselves.

Note: Inclusion and exclusion criteria used to screen literature for relevance to barriers in building sector decarbonization, including criteria topic focus, study type, publication date, geography, and language.

Supplementary Table 3. Examples of qualitative codes grouped by categorical themes with examples from the literature

Code	Examples from Literature	
Economic		
High Costs	One of the main challenges is the high initial cost of these materials. Smart and green building materials are usually made using advanced technology and eco-friendly manufacturing processes, which raises production costs, particularly in the startup stage. This cost barrier prevents many developers, especially those on limited budgets, from using these materials widely.[i]	The main obstacles and shortcomings faced are high upfront costs and delays, design complexities and documentation requirements, superior performance enhancement requirements, and skewness towards environmental sustainability.[ii]
Long ROI	The rather long payback time for investments render Energy Services Companies' (ESCOs) services financially unattractive for many ESCOs and building residents, thus hindering a large potential of energy savings in a sector that is responsible of almost half of Europe's energy consumption.[iii]	Many interviewees in this study indicated their preference to buying technologies (such as TES) only if the payback period is less than 5 years. Even with currently available financial incentives, the payback period of adding TES to existing HVAC systems may be longer than 5 years, especially if the electricity price is not based on the time of use.[iv]
Political		
Lack of Regulation	The need for regulations: Executive regulations in this regard and their coordination at international level to create a common language, reduce conflicts and create a clear path to resolving the dispute are essential.[v]	Besides, public perceptions toward a new change and a lack of regulatory authorities are marked as major obstacles for implementing green building practices. Enforcing fair rules and regulations may aid in overcoming these challenges.[vi]
Reporting Requirements	At the local level, the adoption of CE principles faces significant hurdles due to extensive bureaucratic processes involving administrative and organizational tasks. The challenges in coordinating different units and departments pose significant hurdles to effectively implementing CE principles within the GCI.[vii]	The most important legal barrier was the 'complex procedure to obtain approvals, complex codes, and regulations,' with mean score of 2.64 the impact of this barrier on development of sustainable buildings was assessed less effective.[viii]
Behavioral		
Lack of Stakeholder Alignment	Moreover, the construction industry in the country is still characterised by hostile relationships between main contractors and the supply chain. Hence, communication efforts between the subcontractors would be less effective than strengthening the influence of the main contractors in the industry.[ix]	A lack of integration among stakeholders is one of the key obstacles to GHG emissions mitigation actions in the building sector. The plurality of participants throughout all life cycle phases creates complicated relationships in the building sector. There are different actors intervening in building design, construction, financing, operation, and maintenance processes.[x]
Resistance to Change	Sustainability requires new ways of thinking, methods, practices, and attitude. Hence, it requires change. But as normally happens when implementing a new initiative; there is a resistance to change. This problem may exist at all levels; from client organisations, all the way through to the supply side and funding organisations… In the supply side, the problem may be related to a passive culture, where no change would be initiated unless deemed necessary by the client.[xi]	"Resistance to change from conventional to sustainable buildings<br>(from both people and companies)" was ranked as the second important social factor influencing the sustainable buildings development. This resistance to change could be due to the difficulty to change the construction methods and trends in the construction industry that have been used over the past decades.[60]

Supplementary Table 3. Cont.

Code	Examples from Literature	Code
Social		
Lack of Awareness	Insufficient investment in energy efficiency measures can also be attributed to the lack of information that is due to a lack of energy literacy and motivation to acquire it. Residents who are the final beneficiaries of apartment building modernization projects are often unaware of the benefits of introducing energy efficiency measures…[xii]	Many politicians, building owners, and construction professionals lack an understanding of the benefits of energy efficiency, as well as the technical knowledge and skills necessary to adopt energy-efficient techniques.[xiii]
Uncertain Risk of New Technologies	The fear for higher investment costs of green buildings compared to traditional buildings and the risks of unforeseen costs are among the most commonly addressed barriers for green building.[xiv]	Many aspects of sustainable design and construction are based upon proven technology and are being increasingly incorporated into best practices for the design and construction industries, which might lead to legal problems for those who do not include the more widely accepted sustainability measures in their projects.[xv]
Technical		
Lack of Data	Lack of data and knowledge is also perceived to be a large hindrance to the application of green building with "insufficient cost-benefit data" and "lack of technical understanding between the project stakeholders" having the same mean value of 3.85/5.[xvi]	there is a deficiency of research on the real implementation of 3D printing in the industry. This deficiency also affects top management in construction organization's willingness to adopt this technology for their projects.[xvii]

[i] Rajendra, P. & Mohanasundaram, T. Factors driving consumer adoption of smart and green building materials: The role of civil engineers and architects. *J. Asian Archit. Build. Eng.* (2024). doi:10.1080/13467581.2024.2373819.
[ii] Raouf, A. M. I. & Al-Ghamdi, S. G. Building information modelling and green buildings: challenges and opportunities. *Archit. Eng. Des. Manag.* 15, 1–28 (2019). doi:10.1080/17452007.2018.1502655.
[iii] Aranda, J., Tsitsanis, T., Georgopoulos, G. & Longares, J. M. Innovative data-driven energy services and business models in the domestic building sector. *Sustainability* 15, 3742 (2023). doi:10.3390/su15043742.
[iv] Anand, J., Liu, X., Wang, L. & Malhotra, M. An assessment of existing barriers to market adoption of thermal energy storage in buildings based on interviews with stakeholders. In *ASHRAE Transactions* 130, 52–61 (2024) ASHRAE Winter Conference, Chicago, United States, Jan 20–24, 2024.
[v] Majrouhi Sardroud, J. & Mehdizadehtavasani, M., Khorramabadi, A. & Ranjbardar, A. Barriers analysis to effective implementation of BIM in the construction industry. In *Proceedings of the 35th International Symposium on Automation and Robotics in Construction (ISARC 2018)* (2018).
[vi] Chowdhury, M. A., Sabrina, H., Zzaman, R. & Islam, S. L. U. Green building aspects in Bangladesh: A study based on experts' opinion regarding climate change. *Environ. Dev. Sustain.* 24, (2022). doi:10.1007/s10668-021-01823-0.
[vii] Abdulai, S. F. A., Nani, G., Taiwo, R., Antwi-Afari, P., Zayed, T. & Sojobi, A. O. Modelling the relationship between circular economy barriers and drivers for sustainable construction industry. *Build. Environ.* 254, 111388 (2024). https://doi.org/10.1016/j.buildenv.2024.111388.
[viii] Dalirazar, S. & Sabzi, Z. Strategic analysis of barriers and solutions to development of sustainable buildings using PESTLE technique. *Int. J. Constr. Manag.* 23, 1–30 (2020). doi:10.1080/15623599.2020.1854931.
[ix] Rasheed, A., Booth, C. & Horry, R. Stakeholder perceptions of the benefits and barriers of implementing environmental management systems in the Maldivian construction industry. *J. Hous. Built Environ.* 38, 2821–2850 (2023). doi: 10.1007/s10901-023-10067-5.
[x] Salazar, J., Guevara, J., Espinosa, M., Rivera, F. & Franco, J. F. Decarbonization of the Colombian building sector: Social network analysis of enabling stakeholders. *Buildings* 12, 1531 (2022). https://doi.org/10.3390/buildings12101531.
[xi] Sourani, A. & Sohail, M. Barriers to addressing sustainable construction in public procurement strategies. *Proc. Inst. Civil Eng. Eng. Sustainability* 164, 229–237 (2011). doi: 10.1680/ensu.2011.164.4.229.
[xii] Streimikiene, D. & Balezentis, T. Willingness to pay for renovation of multi-flat buildings and to share the costs of renovation. *Energies* 13, 2721 (2020). doi: 10.3390/en13112721.
[xiii] Carlander, J. & Thollander, P. Barriers to implementation of energy-efficient technologies in building construction projects—Results from a Swedish case study. *Resour. Environ. Sustain.* 11, 100097 (2023). https://doi.org/10.1016/j.resenv.2022.100097.

[xiv] Agyekum, K., Adinyira, E., Baiden, B., Ampratwum, G. & Duah, D. Barriers to the adoption of green certification of buildings: A thematic analysis of verbatim comments from built environment professionals. *J. Eng. Des. Technol.* 17, 1035–1055 (2019). https://doi.org/10.1108/JEDT-01-2019-0028.
[xv] Aiello, S. Addressing financial objections to sustainable design and construction. *J. Green Build.* 5, 67–77 (2010). https://doi.org/10.3992/jgb.5.4.67.
[xvi] Nguyen, H.-T., Skitmore, M., Gray, M., Zhang, X. & Olanipekun, A.O. Will green building development take off? An exploratory study of barriers to green building in Vietnam. *Resour. Conserv. Recycl.* 127, 8–20 (2017). doi: 10.1016/j.resconrec.2017.08.012.
[xvii] Waqar, A., Othman, I., Almujibah, H. R., Sajjad, M., Deifalla, A., Shafiq, N., Azab, M. & Qureshi, A. H. Overcoming Implementation Barriers in 3D Printing for Gaining Positive Influence Considering PEST Environment. *Ain Shams Eng. J.* 15, 102517 (2024). https://doi.org/10.1016/j.asej.2023.102517.

Supplementary References: Academic Literature

Source Literature for Review

Ababio, B., & Lu, W. (2023). Barriers and enablers of circular economy in construction: A multi-system perspective towards the development of a practical framework. CONSTRUCTION MANAGEMENT AND ECONOMICS, 41(1), 3–21. https://doi.org/10.1080/01446193.2022.2135750

Abdulai, S., Nani, G., Taiwo, R., Antwi-Afari, P., Zayed, T., & Sojobi, A. (2024). Modelling the relationship between circular economy barriers and drivers for sustainable construction industry. BUILDING AND ENVIRONMENT, 254. https://doi.org/10.1016/j.buildenv.2024.111388

Abdulsalam, R., Chan, M., Masrom, M., & Nawawi, A. (2024). Benefits and challenges of implementing green building development in Nigeria. BUILT ENVIRONMENT PROJECT AND ASSET MANAGEMENT, 14(3), 399–413. https://doi.org/10.1108/BEPAM-09-2022-0143

Abidin, N., & Powmya, A. (2024). Identifying barriers in constructing green buildings in Oman: A study on project contractors' experience. BUILT ENVIRONMENT PROJECT AND ASSET MANAGEMENT. https://doi.org/10.1108/BEPAM-02-2024-0044

Abougamil, R., Thorpe, D., & Heravi, A. (2023). Investigating the Source of Claims with the Importance of BIM Application on Reducing Construction Disputable Claims in KSA. BUILDINGS, 13(9). https://doi.org/10.3390/buildings13092219

Abougamil, R., Thorpe, D., & Heravi, A. (2024). An Investigation of BIM Advantages in Analysing Claims Procedures Related to the Extension of Time and Money in the KSA Construction Industry. BUILDINGS, 14(2). https://doi.org/10.3390/buildings14020426

Abreu, M. I., Pereira, A., & Gervásio, H. (2023). From a Techno-Economic towards a Socio-Technical Approach—A Review of the Influences and Policies on Home Energy Renovations' Decisions. Buildings, 13(3), 761. https://doi.org/10.3390/buildings13030761

Abuseif, M., Dupre, K., & Michael, R. (2022). Trees on buildings: Opportunities, challenges, and recommendations. BUILDING AND ENVIRONMENT, 225. https://doi.org/10.1016/j.buildenv.2022.109628

Addy, M., Adinyira, E., Danku, J., & Dadzoe, F. (2021). Impediments to the development of the green building market in sub-Saharan Africa: The case of Ghana. SMART AND SUSTAINABLE BUILT ENVIRONMENT, 10(2), 193–207. https://doi.org/10.1108/SASBE-12-2019-0170

Adekunye, O., & Oke, A. (2024). Challenges to the adoption of biomimicry as a sustainable approach in the nigerian construction industry. INTELLIGENT BUILDINGS INTERNATIONAL. https://doi.org/10.1080/17508975.2024.2370374

Adetooto, J., & Windapo, A. (2022). Concomitant Impediments to the Social Acceptance of Sandbag Technology for Sustainable and Affordable Housing Delivery: The Case of South Africa. BUILDINGS, 12(6). https://doi.org/10.3390/buildings12060859

Adhi, A., & Muslim, F. (2023). Development of Stakeholder Engagement Strategies to Improve Sustainable Construction Implementation Based on Lean Construction Principles in Indonesia. SUSTAINABILITY, 15(7). https://doi.org/10.3390/su15076053

Affonso, E., Branco, R., Menezes, O., Guedes, A., Chinelli, C., Haddad, A., & Soares, C. (2024). The Main Barriers Limiting the Development of Smart Buildings. BUILDINGS, 14(6). https://doi.org/10.3390/buildings14061726

Agapiou, A. (2022). Barriers to Offsite Construction Adoption: A Quantitative Study among Housing Associations in England. BUILDINGS, 12(3). https://doi.org/10.3390/buildings12030283

Aghimien, D., Aigbavboa, C., Aghimien, L., Thwala, W. D., & Ndlovu, L. (2020). Making a case for 3D printing for housing delivery in South Africa. International Journal of Housing Markets and Analysis, 13(4), 565–581. https://doi.org/10.1108/IJHMA-11-2019-0111

Aghimien, D., Oke, A., & Aigbavboa, C. (2019). Barriers of Building Maintenance in Private Tertiary Institutions in Nigeria (WOS:000559112200030). 788, 312–323. https://doi.org/10.1007/978-3-319-94199-8_30

Agyekum, K., Adinyira, E., Baiden, B., Ampratwum, G., & Duah, D. (2019). Barriers to the adoption of green certification of buildings A thematic analysis of verbatim comments from built environment professionals. JOURNAL OF ENGINEERING DESIGN AND TECHNOLOGY, 17(5), 1035–1055. https://doi.org/10.1108/JEDT-01-2019-0028

Agyekum, K., Opoku, A., Oppon, A., & Opoku, D. (2022). Obstacles to green building project financing: An empirical study in Ghana. INTERNATIONAL JOURNAL OF CONSTRUCTION MANAGEMENT, 22(15), 2922–2930. https://doi.org/10.1080/15623599.2020.1832182

Ahmad Huzaimi Abd Jamil & Mohamad Syazli Fathi. (2019). Contractual issues for Building Information Modelling (BIM)-based construction projects: An exploratory case study. IOP Conference Series. Materials Science and Engineering, 513(1). https://doi.org/10.1088/1757-899X/513/1/012035

Ahmed, A., Ge, T., Peng, J., Yan, W., Tee, B., & You, S. (2022). Assessment of the renewable energy generation towards net-zero energy buildings: A review. ENERGY AND BUILDINGS, 256. https://doi.org/10.1016/j.enbuild.2021.111755

Ahmed, S., & Sobuz, M. (2020). Challenges of implementing lean construction in the construction industry in Bangladesh. SMART AND SUSTAINABLE BUILT ENVIRONMENT, 9(2), 174–207. https://doi.org/10.1108/SASBE-02-2019-0018

Ahmed, S., Hossain, M., & Haq, I. (2021). Implementation of lean construction in the construction industry in Bangladesh: Awareness, benefits and challenges. INTERNATIONAL JOURNAL OF BUILDING PATHOLOGY AND ADAPTATION, 39(2), 368–406. https://doi.org/10.1108/IJBPA-04-2019-0037

Aiello, S. (2010). ADDRESSING FINANCIAL OBJECTIONS TO SUSTAINABLE DESIGN AND CONSTRUCTION. JOURNAL OF GREEN BUILDING, 5(4), 67–77. https://doi.org/10.3992/jgb.5.4.67

Akhatova, A., Kranzl, L., Schipfer, F., & Heendeniya, C. B. (2022). Agent-Based Modelling of Urban District Energy System Decarbonisation—A Systematic Literature Review. Energies, 15(2), 554. https://doi.org/10.3390/en15020554

Akinradewo, O., Aigbavboa, C., & Ngwenya, L. (2019). Overcoming the challenges encountered by construction industry SMEs in using insurance. IOP Conference Series. Materials Science and Engineering, 640(1). https://doi.org/10.1088/1757-899X/640/1/012027

Alakhali, A. K., Baarimah, A. O., Alkhadri, A. H., Gabir, A. A., Hasan, K., & Aawag Mohsen Alawag. (2024). Unlocking the Potential of BIM for Transformation in the Kenyan Construction Industry. The Institute of Electrical and Electronics Engineers, Inc. (IEEE) Conference Proceedings. https://doi.org/10.1109/ICETSIS61505.2024.10459431

Alaux, N., Truger, B., Hoxha, E., M Ruschi Mendes Saade, & Passer, A. (2022). Greenhouse gas reduction strategies for building materials: A reality check with the climate targets. IOP Conference Series. Earth and Environmental Science, 1078(1), 012050. https://doi.org/10.1088/1755-1315/1078/1/012050

Al-Awag, A. M., Alaloul, W. S., Liew, M. S., Baarimah, A. O., & Musarat Muhammad Ali. (2023). The potential role of industrialized building systems (IBS) in Malaysian sustainable construction: Awareness and barriers. AIP Conference Proceedings, 2608(1). https://doi.org/10.1063/5.0128035

Albeaino, G., & Gheisari, M. (2021). TRENDS, BENEFITS, AND BARRIERS OF UNMANNED AERIAL SYSTEMS IN THE CONSTRUCTION INDUSTRY: A SURVEY STUDY IN THE UNITED STATES. JOURNAL OF INFORMATION TECHNOLOGY IN CONSTRUCTION, 26, 84–111. https://doi.org/10.36680/j.itcon.2021.006

Alex Gonzalez Caceres. (2018). Shortcomings and Suggestions to the EPC Recommendation List of Measures: In-Depth Interviews in Six Countries. Energies, 11(10), n/a. https://doi.org/10.3390/en11102516

Alfaiz, S., Abd Karim, S., & Alashwal, A. (2021). Critical Success Factors of Green Building Retrofitting Ventures in Iraq. INTERNATIONAL JOURNAL OF SUSTAINABLE CONSTRUCTION ENGINEERING AND TECHNOLOGY, 12(1). https://doi.org/10.30880/ijscet.2021.12.01.002

Alghamdi, M. S., Beach, T. H., & Rezgui, Y. (2022). Reviewing the effects of deploying building information modelling (BIM) on the adoption of sustainable design in Gulf countries: A case study in Saudi Arabia. City, Territory and Architecture, 9(1). https://doi.org/10.1186/s40410-022-00160-7

Alhammadi, Y., Kineber, A., & Alhusban, M. (2024). Investigating Barriers to the Adoption of Energy Management Practices for Sustainable Construction Projects: SEM and ANN Approaches. CIVIL ENGINEERING JOURNAL-TEHRAN, 10(4), 1232–1253. https://doi.org/10.28991/CEJ-2024-010-04-015

Alhumayn, S., Chinyio, E., & Ndekugri, I. (2017). THE BARRIERS AND STRATEGIES OF IMPLEMENTING BIM IN SAUDI ARABIA (WOS:000576963300006). 169, 55–67. https://doi.org/10.2495/BIM170061

Ali, A. (2018). Development of a framework for sustainable construction waste management: A case study of three major Libyan cities. PQDT - UK & Ireland. https://ezproxy.bu.edu/login?qurl=https://www.proquest.com/docview/2341404735?accountid=9676&bdid=81940&_bd=8nhaLNoSRG9HH3k46m33MtOX%2FC0%3D

Alite, M., Abu-Omar, H., Agurcia, M. T., Jácome, M., Kenney, J., Tapia, A., & Siebel, M. (2023). Construction and demolition waste management in Kosovo: a survey of challenges and opportunities on the road to circular economy. Journal of Material Cycles and Waste Management, 25(2), 1191-1203.

Aliu, J., Oke, A., Odia, O., Akanni, P., Leo-Olagbaye, F., & Aigbavboa, C. (2024). Exploring the barriers to the adoption of environmental economic practices in the construction industry. MANAGEMENT OF ENVIRONMENTAL QUALITY. https://doi.org/10.1108/MEQ-01-2024-0053

Allen, S., Moorman, C., Peterson, M., Hess, G., & Moore, S. (2012). Overcoming socio-economic barriers to conservation subdivisions: A case-study of four successful communities. LANDSCAPE AND URBAN PLANNING, 106(3), 244–252. https://doi.org/10.1016/j.landurbplan.2012.03.012

Allwood, J., Ashby, M., Gutowski, T., & Worrell, E. (2011). Material efficiency: A white paper. RESOURCES CONSERVATION AND RECYCLING, 55(3), 362–381. https://doi.org/10.1016/j.resconrec.2010.11.002

Al-Otaibi, A., Bowan, P., Daiem, M., Said, N., Ebohon, J., Alabdullatief, A., Al-Enazi, E., & Watts, G. (2022). Identifying the Barriers to Sustainable Management of Construction and Demolition Waste in Developed and Developing Countries. SUSTAINABILITY, 14(13). https://doi.org/10.3390/su14137532

Alqahtani, F., Alsanad, A., Alsadan, A., Sherif, M., & Mohamed, A. (2022). Scrutinizing the Adoption of Integrated Project Delivery in the Kingdom of Saudi Arabia Construction Sector. BUILDINGS, 12(12). https://doi.org/10.3390/buildings12122144

Alsaeedi, F., Lafta, M. J., & Ahmed, A. (2020). State of Building Information Modelling (BIM) adoption in Iraq. IOP Conference Series. Materials Science and Engineering, 737(1). https://doi.org/10.1088/1757-899X/737/1/012007

AlSanad, S. (2015). Awareness, Drivers, Actions, and Barriers of Sustainable Construction in Kuwait (WOS:000380430700112). 118, 969–983. https://doi.org/10.1016/j.proeng.2015.08.538

Altarawneh, N., & Murtadha, T. (2023). Managing engineering challenges in the design and implementation of eco-friendly residential structures. RESULTS IN ENGINEERING, 19. https://doi.org/10.1016/j.rineng.2023.101363

Altassan, A., Othman, M., Elbeltagi, E., Abdelshakor, M., & Ehab, A. (2023). A Qualitative Investigation of the Obstacles Inherent in the Implementation of Building Information Modeling (BIM). BUILDINGS, 13(3). https://doi.org/10.3390/buildings13030700

Alwashah, Z., Sweis, G., Abu Hajar, H., Abu-Khader, W., & Sweis, R. (2024). Challenges to adopt digital construction technologies in the Jordanian construction industry. CONSTRUCTION INNOVATION-ENGLAND. https://doi.org/10.1108/CI-03-2023-0056

Al-Yami, A., & Sanni-Anibire, M. O. (2021). BIM in the Saudi Arabian construction industry: State of the art, benefit and barriers. International Journal of Building Pathology and Adaptation, 39(1), 33–47. https://doi.org/10.1108/IJBPA-08-2018-0065

Amade, B., Moneke, U., & Okorie, C. (2024). Modelling the Hindrances to Building Information Modelling Adoption on Construction Projects in Nigeria. JOURNAL OF CONSTRUCTION IN DEVELOPING COUNTRIES, 29(1), 23–46. https://doi.org/10.21315/jcdc-11-21-0188

Ambekar, S., Roy, D., Hiray, A., Prakash, A., & Patyal, V. (2022). Barriers to adoption of reverse logistics: A case of construction, real estate, infrastructure and project (CRIP) sectors. ENGINEERING CONSTRUCTION AND ARCHITECTURAL MANAGEMENT, 29(7), 2878–2902. https://doi.org/10.1108/ECAM-02-2021-0112

Amecke, H. (2012). The impact of energy performance certificates: A survey of German home owners. Energy Policy, 46, 4. https://doi.org/10.1016/j.enpol.2012.01.064

Ameh, J., Soyingbe, A., & Oyediran, O. (2019). ACCEPTABILITY AND USE OF INNOVATIVE BAMBOO PRODUCTS FOR THE CONSTRUCTION OF RESIDENTIAL BUILDINGS IN NIGERIA. INTERNATIONAL JOURNAL OF TECHNOLOGY, 10(4), 648–656. https://doi.org/10.14716/ijtech.v10i4.2574

Ameli, N., & Kammen, D. (2012). Clean energy deployment: Addressing financing cost. ENVIRONMENTAL RESEARCH LETTERS, 7(3). https://doi.org/10.1088/1748-9326/7/3/034008

Ametepey, O., Aigbavboa, C., & Ansah, K. (2015). Barriers to successful implementation of sustainable construction in the Ghanaian construction industry (WOS:000383740301106). 3, 1682–1689. https://doi.org/10.1016/j.promfg.2015.07.988

Ammar, A., Nassereddine, H., AbdulBaky, N., AbouKansour, A., Tannoury, J., Urban, H., & Schranz, C. (2022). Digital Twins in the Construction Industry: A Perspective of Practitioners and Building Authority. FRONTIERS IN BUILT ENVIRONMENT, 8. https://doi.org/10.3389/fbuil.2022.834671

Amoruso, G., Donevska, N., & Skomedal, G. (2018). German and Norwegian policy approach to residential buildings’ energy efficiency-a comparative assessment. ENERGY EFFICIENCY, 11(6), 1375–1395. https://doi.org/10.1007/s12053-018-9637-5

Ampatzidis, P., Bowyer, E., Coley, D., & Stephenson, V. (2023). Decarbonising at scale: Extracting strategic thinking from EPC and deprivation data. BUILDING SERVICES ENGINEERING RESEARCH & TECHNOLOGY, 44(6), 625–639. https://doi.org/10.1177/01436244231203193

Ana Mafalda Matos, João M P Q Delgado, & Guimarães, A. S. (2022). Linking Energy Poverty with Thermal Building Regulations and Energy Efficiency Policies in Portugal. Energies, 15(1), 329. https://doi.org/10.3390/en15010329

Anand, J., PhD, Liu, X., PhD, Wang, L., PhD, & Malhotra, M., PhD. (2024). An Assessment of Existing Barriers to Market Adoption of Thermal Energy Storage in Buildings Based on Interviews with Stakeholders. *ASHRAE Transactions*, *130*, 52–61.

Anand, V., Kadiri, V. L., & Putcha, C. (2023). Passive buildings: A state-of-the-art review. Journal of Infrastructure Preservation and Resilience, 4(1), 3. https://doi.org/10.1186/s43065-022-00068-z

Anastasiades Kostas, Joos, D., van den Berg Marc, Rinke, M., Blom, J., & Audenaert Amaryllis. (2023). Stakeholder perceptions on implementing design for disassembly and standardisation for heterogeneous construction components. Waste Management & Research, 41(8), 1372–1381. https://doi.org/10.1177/0734242X231154140

Andrade, I., Land, J., Gallardo, P., & Krumdieck, S. (2022). Application of the InTIME Methodology for the Transition of Office Buildings to Low Carbon—A Case Study. Sustainability, 14(19), 12053. https://doi.org/10.3390/su141912053

Anesi, J. (2016). HVAC's Role in Net-zero Buildings. *Air Conditioning, Heating & Refrigeration News*, *258*(13), 12.

Anne Sigrid Nordby. (2019). Barriers and opportunities to reuse of building materials in the Norwegian construction sector. IOP Conference Series. Earth and Environmental Science, 225(1). https://doi.org/10.1088/1755-1315/225/1/012061

Anonymous. (2012). Training for the Future: Arming Subcontractors With Advanced Tools Through Performance Simulation. *Energy Design Update*, *32*(10), 6.

Anonymous. (2014). Net-Zero Feasible in D.C. *Buildings*, *108*(6), 22.

Anonymous. (2023). RMI, USGBC audit concrete and steel in "Action on Embodied Carbon." *Concrete Products*, *76*(11), 24–24.

Anonymous. (2023b). Industry body reacts to new government net zero leadership. *H & V News*, 5–5.

Antai, I., Lenka, S., & Achtenhagen, L. (2024). Digital platforms and the construction supply chain: Trends and emerging themes in extant AEC research. CONSTRUCTION MANAGEMENT AND ECONOMICS. https://doi.org/10.1080/01446193.2024.2396592

Antonopoulos, C. A., Fuentes, T. L., McCord, K. H., Rackley, A. L. S., & Biswas, S. (2024). Regional assessment of household energy decision-making and technology adoption in the United States. Energy Policy, 185, 113940. https://doi.org/10.1016/j.enpol.2023.113940

Anzagira, L., Duah, D., Badu, E., Simpeh, E., & Marful, A. (2024). Stimulation strategies to promote green building uptake in developing countries: The case of Ghana. JOURNAL OF ENGINEERING DESIGN AND TECHNOLOGY, 22(3), 1012–1029. https://doi.org/10.1108/JEDT-12-2021-0719

Aranda, J., Tsitsanis, T., Georgopoulos, G., & Longares, J. M. (2023). Innovative Data-Driven Energy Services and Business Models in the Domestic Building Sector. Sustainability, 15(4), 3742. https://doi.org/10.3390/su15043742

Araya, R., Guillaumet, A., do Valle, A., Duque, M., Gonzalez, G., Cabrero, J., De Leon, E., Castro, F., Gutierrez, C., Negrao, J., Moya, L., & Guindos, P. (2022). Development of Sustainable Timber Construction in Ibero-America: State of the Art in the Region and Identification of Current International Gaps in the Construction Industry. SUSTAINABILITY, 14(3). https://doi.org/10.3390/su14031170

Arayici, Y., Coates, P., Koskela, L., Kagioglou, M., Usher, C., & O'Reilly, K. (2011). Technology adoption in the BIM implementation for lean architectural practice. Automation in Construction, 20(2), 189–195. https://doi.org/10.1016/j.autcon.2010.09.016

Arbulu, M., Oregi, X., Etxepare, L., & Hernandez-Minguillon, R. (2022). Barriers and challenges of the assessment framework of the Commission Recommendation (EU) 2019/786 on building renovation by European RTD projects. ENERGY AND BUILDINGS, 269. https://doi.org/10.1016/j.enbuild.2022.112267

Arent, D., Green, P., Abdullah, Z., Barnes, T., Bauer, S., Bernstein, A., Berry, D., Berry, J., Burrell, T., Carpenter, B., Cochran, J., Cortright, R., Curry-Nkansah, M., Denholm, P., Gevorian, V., Himmel, M., Livingood, B., Keyser, M., King, J., … Turchi, C. (2022). Challenges and opportunities in decarbonizing the US energy system. RENEWABLE & SUSTAINABLE ENERGY REVIEWS, 169. https://doi.org/10.1016/j.rser.2022.112939

Armistead, S., & Babaahmadi, A. (2024). Navigating regulatory challenges, technical performance and circular economy integration of mineral-based waste materials for sustainable construction: A mini review in the European context. WASTE MANAGEMENT & RESEARCH. https://doi.org/10.1177/0734242X241270973

Armstrong, A., Wright, C., Ashe, B., & Nielsen, H. (2017). Enabling innovation in building sustainability: Australia's National Construction Code (WOS:000404873600033). 180, 320–330. https://doi.org/10.1016/j.proeng.2017.04.191

Arogundade, S., Dulaimi, M., & Ajayi, S. (2024). Exploring the challenges impeding construction process carbon reduction in the UK. INTERNATIONAL JOURNAL OF CONSTRUCTION MANAGEMENT, 24(4), 422–431. https://doi.org/10.1080/15623599.2023.2257512

Arowolo, W., & Perez, Y. (2023). Rapid decarbonisation of Paris, Lyon and Marseille's power, transport and building sectors by coupling rooftop solar PV and electric vehicles. ENERGY FOR SUSTAINABLE DEVELOPMENT, 74, 196–214. https://doi.org/10.1016/j.esd.2023.04.002

Arzoyan, S. (2019). Endogenous energy efficiency improvements in large-scale retrofits to Swiss residential building stock (WOS:000561852800174). 1343. https://doi.org/10.1088/1742-6596/1343/1/012174

Asgarzadeh, M., Koga, T., Yoshizawa, N., Munakata, J., & Hirate, K. (2010). Investigating Green Urbanism; Building Oppressiveness. JOURNAL OF ASIAN ARCHITECTURE AND BUILDING ENGINEERING, 9(2), 555–562. https://doi.org/10.3130/jaabe.9.555

ASHRAE, Edelson, J., Cheslak, K., Miller, A., & Lyles, M. (2022). *Expanding the Role of Energy Codes to Meet Generational Shifts* (WOS:001057523300015). *128*, 129–136.

ASHRAE, Heizer, M., & Shelide, B. (2020). *Challenges to Statewide Net-Zero Energy Buildings: The Oregon Experience* (WOS:000784116000008). *126*, 29–37.

Assaad, R. (2024). Examining Low-Carbon Material Initiatives: Existing Policies, Impacts on the Procurement of Projects, Current Challenges, and Potential Solutions to Reduce Embodied Carbon in the Construction Industry. JOURNAL OF LEGAL AFFAIRS AND DISPUTE RESOLUTION IN ENGINEERING AND CONSTRUCTION, 16(2). https://doi.org/10.1061/JLADAH.LADR-1101

Assadiki, R., Merlin, G., Boileau, H., Buhé, C., & Belmir, F. (2022). Status and Prospects of Green Building in the Middle East and North Africa (MENA) Region with a Focus on the Moroccan Context. SUSTAINABILITY, 14(19). https://doi.org/10.3390/su141912594

Assessing Progress in Decarbonizing Spain's Building Stock; Indicators and Data Availability. (2024). Assessing Progress in Decarbonizing Spain's Building Stock. https://ezproxy.bu.edu/login?qurl=https://www.proquest.com/docview/3067835457?accountid=9676&bdid=77468&_bd=e%2FB%2FD4RsUYXAb65CwcIHfRuwgAM%3D

Assessment of technical and economical viability for large-scale conversion of single family residential buildings into zero energy buildings in Brazil: Climatic and cultural considerations. (n.d.). *63*.

Astmarsson, B., Jensen, P. A., & Maslesa, E. (2013). Sustainable renovation of residential buildings and the landlord/tenant dilemma. *Energy Policy*, *63*, 355–362.

Astudillo, M., Vaillancourt, K., Pineau, P., & Amor, B. (2017). Can the household sector reduce global warming mitigation costs? Sensitivity to key parameters in a TIMES techno-economic energy model. APPLIED ENERGY, 205, 486–498. https://doi.org/10.1016/j.apenergy.2017.07.130

Atmoko, D., Susilawati, C., & Goonetilleke, A. (2019). Improving Sustainability of Indonesian Public Building: Challenges and Policy Strategy (WOS:000770798200027). 131, 268–276. https://doi.org/10.1007/978-3-030-04293-6_27

Attoye, D. E., Kheira Anissa Tabet Aoul, & Hassan, A. (2022). Mandatory Policy, Innovations and the Renewable Energy Debate: A Case Study on Building Integrated Photovoltaics. Buildings, 12(7), 931. https://doi.org/10.3390/buildings12070931

Awareness of BIM adoption in Brunei. (2017). AIP Conference Proceedings, 1887(1). https://doi.org/10.1063/1.5003515

Aydin, Y., Mirzaei, P., & Akhavannasab, S. (2019). On the relationship between building energy efficiency, aesthetic features and marketability: Toward a novel policy for energy demand reduction. ENERGY POLICY, 128, 593–606. https://doi.org/10.1016/j.enpol.2018.12.036

Azar, E., Alaifan, B., Lin, M., Trepci, E., & El Asmar, M. (2021). Drivers of energy consumption in Kuwaiti buildings: Insights from a hybrid statistical and building performance simulation approach. Energy Policy, 150, 1. https://doi.org/10.1016/j.enpol.2021.112154

Azcarate-Aguerre, J. F., Conci, M., Zils, M., Hopkinson, P., & Klein, T. (2022). Building energy retrofit-as-a-service: A Total Value of Ownership assessment methodology to support whole life-cycle building circularity and decarbonisation. Construction Management and Economics, 40(9), 676–689. https://doi.org/10.1080/01446193.2022.2094434

Azeem, S., Naeem, M., & Waheed, A. (2020). Adoption of Green Building Practices in Pakistan: Barriers and Measures. In GREEN BUILDING IN DEVELOPING COUNTRIES: POLICY, STRATEGY AND TECHNOLOGY (WOS:000489143800013; pp. 199–215). https://doi.org/10.1007/978-3-030-24650-1_1110.1007/978-3-030-24650-1

Azharuddin, M. (2019). Integrated Carbon Emission Management for the United Arab Emirates Construction Industry. PQDT - UK & Ireland. https://ezproxy.bu.edu/login?qurl=https://www.proquest.com/docview/2579453311?accountid=9676&bdid=81940&_bd=GMM3ofvv4X%2BOu8KTDuqIkeJaLZY%3D

Babatunde, S., Udeaja, C., & Adekunle, A. (2021). Barriers to BIM implementation and ways forward to improve its adoption in the Nigerian AEC firms. INTERNATIONAL JOURNAL OF BUILDING PATHOLOGY AND ADAPTATION, 39(1), 48–71. https://doi.org/10.1108/IJBPA-05-2019-0047

Badiu, C., Barbuta-Misu, N., Chirita, M., Soare, I., Zlati, M., Fortea, C., & Antohi, V. (2024). Modelling the Impact of VAT Fiscality on Branch-Level Performance in the Construction Industry-Evidence from Romania. ECONOMIES, 12(2). https://doi.org/10.3390/economies12020030

Bahadorestani, A., Naderpajouh, N., & Sadiq, R. (2020). Planning for sustainable stakeholder engagement based on the assessment of conflicting interests in projects. JOURNAL OF CLEANER PRODUCTION, 242. https://doi.org/10.1016/j.jclepro.2019.118402

Bahidrah, S., & Korkmaz, K. A. (2017). Barriers to the implementation of the 2030 Saudi Visions Sustainable Construction Objectives. *Middle East Journal of Entrepreneurship, Leadership & Sustainable Development*, *1*(1), 33.

Balon, V., Bagul, A., & Kumar, R. (2024). Green Construction Supply Chain Barriers Assessment: Evidence from Indian Construction Industry. GLOBAL BUSINESS REVIEW. https://doi.org/10.1177/09721509241231107

Bao, Z., Lee, W. M., & Lu, W. (2020). Implementing on-site construction waste recycling in Hong Kong: Barriers and facilitators. Science of the Total Environment, 747, 141091.

Barbero, I., Rezgui, Y., & Petri, I. (2023). A European-wide exploratory study to analyse the relationship between training and energy efficiency in the construction sector. Environment Systems & Decisions, 43(3), 337–357. https://doi.org/10.1007/s10669-022-09891-x

Barut, T., & Selçuk, S. (2024). Holistic Approach to Niche Formation: A Case on Transition to Nearly Zero-Energy Buildings in Türkiye. BUILDINGS, 14(6). https://doi.org/10.3390/buildings14061565

Bashir, H., Al-Hawarneh, A., Haridy, S., Shamsuzzaman, M., & Aydin, R. (2024). Barriers to Implementing Environmental Sustainability in UAE Construction Project Management: Identification and Comparison of ISO 14001-Certified and Non-Certified Firms. SUSTAINABILITY, 16(16). https://doi.org/10.3390/su16166779

Beach, T. H., Hippolyte, J.-L., & Rezgui, Y. (2020). Towards the adoption of automated regulatory compliance checking in the built environment. Automation in Construction, 118, 1. https://doi.org/10.1016/j.autcon.2020.103285

Beach, T., Rezgui, Y., Li, H., & Kasim, T. (2015). A rule-based semantic approach for automated regulatory compliance in the construction sector. Expert Systems with Applications, 42(12), 5219–5231. https://doi.org/10.1016/j.eswa.2015.02.029

Belaid, F., & Massié, C. (2022). What are the salient factors determining the usage of heating energy sources in France? Evidence from a discrete choice model. ENERGY AND BUILDINGS, 273. https://doi.org/10.1016/j.enbuild.2022.112386

Belaïd, F., & Massié, C. (2023). Driving forward a low-carbon built environment: The impact of energy context and environmental concerns on building renovation. Energy Economics, 124, 106865.

Belay, S., Goedert, J., Woldesenbet, A., & Rokooei, S. (2021). Enhancing BIM implementation in the Ethiopian public construction sector: An empirical study. COGENT ENGINEERING, 8(1). https://doi.org/10.1080/23311916.2021.1886476

Bennett-Ness, J., & Nerval, C. (2023). Net zero target "will be missed." *Property Week*, 1–1.

Bertelsen, N., & Vad Mathiesen, B. (2020). EU-28 Residential Heat Supply and Consumption: Historical Development and Status. ENERGIES, 13(8). https://doi.org/10.3390/en13081894

Bertoldi, P., Economidou, M., Palermo, V., Benigna Boza-Kiss, & Todeschi, V. (2021). How to finance energy renovation of residential buildings: Review of current and emerging financing instruments in the EU. Wiley Interdisciplinary Reviews, 10(1), e384. https://doi.org/10.1002/wene.384

Bertolini, M. (2022). Energy Efficiency in Urban Context: An Overview of European-Funded Projects with the Analysis of an ELENA Case Study. SUSTAINABILITY, 14(17). https://doi.org/10.3390/su141710574

Biere-Arenas, R., Spairani-Berrio, S., Spairani-Berrio, Y., & Marmolejo-Duarte, C. (2021). One-Stop-Shops for Energy Renovation of Dwellings in Europe—Approach to the Factors That Determine Success and Future Lines of Action. Sustainability, 13(22), 12729. https://doi.org/10.3390/su132212729

Bijivemula, S., Sai, S., & Chepuri, A. (2024). A structural equation model of stakeholder roles in the implementation of green construction strategies in the Indian construction industry. INTERNATIONAL JOURNAL OF CONSTRUCTION MANAGEMENT, 24(5), 486–494. https://doi.org/10.1080/15623599.2023.2179568

Bilge Gokhan Celik, Abraham, Y. S., & Attaran, M. (2024). Unlocking Blockchain in Construction: A Systematic Review of Applications and Barriers. Buildings, 14(6), 1600. https://doi.org/10.3390/buildings14061600

Biskupski, R., Bolin, G., Nauclér, T., Sjödin, E., Smeets, B., & Spranzi, P. (2023). Refurbishing Europe: Igniting opportunities in the built environment. *McKinsey Insights*, n/a.

Björklund, M., von Malmborg, F., & Nordensvärd, J. (2023). Lessons learnt from 20+years of research on multilevel governance of energy-efficient and zero-carbon buildings in the European Union. ENERGY EFFICIENCY, 16(8). https://doi.org/10.1007/s12053-023-10178-6

Blomqvist, S., Glad, W., & Rohdin, P. (2022). Ten years of energy efficiency-Exploring the progress of barriers and drivers in the swedish residential and services sector. ENERGY REPORTS, 8, 14726–14740. https://doi.org/10.1016/j.egyr.2022.10.439

Blomqvist, S., Ödlund, L., & Rohdin, P. (2022). Understanding energy efficiency decisions in the building sector—A survey of barriers and drivers in Sweden. CLEANER ENGINEERING AND TECHNOLOGY, 9. https://doi.org/10.1016/j.clet.2022.100527

Bomberg, M., Wojcik, R., & Piotrowski, J. (2016). A concept of integrated environmental approach, Part 2: Integrated approach to rehabilitation. JOURNAL OF BUILDING PHYSICS, 39(6), 482–502. https://doi.org/10.1177/1744259115624940

Bortali, M., Rabouli, M., Yessari, M., Errouhi, A., Zejli, D., & Hajjaji, A. (2022). Regulatory framework for the beneficial reuse of dredged sediments as construction materials: A case study in Morocco (WOS:000870671600024). 66, 441–446. https://doi.org/10.1016/j.matpr.2022.06.316

Bragança, L. & María Concepción Verde Muniesa. (2023). Measuring Carbon in Cities and Their Buildings through Reverse Engineering of Life Cycle Assessment. Applied System Innovation, 6(5), 76. https://doi.org/10.3390/asi6050076

Braun, K., Kropp, C., & Boeva, Y. (2022). From Digital Design to Data-Assets: Competing Visions, Policy Projects, and Emerging Arrangements of Value Creation in the Digital Transformation of Construction. Historical Social Research, 47(3). https://ezproxy.bu.edu/login?qurl=https://www.proquest.com/docview/2755157937?accountid=9676&bdid=81940&_bd=sFQKHx3SdRxQk1jhzrNfFHZk9Xs%3D

Braungardt, S., Bürger, V., & Köhler, B. (2021). Carbon Pricing and Complementary Policies—Consistency of the Policy Mix for Decarbonizing Buildings in Germany. Energies, 14(21), 7143. https://doi.org/10.3390/en14217143

Brismark, J., Malmqvist, T., & Borgström, S. (2022). Climate Mitigation in the Swedish Single-Family Homes Industry and Potentials for LCA as Decision Support. Buildings, 12(5), 588. https://doi.org/10.3390/buildings12050588

Britton, J. S. (2018). The Role of the City-Scale in Energy Transitions: Heat Networks in England and Germany. PQDT - UK & Ireland. https://ezproxy.bu.edu/login?qurl=https://www.proquest.com/docview/2351205089?accountid=9676&bdid=77468&_bd=rlfzx0tfEW7RTaDa%2FVKM3SKKQ0s%3D

Broad, O., Hawker, G., & Dodds, P. E. (2020). Decarbonising the UK residential sector: The dependence of national abatement on flexible and local views of the future. Energy Policy, 140, 111321. https://doi.org/10.1016/j.enpol.2020.111321

Broft, R., Badi, S. M., & Pryke, S. (2016). Towards supply chain maturity in construction. Built Environment Project and Asset Management, 6(2), 187–204. https://doi.org/10.1108/BEPAM-09-2014-0050

Brown, D. (2018). Business models for residential retrofit in the UK: a critical assessment of five key archetypes. Energy Efficiency, 11(6), 1497–1517. https://doi.org/10.1007/s12053-018-9629-5

Brown, D., Sorrell, S., & Kivimaa, P. (2019). Worth the risk? An evaluation of alternative finance mechanisms for residential retrofit. ENERGY POLICY, 128, 418–430. https://doi.org/10.1016/j.enpol.2018.12.033

Bucea-Manea-Țoniș, R., Oliva Maria Dourado Martins, Ilic, D., Belous, M., Radu Bucea-Manea-Țoniș, Braicu, C., & Violeta-Elena Simion. (2021). Green and Sustainable Public Procurement—An Instrument for Nudging Consumer Behavior. A Case Study on Romanian Green Public Agriculture across Different Sectors of Activity. Sustainability, 13(1), 12. https://doi.org/10.3390/su13010012

Buchard Martin Visby, & Christensen, T. B. (2024). Business models for the reuse of construction and demolition waste. Waste Management & Research, 42(5), 359–371. https://doi.org/10.1177/0734242X231188023

Buchholz, M., & Lützkendorf, T. (2023). European building passports: Developments, challenges and future roles. Buildings & Cities, 4(1), 902. https://doi.org/10.5334/bc.355

Bui, T. T. P., Domingo, N., MacGregor, C., & Wilkinson, S. (2022). Zero carbon refurbishment for existing buildings: A literature review. IOP Conference Series. Earth and Environmental Science, 1101(2), 022017. https://doi.org/10.1088/1755-1315/1101/2/022017

Bürger, V., Hesse, T., Köhler, B., Palzer, A., & Engelmann, P. (2019). German Energiewendedifferent visions for a (nearly) climate neutral building sector in 2050. ENERGY EFFICIENCY, 12(1), 73–87. https://doi.org/10.1007/s12053-018-9660-6

Bushra Danish Talpur, Liuzzi, S., Rubino, C., Cannavale, A., & Martellotta, F. (2023). Life Cycle Assessment and Circular Building Design in South Asian Countries: A Review of the Current State of the Art and Research Potentials. Buildings, 13(12), 3045. https://doi.org/10.3390/buildings13123045

Caetano, N. S., Carvalho, R. R., Franco, F. R., Afonso, C. A. R., & Felgueiras, C. (2018). Sustainable engineering labs—A Portuguese perspective. Energy Procedia, 153, 455–460. https://doi.org/10.1016/j.egypro.2018.10.077

Caetano, N., Carvalho, R., Franco, F., Afonso, C., & Felgueiras, C. (2018). Sustainable engineering labs—A Portuguese perspective (WOS:000470993600077). 153, 455–460. https://doi.org/10.1016/j.egypro.2018.10.077

Calle Müller, C., Pradhananga, P., & ElZomor, M. (2024). Pathways to decarbonization, circular construction, and sustainability in the built environment. International Journal of Sustainability in Higher Education, 25(6), 1315–1332. https://doi.org/10.1108/IJSHE-09-2023-0400

Camarasa, C., Kalahasthi, L. K., Sanchez-Díaz, I., Rosado, L., Hennes, L., Bienge, K., & Hamilton, I. (2021). Energy-Efficient Retrofit Measures (EERM) in Residential Buildings: An Application of Discrete Choice Modelling. Buildings, 11(6), 257. https://doi.org/10.3390/buildings11060257

Cappello, C., Giuffrida, S., Maria Rosa Trovato, & Ventura, V. (2022). Environmental Identities and the Sustainable City. The Green Roof Prospect for the Ecological Transition. Sustainability, 14(19), 12005. https://doi.org/10.3390/su141912005

Carcassi, O. B., Salierno, R., Falcinelli, P. A., Paoletti, I. M., & Ben-Alon, L. (2024). Upscaling Natural Materials in Construction: Earthen, Fast-Growing, and Living Materials. Sustainability, 16(18), 7926. https://doi.org/10.3390/su16187926

Cardoso, V. E., Sanhudo, L., Silvestre, J. D., Almeida, M., & Costa, A. A. (2024). Challenges in the harmonisation and digitalisation of Environmental Product Declarations for construction products in the European context. *The International Journal of Life Cycle Assessment*, *29*(5), 759-788.

Carlander, J., & Thollander, P. (2023). Barriers to implementation of energy-efficient technologies in building construction projects—Results from a Swedish case study. RESOURCES ENVIRONMENT AND SUSTAINABILITY, 11. https://doi.org/10.1016/j.resenv.2022.100097

Carnero, P., & Calatayud, P. (2021). A Parametric Analysis for Short-Term Residential Electrification with Electric Water Tanks. The Case of Spain. SUSTAINABILITY, 13(21). https://doi.org/10.3390/su132112070

Cascone, S., Parisi, G., & Caponetto, R. (2024). BIM-Based Strategies for the Revitalization and Automated Management of Buildings: A Case Study. SUSTAINABILITY, 16(16). https://doi.org/10.3390/su16166720

Celik, Y., Barbero, I., Hodorog, A., Petri, I., & Rezgui, Y. (2024). Blockchain for Energy Efficiency Training in the Construction Industry. Education and Information Technologies, 29(1), 323–349. https://doi.org/10.1007/s10639-023-12261-y

Celoza, A., de Oliveira, D., & Leite, F. (2023). Qualitative Analysis of the Impact of Contracts on Information Management in AEC Projects. JOURNAL OF CONSTRUCTION ENGINEERING AND MANAGEMENT, 149(3). https://doi.org/10.1061/JCEMD4.COENG-12359

Challenges and drivers of BIM implementation in Sarawak construction industry. (2024). AIP Conference Proceedings, 2991(1). https://doi.org/10.1063/5.0199316

Chan, A., Darko, A., & Ameyaw, E. (2017). Strategies for Promoting Green Building Technologies Adoption in the Construction Industry-An International Study. SUSTAINABILITY, 9(6). https://doi.org/10.3390/su9060969

Chan, A., Darko, A., Ameyaw, E., & Owusu-Manu, D. (2017). Barriers Affecting the Adoption of Green Building Technologies. JOURNAL OF MANAGEMENT IN ENGINEERING, 33(3). https://doi.org/10.1061/(ASCE)ME.1943-5479.0000507

Chan, A., Darko, A., Olanipekun, A., & Ameyaw, E. (2018). Critical barriers to green building technologies adoption in developing countries: The case of Ghana. JOURNAL OF CLEANER PRODUCTION, 172, 1067–1079. https://doi.org/10.1016/j.jclepro.2017.10.235

Chan, D., Olawumi, T., & Ho, A. (2019). Perceived benefits of and barriers to Building Information Modelling (BIM) implementation in construction: The case of Hong Kong. JOURNAL OF BUILDING ENGINEERING, 25. https://doi.org/10.1016/j.jobe.2019.100764

Chan, E., Qian, Q., & Lam, P. (2009). The market for green building in developed Asian cities-the perspectives of building designers. ENERGY POLICY, 37(8), 3061–3070. https://doi.org/10.1016/j.enpol.2009.03.057

Chan, K., Schillereff, D. N., Baas, A. C., Chadwick, M. A., Main, B., Mulligan, M., O'Shea Francis T, Pearce, R., Smith, T. E., Arnout, van S., Tebbs Emma, & Thompson, J. (2021). Low-cost electronic sensors for environmental research: Pitfalls and opportunities. Progress in Physical Geography, 45(3), 305–338. https://doi.org/10.1177/0309133320956567

Chang, R., Soebarto, V., Zhao, Z., & Zillante, G. (2016). Facilitating the transition to sustainable construction: China's policies. JOURNAL OF CLEANER PRODUCTION, 131, 534–544. https://doi.org/10.1016/j.jclepro.2016.04.147

Chansomsak, S., & Vale, B. (2010). PROGRESSING PRACTICES OF SUSTAINABLE SCHOOL DESIGN. JOURNAL OF GREEN BUILDING, 5(2), 147–157. https://doi.org/10.3992/jgb.5.2.147

Chappell, K. (2023). Will the construction industry ever manage to totally ditch diesel? *Construction News*, 18–18.

Chaturvedi, P., Kumar, N., & Lamba, R. (2024). Finding the gaps in design strategies and technological advancements for net-zero energy buildings development in India. ENERGY & ENVIRONMENT. https://doi.org/10.1177/0958305X241256039

Cheekatamarla, P. (2022). Role of On-Site Generation in Carbon Emissions and Utility Bill Savings under Different Electric Grid Scenarios. Energies, 15(10), 3477. https://doi.org/10.3390/en15103477

Cheekatamarla, P., & Nawaz, K. (2022). Global Building Decarbonization Trends and Strategies. Energies, 15(22), 8402. https://doi.org/10.3390/en15228402

Chegut, A., Eichholtz, P., & Kok, N. (2019). The price of innovation: An analysis of the marginal cost of green buildings. JOURNAL OF ENVIRONMENTAL ECONOMICS AND MANAGEMENT, 98. https://doi.org/10.1016/j.jeem.2019.07.003

Chen, J., Su, Y., Si, H., & Chen, J. (2018). Managerial Areas of Construction and Demolition Waste: A Scientometric Review. International Journal of Environmental Research and Public Health, 15(11), 2350. https://doi.org/10.3390/ijerph15112350

Chen, L., Darko, A., Adegoriola, M., Chan, A., Yang, Y., & Tetteh, M. (2024). Challenges to energy retrofitting of existing office buildings in high-rise high-density cities: The case of Hong Kong. ENERGY AND BUILDINGS, 312. https://doi.org/10.1016/j.enbuild.2024.114220

Chen, X., Shuai, C., Chen, Z., & Zhang, Y. (2019). What are the root causes hindering the implementation of green roofs in urban China? SCIENCE OF THE TOTAL ENVIRONMENT, 654, 742–750. https://doi.org/10.1016/j.scitotenv.2018.11.051

Chen, X., Vand, B., & Baldi, S. (2024). Challenges and Strategies for Achieving High Energy Efficiency in Building Districts. Buildings, 14(6), 1839. https://doi.org/10.3390/buildings14061839

Chen, Y., Cai, X., Li, J., Lin, P., Song, H., Liu, G., Cao, D., & Ma, X. (2023). The values and barriers of BIM implementation combination evaluation based on stakeholder theory: A study in China. ENGINEERING CONSTRUCTION AND ARCHITECTURAL MANAGEMENT, 30(7), 2814–2836. https://doi.org/10.1108/ECAM-08-2020-0607

Chen, Z., Chen, J., Chen, Y., & Pedrycz, W. (2024). Construction metaverse: Application framework and adoption barriers. AUTOMATION IN CONSTRUCTION, 163. https://doi.org/10.1016/j.autcon.2024.105422

Cheng, V. (2020). De-carbonizing Hong Kong-What energy strategies are effective? IOP Conference Series. Earth and Environmental Science, 588(2). https://doi.org/10.1088/1755-1315/588/2/022045

Chi Dara & Caroline Hachem Vermette. (2022). Life Cycle Embodied Energy and Carbon Emissions of Panelized Wall Systems. The International Journal of Architectonic, Spatial, and Environmental Design, 17(1), 37. https://doi.org/10.18848/2325-1662/CGP/v17i01/37-56

Chileshe, N., Rameezdeen, R., Hosseini, M., & Lehmann, S. (2015). Barriers to implementing reverse logistics in South Australian construction organisations. SUPPLY CHAIN MANAGEMENT-AN INTERNATIONAL JOURNAL, 20(2), 179–204. https://doi.org/10.1108/SCM-10-2014-0325

Chmutina, K., & Rose, J. (2018). Building resilience: Knowledge, experience and perceptions among informal construction stakeholders. INTERNATIONAL JOURNAL OF DISASTER RISK REDUCTION, 28, 158–164. https://doi.org/10.1016/j.ijdrr.2018.02.039

Chowdhury, M., Sabrina, H., Zzaman, R., & Ul Islam, S. (2022). Green building aspects in Bangladesh: A study based on experts opinion regarding climate change. ENVIRONMENT DEVELOPMENT AND SUSTAINABILITY, 24(7), 9260–9284. https://doi.org/10.1007/s10668-021-01823-0

Chro Hama Radha. (2023). Retrofitting for Improving Indoor Air Quality and Energy Efficiency in the Hospital Building. Sustainability, 15(4), 3464. https://doi.org/10.3390/su15043464

Chukwu, I. (2018). Sustainability in the Nigerian Construction Industry: The Feasibility of Adopting Green Building Rating System. ProQuest Dissertations and Theses. https://ezproxy.bu.edu/login?qurl=https://www.proquest.com/docview/2162853228?accountid=9676&bdid=81940&_bd=nveXM1ypvQaux6JG%2BGEq0ccxDoI%3D

Chung-Camargo, K., González, J., Miguel Chen Austin, Carpino, C., Mora, D., & Arcuri, N. (2024). Advances in Retrofitting Strategies for Energy Efficiency in Tropical Climates: A Systematic Review and Analysis. Buildings, 14(6), 1633. https://doi.org/10.3390/buildings14061633

Cioara, T., Antal, M., Mihailescu, V., Antal, C., Anghel, I., & Mitrea, D. (2021). Blockchain-Based Decentralized Virtual Power Plants of Small Prosumers. IEEE ACCESS, 9, 29490–29504. https://doi.org/10.1109/ACCESS.2021.3059106

Clarke, J., Littlewood, J. R., & Karani, G. (2023). Developing Tools to Enable the UK Construction Industry to Adopt the Active Building Concept for Net Zero Carbon Buildings. Buildings, 13(2), 304. https://doi.org/10.3390/buildings13020304

Climate Adaptation and Resilience Across Scales; From Buildings to Cities. (2021). Climate Adaptation and Resilience Across Scales. https://ezproxy.bu.edu/login?qurl=https://www.proquest.com/docview/2581456170?accountid=9676&bdid=77468&_bd=rjwB5o7dRm1iQkbG3P6%2Fu8KX3sw%3D

Cohen, J., Rosado, L., & Gil, J. (2022). How is the construction sector addressing the Circular Economy? Lessons from current practices and perceptions in Argentina. IOP Conference Series. Earth and Environmental Science, 1078(1), 012008. https://doi.org/10.1088/1755-1315/1078/1/012008

Collins, D., Junghans, A., & Haugen, T. (2018). Green leasing in commercial real estate; The drivers and barriers for owners and tenants of sustainable office buildings. Journal of Corporate Real Estate, 20(4), 244–259. https://doi.org/10.1108/JCRE-01-2017-0003

Colombier, M., & Li, J. (2012). Shaping climate policy in the housing sector in northern Chinese cities. Climate Policy, 12(4), 453. https://doi.org/10.1080/14693062.2011.592665

Condotta, M., & Zatta, E. (2021). Reuse of building elements in the architectural practice and the European regulatory context: Inconsistencies and possible improvements. JOURNAL OF CLEANER PRODUCTION, 318. https://doi.org/10.1016/j.jclepro.2021.128413

Constantinou, S., Al-naemi, F., Hameed Alrashidi, Mallick, T., & Issa, W. (2024). A review on technological and urban sustainability perspectives of advanced building-integrated photovoltaics. Energy Science & Engineering, 12(3), 1265–1293. https://doi.org/10.1002/ese3.1639

Creutzig, F., Simoes, S., Leipold, S., Berrill, P., Azevedo, I., Edelenbosch, O., Fishman, T., Haberl, H., Hertwich, E., Krey, V., Lima, A., Makov, T., Mastrucci, A., Milojevic-Dupont, N., Nachtigall, F., Pauliuk, S., Silva, M., Verdolini, E., van Vuuren, D., … Wilson, C. (2024). Demand-side strategies key for mitigating material impacts of energy transitions. NATURE CLIMATE CHANGE. https://doi.org/10.1038/s41558-024-02016-z

Cui, H., & Xia, J. (2024). Research on the path of building carbon peak in China based on LMDI decomposition and GA-BP model. Environmental Science and Pollution Research, 31(15), 22694–22714. https://doi.org/10.1007/s11356-024-32591-9

Cupido, A., Baetz, B., Pujari, A., & Chidiac, S. (2010). EVALUATING INSTITUTIONAL GREEN BUILDING POLICIES: A Mixed-Methods Approach. JOURNAL OF GREEN BUILDING, 5(1), 115–131. https://doi.org/10.3992/jgb.5.1.115

D'Agostino, D., Tzeiranaki, S., Zangheri, P., & Bertoldi, P. (2021). Assessing Nearly Zero Energy Buildings (NZEBs) development in Europe. ENERGY STRATEGY REVIEWS, 36. https://doi.org/10.1016/j.esr.2021.100680

D'Oca, S., Ferrante, A., Ferrer, C., Pernetti, R., Gralka, A., Sebastian, R., & Veld, P. (2018). Technical, Financial, and Social Barriers and Challenges in Deep Building Renovation: Integration of Lessons Learned from the H2020 Cluster Projects. BUILDINGS, 8(12). https://doi.org/10.3390/buildings8120174

Da Silva, I. F. (2023). Promoting Sustainability in the Construction Sector: Assessment of the Reuse of Construction Materials. PQDT - Global. https://ezproxy.bu.edu/login?qurl=https://www.proquest.com/docview/3098796349?accountid=9676&bdid=81940&_bd=mheoW1sXjVeDvlLzs0dmR9sSn6o%3D

Dadar, A., Bolotin, S., Aslan Malsagov, & Oolakai, Z. (2019). Improving construction duration forecasts and management of construction operations. E3S Web of Conferences, 110, n/a. https://doi.org/10.1051/e3sconf/201911001078

Dalirazar, S., & Sabzi, Z. (2022). Barriers to sustainable development: Critical social factors influencing the sustainable building development based on Swedish experts' perspectives. SUSTAINABLE DEVELOPMENT, 30(6), 1963–1974. https://doi.org/10.1002/sd.2362

Dalirazar, S., & Sabzi, Z. (2023). Strategic analysis of barriers and solutions to development of sustainable buildings using PESTLE technique. INTERNATIONAL JOURNAL OF CONSTRUCTION MANAGEMENT, 23(1), 167–181. https://doi.org/10.1080/15623599.2020.1854931

Dallapiccola, M., Barchi, G., Adami, J., & Moser, D. (2021). The Role of Flexibility in Photovoltaic and Battery Optimal Sizing towards a Decarbonized Residential Sector. ENERGIES, 14(8). https://doi.org/10.3390/en14082326

Daly, D., Carr, C., Daly, M., McGuirk, P., Stanes, E., & Santala, I. (2023). Extending urban energy transitions to the mid-tier: Insights into energy efficiency from the management of HVAC maintenance in "mid-tier" office buildings. ENERGY POLICY, 174. https://doi.org/10.1016/j.enpol.2022.113415

Dams, B., Maskell, D., Shea, A., Allen, S., Cascione, V., & Walker, P. (2023). Upscaling bio-based construction: Challenges and opportunities. BUILDING RESEARCH AND INFORMATION, 51(7), 764–782. https://doi.org/10.1080/09613218.2023.2204414

Darko, A., Chan, A., Ameyaw, E., He, B., & Olanipekun, A. (2017). Examining issues influencing green building technologies adoption: The United States green building experts' perspectives. ENERGY AND BUILDINGS, 144, 320–332. https://doi.org/10.1016/j.enbuild.2017.03.060

Darko, A., Chan, A., Yang, Y., Shan, M., He, B., & Gou, Z. (2018). Influences of barriers, drivers, and promotion strategies on green building technologies adoption in developing countries: The Ghanaian case. JOURNAL OF CLEANER PRODUCTION, 200, 687–703. https://doi.org/10.1016/j.jclepro.2018.07.318

Dat Tien Doan, Albsoul, H., & GhaffarianHoseini, A. (2023). Enhancing construction waste management in New Zealand: Lessons from Hong Kong and other countries. Environmental Research Communications, 5(10), 102001. https://doi.org/10.1088/2515-7620/ad0105

Dauda, J., & Ajayi, S. (2022). Understanding the impediments to sustainable structural retrofit of existing buildings in the UK. JOURNAL OF BUILDING ENGINEERING, 60. https://doi.org/10.1016/j.jobe.2022.105168

Dawes, J. (2018). Trending toward zero carbon. *Grand Rapids Business Journal*, *36*(1), 1–4.

Dawes, Ruth. (2010). Building to Improve Energy Efficiency in England and Wales. Environmental Law Review. 12. 266-281. 10.1350/enlr.2010.12.4.101.

de Almeida, L., Esposito, F., & van Zeben, J. (2022). When indicators fail electricity policies: Pitfalls of the EU's retail energy market Barrier Index. Energy Policy, 165, 1. https://doi.org/10.1016/j.enpol.2022.112892

De Araujo, V. (2023). Timber construction as a multiple valuable sustainable alternative: Main characteristics, challenge remarks and affirmative actions. INTERNATIONAL JOURNAL OF CONSTRUCTION MANAGEMENT, 23(8), 1334–1343. https://doi.org/10.1080/15623599.2021.1969742

De Silva, G., Perera, B., & Rodrigo, M. (2019). Adaptive reuse of buildings: The case of Sri Lanka. JOURNAL OF FINANCIAL MANAGEMENT OF PROPERTY AND CONSTRUCTION, 24(1), 79–96. https://doi.org/10.1108/JFMPC-11-2017-0044

Deason, J., & Borgeson, M. (2019). Electrification of Buildings: Potential, Challenges, and Outlook. Current Sustainable / Renewable Energy Reports, 6(4), 131–139. https://doi.org/10.1007/s40518-019-00143-2

Deetjen, T., Conger, J., Leibowicz, B., & Webber, M. (2018). Review of climate action plans in 29 major US cities: Comparing current policies to research recommendations. SUSTAINABLE CITIES AND SOCIETY, 41, 711–727. https://doi.org/10.1016/j.scs.2018.06.023

Del Rio, D., Sovacool, B., & Griffiths, S. (2021). Culture, energy and climate sustainability, and smart home technologies: A mixed methods comparison of four countries. ENERGY AND CLIMATE CHANGE, 2. https://doi.org/10.1016/j.egycc.2021.100035

Del Rio, D., Sovacool, B., Foley, A., Griffiths, S., Bazilian, M., Kim, J., & Rooney, D. (2022a). Decarbonizing the ceramics industry: A systematic and critical review of policy options, developments and sociotechnical systems. RENEWABLE & SUSTAINABLE ENERGY REVIEWS, 157. https://doi.org/10.1016/j.rser.2022.112081

Del Rio, D., Sovacool, B., Foley, A., Griffiths, S., Bazilian, M., Kim, J., & Rooney, D. (2022b). Decarbonizing the glass industry: A critical and systematic review of developments, sociotechnical systems and policy options. RENEWABLE & SUSTAINABLE ENERGY REVIEWS, 155. https://doi.org/10.1016/j.rser.2021.111885

Dell'Anna, F., Marmolejo-Duarte, C., Bravi, M., & Bottero, M. (2022). A choice experiment for testing the energy-efficiency mortgage as a tool for promoting sustainable finance. Energy Efficiency, 15(5). https://doi.org/10.1007/s12053-022-10035-y

Delmastro, C., & Gargiulo, M. (2020). Capturing the long-term interdependencies between building thermal energy supply and demand in urban planning strategies. APPLIED ENERGY, 268. https://doi.org/10.1016/j.apenergy.2020.114774

Delponte, I., Bianco, V., & Costa, V. (2022). The Role of Non-Energy Impact Assessment in Boosting Energy Efficiency and Urban Regeneration Projects: The RenOnBill Project and Experiences from Liguria Region. Energies, 15(11), 4093. https://doi.org/10.3390/en15114093

Deng, W., Yang, T., Tang, L., & Tang, Y.-T. (2018). Barriers and policy recommendations for developing green buildings from local government perspective: A case study of Ningbo China. Intelligent Buildings International, 10(2), 61. https://doi.org/10.1080/17508975.2016.1248342

Design & development of system to manage, buy & sell construction and demolition waste. (2024). AIP Conference Proceedings, 3139(1). https://doi.org/10.1063/5.0224589

Desport, L., & Selosse, S. (2022). An overview of CO2 capture and utilization in energy models. RESOURCES CONSERVATION AND RECYCLING, 180. https://doi.org/10.1016/j.resconrec.2021.106150

Di Turi, S., Ronchetti, L., Sannino, R., Calabrese, N., & Iatauro, D. (2024). The energy renovation pathway to ZEB in Italy: Analysis of typical buildings and methodological aspects (WOS:001223831500022). 523. https://doi.org/10.1051/e3sconf/202452304002

Dialga, I., & Thi Hang Giang, L. (2017). Highlighting Methodological Limitations in the Steps of Composite Indicators Construction. Social Indicators Research, 131(2), 441. https://doi.org/10.1007/s11205-016-1263-z

Ding, Z., Fan, Z., Tam, V., Bian, Y., Li, S., Illankoon, I., & Moon, S. (2018). Green building evaluation system implementation. BUILDING AND ENVIRONMENT, 133, 32–40. https://doi.org/10.1016/j.buildenv.2018.02.012

Doak, A., Stanier, C., Anthony, J., & Udaykumar, H. (2022). Can heat-pumps provide routes to decarbonization of building thermal control in the US Midwest? ENERGY SCIENCE & ENGINEERING, 10(8), 2612–2621. https://doi.org/10.1002/ese3.1159

Doh, S. I. (2023). Adoption approaches for BIM implementation in construction projects: Jordan as a case study. AIP Conference Proceedings, 2688(1). https://doi.org/10.1063/5.0141978

Dolge, K., & Blumberga, D. (2023). Transitioning to Clean Energy: A Comprehensive Analysis of Renewable Electricity Generation in the EU-27. ENERGIES, 16(18). https://doi.org/10.3390/en16186415

Domenig, C., Scheller, F., Gunkel, P., Hermann, J., Bergaentzlé, C., Lopes, M., Barnes, J., & Mckenna, R. (2024). Overcoming the landlord-tenant dilemma: A techno-economic assessment of collective self-consumption for European multi-family buildings. ENERGY POLICY, 189. https://doi.org/10.1016/j.enpol.2024.114120

Dooley, K. (2017). Routines, Rigidity and Real Estate: Organisational Innovations in the Workplace. Sustainability, 9(6), 998. https://doi.org/10.3390/su9060998

Dorr, E., François, C., Poulhès, A., & Wurtz, A. (2022). A life cycle assessment method to support cities in their climate change mitigation strategies. SUSTAINABLE CITIES AND SOCIETY, 85. https://doi.org/10.1016/j.scs.2022.104052

Downing, L., & Hsu, D. (2024). Decarbonizing Affordable Housing in New York City: Options and Obstacles to Scale Up Deep Energy Retrofits. CASE STUDIES IN THE ENVIRONMENT, 8(1). https://doi.org/10.1525/cse.2024.2253451

Drummond, P., & Ekins, P. (2017). Cost-effective decarbonization in the EU: an overview of policy suitability. Climate Policy, 17(sup1), 51. https://doi.org/10.1080/14693062.2016.1258634

Durdyev, S., Koc, K., Tleuken, A., Budayan, C., Ekmekcioglu, O., & Karaca, F. (2023). Barriers to circular economy implementation in the construction industry: Causal assessment model. ENVIRONMENT DEVELOPMENT AND SUSTAINABILITY. https://doi.org/10.1007/s10668-023-04061-8

Durdyev, S., Mbachu, J., Thurnell, D., Zhao, L., & Hosseini, M. (2021). BIM Adoption in the Cambodian Construction Industry: Key Drivers and Barriers. ISPRS INTERNATIONAL JOURNAL OF GEO-INFORMATION, 10(4). https://doi.org/10.3390/ijgi10040215

Durdyev, S., Zavadskas, E., Thurnell, D., Banaitis, A., & Ihtiyar, A. (2018). Sustainable Construction Industry in Cambodia: Awareness, Drivers and Barriers. SUSTAINABILITY, 10(2). https://doi.org/10.3390/su10020392

E Yu Gorbachevskaya, Bezrukikh, O. A., & Safronov, D. A. (2021). Wooden house building market: Technological problems in development issues. IOP Conference Series. Earth and Environmental Science, 751(1). https://doi.org/10.1088/1755-1315/751/1/012090

Ebekozien, A., Aigbavboa, C., Adekunle, S., Samsurijan, M., Aliu, J., Arthur-Aidoo, B., & Amadi, G. (2024). Smart contract applications in the built environment: How prepared are Nigerian construction stakeholders? FRONTIERS OF ENGINEERING MANAGEMENT, 11(1), 50–61. https://doi.org/10.1007/s42524-023-0275-z

Ebekozien, A., Ayo-Odifiri, S., Nwaole, A., Ibeabuchi, A., & Uwadia, F. (2022). Barriers in Nigeria's public hospital green buildings implementation initiatives. JOURNAL OF FACILITIES MANAGEMENT, 20(4), 586–605. https://doi.org/10.1108/JFM-01-2021-0009

Ebekozien, A., Ikuabe, M., Awo-Osagie, A., Aigbavboa, C., & Ayo-Odifiri, S. (2022). Model for promoting green certification of buildings in developing nations: A case study of Nigeria. PROPERTY MANAGEMENT, 40(1), 118–136. https://doi.org/10.1108/PM-05-2021-0033

Edelson, J., & Miller, A., PE. (2019). Taking the (Fuel) Blinders off Energy Codes Part 2: Metrics and Mechanics in the Modern Era. *ASHRAE Transactions*, *125*, 461–469.

Eguiarte, O., Garrido-Marijuán, A., De Agustín-Camacho, P., del Portillo, L., & Romero-Amorrortu, A. (2020). Energy, Environmental and Economic Analysis of Air-to-Air Heat Pumps as an Alternative to Heating Electrification in Europe. ENERGIES, 13(15). https://doi.org/10.3390/en13153939

Ehis Lawrence Onus, Chinyio, E., & Daniel, E. I. (2024). 'Stakeholder Perceptions' of the Impacts of Climatic Features on Residents and Residences: A UK Study. Atmosphere, 15(7), 791. https://doi.org/10.3390/atmos15070791

Eichhammer, W., Ragwitz, M., & Schlomann, B. (2013). Financing instruments to promote energy efficiency and renewables in times of tight public budgets. Energy & Environment, 24(1-2), 1-26.

Eley, C. (2016). Design Professional's Guide to Zero Net Energy Buildings. Design Professional's Guide to Zero Net Energy Buildings. https://ezproxy.bu.edu/login?qurl=https://www.proquest.com/docview/2134057843?accountid=9676&bdid=77468&_bd=l7df1NGfV%2F8QHPpMjINLiYb0GHw%3D

Elliott, T., Geske, J., & Green, R. (2022). Business Models for Active Buildings. Energies, 15(19), 7389. https://doi.org/10.3390/en15197389

Emre Caner Akcay. (2023). Barriers to Undertaking Green Building Projects in Developing Countries: A Turkish Perspective. Buildings, 13(4), 841. https://doi.org/10.3390/buildings13040841

Energy Retrofitting Opportunities Using Renewable Materials—Comparative Analysis of the Current Frameworks in Bosnia-Herzegovina and Slovenia. (2021). Sustainability, 13(2), 603. https://doi.org/10.3390/su13020603

Enhancing the appearance of Building Integrated Photovoltaics (BIPV) through the application of traditional symbols: A case study. (2022). AIP Conference Proceedings, 2681(1). https://doi.org/10.1063/5.0114941

Enshassi, A., & AbUHamra, L. (2017). Challenges to the Utilization of BIM in the Palestinian Construction Industry. ISARC. Proceedings of the International Symposium on Automation and Robotics in Construction, 34. https://ezproxy.bu.edu/login?qurl=https://www.proquest.com/docview/1943517356?accountid=9676&bdid=81940&_bd=Ujcg5QNgXlI5Hhkd5MFFmuapEVs%3D

Eon, C., Breadsell, J. K., Byrne, J., & Morrison, G. M. (2020). The Discrepancy between As-Built and As-Designed in Energy Efficient Buildings: A Rapid Review. Sustainability, 12(16), 6372. https://doi.org/10.3390/su12166372

European Union: Opening remarks by Executive Vice-President Timmermans and Commissioner Simson at the press conference Building a Climate Neutral Europe. (2020). Asia News Monitor. https://ezproxy.bu.edu/login?qurl=https://www.proquest.com/docview/2450816125?accountid=9676&bdid=77468&_bd=Qa%2BJ3qLUBju58UMtetdHzcKBBAg%3D

Eveloy, V., & Ayou, D. S. (2019). Sustainable District Cooling Systems: Status, Challenges, and Future Opportunities, with Emphasis on Cooling-Dominated Regions. Energies, 12(2), 235. https://doi.org/10.3390/en12020235

Eyre, N., Fawcett, T., Topouzi, M., Killip, G., Oreszczyn, T., & Rosenow, J. (2023). Fabric first: Is it still the right approach? Buildings & Cities, 4(1), 965. https://doi.org/10.5334/bc.388

Eze, E. C., Sofolahan, O., & Omoboye, O. G. (2023). Assessment of barriers to the adoption of sustainable building materials (SBM) in the construction industry of a developing country. Frontiers in Engineering and Built Environment, 3(3), 153–166. https://doi.org/10.1108/FEBE-07-2022-0029

Eze, E., Sofolahan, O., Uzoma, C., Ameyaw, E., & Omoboye, O. (2024). Impediments to building information modelling-enabled construction waste management in Nigeria. BUILT ENVIRONMENT PROJECT AND ASSET MANAGEMENT. https://doi.org/10.1108/BEPAM-12-2023-0217

Faber, A., & Hoppe, T. (2013). Co-constructing a sustainable built environment in the Netherlands—Dynamics and opportunities in an environmental sectoral innovation system. *Energy Policy*, *52*, 628–638.

Farahani, A., & Dalenbäck, J. (2019). Optimizing the Life Cycle Costs of Building Components with Regard to Energy Renovation (WOS:000675599300023). 265–274. https://doi.org/10.1007/978-3-030-00662-4_23

Farouk, A. M., Zulhisham, A. Z., Lee, Y. S., Mohammad Sadra Rajabi, & Rahman, R. A. (2023). Factors, Challenges and Strategies of Trust in BIM-Based Construction Projects: A Case Study in Malaysia. Infrastructures, 8(1), 13. https://doi.org/10.3390/infrastructures8010013

Fathalizadeh, A., Hosseini, M., Vaezzadeh, S., Edwards, D., Martek, I., & Shooshtarian, S. (2022). Barriers to sustainable construction project management: The case of Iran. SMART AND SUSTAINABLE BUILT ENVIRONMENT, 11(3), 717–739. https://doi.org/10.1108/SASBE-09-2020-0132

Fawcett, T., & Topouzi, M. (2019). What buildings policy might look like if we took climate change seriously. IOP Conference Series. Earth and Environmental Science, 329(1). https://doi.org/10.1088/1755-1315/329/1/012004

Fayez Musa, F. S. (2017). Communicating Sustainability: A Case Study Exploring Jordan Green Building Council's Sustainability Communication Techniques. ProQuest Dissertations and Theses. https://ezproxy.bu.edu/login?qurl=https://www.proquest.com/docview/1929640900?accountid=9676&bdid=81940&_bd=WsFNCAV4gVP320NcxSEEII2uyqg%3D

Feige, A., Wallbaumand, H., & Krank, S. (2011). Harnessing stakeholder motivation: Towards a Swiss sustainable building sector. BUILDING RESEARCH AND INFORMATION, 39(5), 504–517. https://doi.org/10.1080/09613218.2011.589788

Feng, L., Liu, J., Wang, Z., & Hong, Y. (2024). Navigating compliance complexity: Insights from the MOA framework in international construction. ENGINEERING CONSTRUCTION AND ARCHITECTURAL MANAGEMENT. https://doi.org/10.1108/ECAM-02-2024-0163

Fernandes, J., & Ferrão, P. (2023). A New Framework for Circular Refurbishment of Buildings to Operationalize Circular Economy Policies. Environments, 10(3), 51. https://doi.org/10.3390/environments10030051

Fernando, S., Hansen, E., Kozak, R., & Sinha, A. (2018). Organizational cultural compatibility of engineered wood products manufacturers and building specifiers in the Pacific Northwest. ARCHITECTURAL ENGINEERING AND DESIGN MANAGEMENT, 14(5), 398–410. https://doi.org/10.1080/17452007.2018.1491384

Ferreira, M., Morgado, C., & Estellita, M. (2024). Organizations and stakeholders' roles and influence on implementing sustainability requirements in construction projects. HELIYON, 10(1). https://doi.org/10.1016/j.heliyon.2023.e23762

Fina, B., Roberts, M., Auer, H., Bruce, A., & MacGill, I. (2021). Exogenous influences on deployment and profitability of photovoltaics for self-consumption in multi-apartment buildings in Australia and Austria. APPLIED ENERGY, 283. https://doi.org/10.1016/j.apenergy.2020.116309

Fiore, P., Donnarumma, G., Falce, C., Emanuela D'Andria, & Sicignano, C. (2020). An AHP-Based Methodology for Decision Support in Integrated Interventions in School Buildings. Sustainability, 12(23), 10181. https://doi.org/10.3390/su122310181

Fisch-Romito, V., Guivarch, C., Creutzig, F., Minx, J. C., & Callaghan, M. W. (2021). Systematic map of the literature on carbon lock-in induced by long-lived capital. Environmental Research Letters, 16(5). https://doi.org/10.1088/1748-9326/aba660

Fitriani, H., & Ajayi, S. (2023). Barriers to sustainable practices in the Indonesian construction industry. JOURNAL OF ENVIRONMENTAL PLANNING AND MANAGEMENT, 66(10), 2028–2050. https://doi.org/10.1080/09640568.2022.2057281

Foliente, G., & Seo, S. (2012). Modelling building stock energy use and carbon emission scenarios. Smart and Sustainable Built Environment, 1(2), 118. https://doi.org/10.1108/20466091211260578

Forsberg, M. & Clarice Bleil de Souza. (2021). Implementing Regenerative Standards in Politically Green Nordic Social Welfare States: Can Sweden Adopt the Living Building Challenge? Sustainability, 13(2), 738. https://doi.org/10.3390/su13020738

Fotiou, T., Capros, P., & Fragkos, P. (2022). Policy Modelling for Ambitious Energy Efficiency Investment in the EU Residential Buildings. ENERGIES, 15(6). https://doi.org/10.3390/en15062233

Fotiou, T., de Vita, A., & Capros, P. (2019). Economic-Engineering Modelling of the Buildings Sector to Study the Transition towards Deep Decarbonisation in the EU. ENERGIES, 12(14). https://doi.org/10.3390/en12142745

Fotiou, T., Fragkos, P., & Zisarou, E. (2024). Decarbonising the EU Buildings|Model-Based Insights from European Countries. Climate, 12(6), 85. https://doi.org/10.3390/cli12060085

Fournier, E., Federico, F., Cudd, R., Pincetl, S., Ricklefs, A., Costa, M., Jerrett, M., & Garcia-Gonzales, D. (2022). Net GHG emissions and air quality outcomes from different residential building electrification pathways within a California disadvantaged community. SUSTAINABLE CITIES AND SOCIETY, 86. https://doi.org/10.1016/j.scs.2022.104128

Franco, D., Macke, J., Cotton, D., Paço, A., Segers, J.-P., & Franco, L. (2022). Student energy-saving in higher education tackling the challenge of decarbonisation. International Journal of Sustainability in Higher Education, 23(7), 1648–1666. https://doi.org/10.1108/IJSHE-10-2021-0432

Franco, M., Pawar, P., & Wu, X. (2021). Green building policies in cities: A comparative assessment and analysis. ENERGY AND BUILDINGS, 231. https://doi.org/10.1016/j.enbuild.2020.110561

Franzini, F., Toivonen, R., & Toppinen, A. (2018). Why Not Wood? Benefits and Barriers of Wood as a Multistory Construction Material: Perceptions of Municipal Civil Servants from Finland. Buildings, 8(11), 159. https://doi.org/10.3390/buildings8110159

Fregonara, E., & Rubino, I. (2021). Buildings' energy performance, green attributes and real estate prices: Methodological perspectives from the European literature. Aestimum, 79, 43–73. https://doi.org/10.36253/aestim-10785

Friedman, R. A. (2018). Taking the Measure of Benchmarking. *Multi - Housing News*, *52*(8), 12–13.

Friege, J., Holtz, G., & Chappin, É. (2016). Exploring Homeowners' Insulation Activity. JASSS-THE JOURNAL OF ARTIFICIAL SOCIETIES AND SOCIAL SIMULATION, 19(1). https://doi.org/10.18564/jasss.2941

Frischknecht, R., Balouktsi, M., Lützkendorf, T., Aumann, A., Birgisdottir, H., Elmar Grosse Ruse, Hollberg, A., Kuittinen, M., Lavagna, M., Lupišek, A., Passer, A., Peuportier, B., Ramseier, L., Röck, M., Trigaux, D., & Vancso, D. (2019). Environmental benchmarks for buildings: Needs, challenges and solutions—71st LCA forum, Swiss Federal Institute of Technology, Zürich, 18 June 2019. The International Journal of Life Cycle Assessment, 24(12), 2272–2280. https://doi.org/10.1007/s11367-019-01690-y

Fufa, S., Brown, M., Hauge, Å., Johnsen, S., & Fjellheim, K. (2023). User perspectives on reuse of construction products in Norway: Results of a national survey. JOURNAL OF CLEANER PRODUCTION, 408. https://doi.org/10.1016/j.jclepro.2023.137067

Fuinhas, J. A., Matheus Koengkan, Silva, N., Kazemzadeh, E., Auza, A., Santiago, R., Teixeira, M., & Osmani, F. (2022). The Impact of Energy Policies on the Energy Efficiency Performance of Residential Properties in Portugal. Energies, 15(3), 802. https://doi.org/10.3390/en15030802

Fulvio Re Cecconi, & Rampini, L. (2023). Data driven economic scenarios for retrofitting residential buildings in a northern Italian region. IOP Conference Series. Earth and Environmental Science, 1196(1), 012113. https://doi.org/10.1088/1755-1315/1196/1/012113

Gaffar, R. A. (2016). Assess the Level of Public Awareness in UAE - Green Roof & Facades (Benefits & Obstacles). PQDT - Global. https://ezproxy.bu.edu/login?qurl=https://www.proquest.com/docview/2878260949?accountid=9676&bdid=81940&_bd=0JkKIOnM08i37fwcM9QbaquY3do%3D

Galimshina, A., Moustapha, M., Hollberg, A., Lasvaux, S., Sudret, B., & Habert, G. (2024). Strategies for robust renovation of residential buildings in Switzerland. Nature Communications, 15(1), 2227. https://doi.org/10.1038/s41467-024-46305-9

Galvin, R. (2021). Identifying possible drivers of rebound effects and reverse rebounds among households with rooftop photovoltaics. RENEWABLE ENERGY FOCUS, 38, 71–83. https://doi.org/10.1016/j.ref.2021.06.002

Galvin, R. (2024). Deep energy efficiency renovation of Germany's residential buildings: Is this as economically viable as Germany's policymakers and popular promoters often claim? Energy Efficiency, 17(5), 47. https://doi.org/10.1007/s12053-024-10227-8

Gamil, Y., & Rahman, I. (2019). Awareness and challenges of building information modelling (BIM) implementation in the Yemen construction industry. JOURNAL OF ENGINEERING DESIGN AND TECHNOLOGY, 17(5), 1077–1084. https://doi.org/10.1108/JEDT-03-2019-0063

Gan, X., Chang, R., & Wen, T. (2018). Overcoming barriers to off-site construction through engaging stakeholders: A two-mode social network analysis. JOURNAL OF CLEANER PRODUCTION, 201, 735–747. https://doi.org/10.1016/j.jclepro.2018.07.299

Gan, X., Chang, R., Zuo, J., Wen, T., & Zillante, G. (2018). Barriers to the transition towards off-site construction in China: An Interpretive structural modeling approach. JOURNAL OF CLEANER PRODUCTION, 197, 8–18. https://doi.org/10.1016/j.jclepro.2018.06.184

Gan, X., Liu, L., Wen, T., & Webber, R. (2022). Modelling interrelationships between barriers to adopting green building technologies in China's rural housing via grey-DEMATEL. TECHNOLOGY IN SOCIETY, 70. https://doi.org/10.1016/j.techsoc.2022.102042

Garces, P., Pires, C., Costa, J., Jorge, S., Catalao-Lopes, M., & Alventosa, A. (2022). Disentangling Housing Supply to Shift towards Smart Cities: Analysing Theoretical and Empirical Studies. SMART CITIES, 5(4), 1488–1507. https://doi.org/10.3390/smartcities5040076

Gasue, R., Aklashie, S., Dompey, A., Agyekum, K., & Opoku, D. (2024). Implementing materials passports in the construction industry: Empirical evidence from Ghana. INTERNATIONAL JOURNAL OF BUILDING PATHOLOGY AND ADAPTATION. https://doi.org/10.1108/IJBPA-01-2024-0007

Geh, N., Emuze, F., & Das, D. (2022). Barriers to the deployment of solar photovoltaic in public universities in South Africa: A Delphi study. INTERNATIONAL JOURNAL OF BUILDING PATHOLOGY AND ADAPTATION. https://doi.org/10.1108/IJBPA-11-2021-0147

Geh, N., Emuze, F., & Das, D. (2023). Solar photovoltaic deployment acceleration model to advance the sustainability of buildings in public universities in South Africa. ENERGY AND BUILDINGS, 284. https://doi.org/10.1016/j.enbuild.2023.112855

Geissler, S., & Spitzbart, C. (2010). Sustainable buildings in Austria—Performance indicators and implications on the construction industry (WOS:000281122400046). 128, 539–550. https://doi.org/10.2495/ARC100461

Geissler, S., Charalambides, A. G., & Hanratty, M. (2019). Public Access to Building Related Energy Data for Better Decision Making in Implementing Energy Efficiency Strategies: Legal Barriers and Technical Challenges. Energies, 12(10), n/a. https://doi.org/10.3390/en12102029

Gembali, V., Kumar, A., & Sarma, P. (2024). Analysis and influence mapping of socio-technical challenges for developing decarbonization and circular economy practices in the construction and building industry. ANNALS OF OPERATIONS RESEARCH. https://doi.org/10.1007/s10479-024-05864-2

Geng, J., Huang, Y., Li, X., & Zhang, Y. (2023). Overcoming Barriers to the Adoption of Recycled Construction Materials: A Comprehensive PEST Analysis and Tailored Strategies. SUSTAINABILITY, 15(19). https://doi.org/10.3390/su151914635

Geng, Y., Dong, H., Xue, B., & Fu, J. (2012). An Overview of Chinese Green Building Standards. SUSTAINABLE DEVELOPMENT, 20(3), 211–221. https://doi.org/10.1002/sd.1537

Georges, L., Selvnes, E., Heide, V., & Mathisen, H. M. (2019). Energy efficiency of strategies to enable temperature zoning during winter in highly-insulated residential buildings equipped with balanced mechanical ventilation. IOP Conference Series. Earth and Environmental Science, 352(1). https://doi.org/10.1088/1755-1315/352/1/012057

Ghaleb, B., Abbasi, S., & Asif, M. (2023). Application of solar PV in the building sector: Prospects and barriers in the GCC region. ENERGY REPORTS, 9, 3932–3942. https://doi.org/10.1016/j.egyr.2023.02.085

Ghansah, F. A., De-Graft, O.-M., Ayarkwa, J., Edwards, D. J., & Hosseini, M. R. (2021). Exploration of latent barriers inhibiting project management processes in adopting smart building technologies (SBTs) in the developing countries. Construction Innovation, 21(4), 685–707. https://doi.org/10.1108/CI-07-2020-0116

Gharaibeh, L., Matarneh, S., Eriksson, K., & Lantz, B. (2022). An Empirical Analysis of Barriers to Building Information Modelling (BIM) Implementation in Wood Construction Projects: Evidence from the Swedish Context. BUILDINGS, 12(8). https://doi.org/10.3390/buildings12081067

Gholami, H., Røstvik, H. N., & Steemers, K. (2021). The Contribution of Building-Integrated Photovoltaics (BIPV) to the Concept of Nearly Zero-Energy Cities in Europe: Potential and Challenges Ahead. Energies, 14(19), 6015. https://doi.org/10.3390/en14196015

Giesekam, J., Barrett, J. R., & Taylor, P. (2016). Construction sector views on low carbon building materials. Building Research and Information, 44(4), 423. https://doi.org/10.1080/09613218.2016.1086872

Gillott, C., Davison, B., & Densley Tingley, D. (2022). Drivers, barriers and enablers: Construction sector views on vertical extensions. Building Research & Information, 50(8), 909–923. https://doi.org/10.1080/09613218.2022.2087173

Giostra, S., Masera, G., & Monteiro, R. (2022). Solar Typologies: A Comparative Analysis of Urban Form and Solar Potential. Sustainability, 14(15), 9023. https://doi.org/10.3390/su14159023

Global Green USA, G. G. (2007). Blueprint for Greening Affordable Housing. Blueprint for Greening Affordable Housing. https://ezproxy.bu.edu/login?qurl=https://www.proquest.com/docview/2131948619?accountid=9676&bdid=77468&_bd=frB3oCSS6Ib80%2FVOs12GeibwUqA%3D

Glorianne Borg Axisa, & Borg, R. P. (2024). Climate change adaptation in Malta: Assessment of skills in the built environment. International Journal of Disaster Resilience in the Built Environment, 15(4), 668–679. https://doi.org/10.1108/IJDRBE-10-2023-0116

Gokarakonda, S., & Kumar, A. (2016). Passive Architectural Design Index applied to vernacular and passive buildings. *International Journal of Environmental Studies*, *73*(4), 563-572.

Gold, R. (2021). Status Report on Electrification Policy: Where to Next? Current Sustainable / Renewable Energy Reports, 8(2), 114–122. https://doi.org/10.1007/s40518-021-00180-w

Golic, K., Kosoric, V., Kosic, T., Vuckovic, S., & Kujundzic, K. (2023). A Platform of Critical Barriers to Socially Sustainable Residential Buildings: Experts' Perspective. SUSTAINABILITY, 15(9). https://doi.org/10.3390/su15097485

Golizadeh, H., Hosseini, M., Edwards, D., Abrishami, S., Taghavi, N., & Banihashemi, S. (2019). Barriers to adoption of RPAs on construction projects: A task-technology fit perspective. CONSTRUCTION INNOVATION-ENGLAND, 19(2), 149–169. https://doi.org/10.1108/CI-09-2018-0074

Gombošová, V., Krajčík, M., & Šikula, O. (2022). Feasibility of Using Energy Performance Contracting for the Retrofit of Apartment Buildings in Slovakia. Slovak Journal of Civil Engineering, 30(3), 33–42. https://doi.org/10.2478/sjce-2022-0019

Gonzalez Quiros, A., MacAllister, D. J., MacDonald, A., Palumbo-Roe, B., Bearcock, J., Ó Dochartaigh, B., Callaghan, E., Kearsey, T., Walker-Verkuil, K., & Monaghan, A. (2024). De-risking green energy from mine waters by developing a robust hydrogeological conceptual model of the UK Geoenergy Observatory in Glasgow. Hydrogeology Journal, 32(5), 1307–1329. https://doi.org/10.1007/s10040-024-02778-y

González-Torres, M., Pérez-Lombard, L., Coronel, J., Maestre, I., & Yan, D. (2022). A review on buildings energy information: Trends, end-uses, fuels and drivers. ENERGY REPORTS, 8, 626–637. https://doi.org/10.1016/j.egyr.2021.11.280

Gordon, J. A., Balta-Ozkan, N., Haq, A. U., & Nabavi, S. A. (2024). Heterogeneous preferences for living in a hydrogen home: An advanced multigroup analysis. Sustainable Energy & Fuels, 8(12), 2601–2648. https://doi.org/10.1039/D4SE00392F

Göswein, V., Carvalho, S., Cerqueira, C., & Lorena, A. (2022). Circular material passports for buildings – Providing a robust methodology for promoting circular buildings. IOP Conference Series. Earth and Environmental Science, 1122(1), 012049. https://doi.org/10.1088/1755-1315/1122/1/012049

Gounder, S., Hasan, A., Shrestha, A., & Elmualim, A. (2023). Barriers to the use of sustainable materials in Australian building projects. ENGINEERING CONSTRUCTION AND ARCHITECTURAL MANAGEMENT, 30(1), 189–209. https://doi.org/10.1108/ECAM-10-2020-0854

Grady, G. C. (2012). Land Use Incentives And Enforcement In Government "Green" Requirements. *The Practical Real Estate Lawyer*, *28*(2), 43.

Graham, R., & Dutton, J. (2021). Obsolescence as an Opportunity: The Role of Adaptive Reuse in Calgary's Office Market. The School of Public Policy Publications (SPPP), 14, n/a. https://doi.org/10.11575/sppp.v14i.71326

Gram-Hanssen, K., Jensen, J. O., & Friis, F. (2018). Local strategies to promote energy retrofitting of single-family houses. Energy Efficiency, 11(8), 1955. https://doi.org/10.1007/s12053-018-9653-5

Groh, A., Hunter Kuhlwein, & Bienert, S. (2022). Does Retrofitting Pay Off? An Analysis of German Multifamily Building Data. Journal of Sustainable Real Estate, 14(1), 95–112. https://doi.org/10.1080/19498276.2022.2135188

Gross, R., & Hanna, R. (2019). Path dependency in provision of domestic heating. NATURE ENERGY, 4(5), 358–364. https://doi.org/10.1038/s41560-019-0383-5

Gu, N., & London, K. (2010). Understanding and facilitating BIM adoption in the AEC industry. Automation in Construction, 19(8), 988–999. https://doi.org/10.1016/j.autcon.2010.09.002

Guerra, B., & Leite, F. (2021). Circular economy in the construction industry: An overview of United States stakeholders' awareness, major challenges, and enablers. RESOURCES CONSERVATION AND RECYCLING, 170. https://doi.org/10.1016/j.resconrec.2021.105617

Gundlach, J., & Stein, E. B. (2020). HARMONIZING STATES' ENERGY UTILITY REGULATION FRAMEWORKS AND CLIMATE LAWS: A CASE STUDY OF NEW YORK. *Energy Law Journal*, *41*(2), 211–260.

Guo, M., Fu, Y., PhD, Liu, M., PhD, & O'Neill, Z., PhD, PE. (2024). Investigations on the Influence of Model Accuracy in Deep Reinforcement Learning Control for HVAC Applications. *ASHRAE Transactions*, *130*, 570–579.

Hafez, H., El-Mahdy, D., & Marsh, A. (2023). Barriers and enablers for scaled-up adoption of compressed earth blocks in Egypt. BUILDING RESEARCH AND INFORMATION, 51(7), 783–797. https://doi.org/10.1080/09613218.2023.2237133

Haidar, A., Guimón, J., & Martínez, J. (2022). Mainstreaming graphene in electrochemical energy storage devices: A Delphi-based adaptive priority-setting. ENERGY FOR SUSTAINABLE DEVELOPMENT, 71, 279–290. https://doi.org/10.1016/j.esd.2022.10.004

Hailemariam, E., Hailemariam, L., Amede, E., & Nuramo, D. (2023). Identification of barriers, benefits and opportunities of using bamboo materials for structural purposes. ENGINEERING CONSTRUCTION AND ARCHITECTURAL MANAGEMENT, 30(7), 2716–2738. https://doi.org/10.1108/ECAM-11-2021-0996

Hajare, A. (2019). Life Cycle Cost Analysis of an Energy Efficient Residential Unit. ProQuest Dissertations and Theses. https://ezproxy.bu.edu/login?qurl=https://www.proquest.com/docview/2827703667?accountid=9676&bdid=81940&_bd=Hlp5yVtbbgz7jLkxdz%2FF%2FDWU9Is%3D

Hajare, A., & Elwakil, E. (2020). Integration of life cycle cost analysis and energy simulation for building energy-efficient strategies assessment. SUSTAINABLE CITIES AND SOCIETY, 61. https://doi.org/10.1016/j.scs.2020.102293

Hale, S. E., Roque, A. J., Okkenhaug, G., Sørmo, E., Lenoir, T., Carlsson, C., Kupryianchyk, D., Flyhammar, P., & Žlender, B. (2021). The Reuse of Excavated Soils from Construction and Demolition Projects: Limitations and Possibilities. Sustainability, 13(11), 6083. https://doi.org/10.3390/su13116083

Hamed Olfat, Rismanchi, B., Shojaei, D., & Rajabifard, A. (2019). Utilizing a Building Information Modelling Environment to Communicate the Legal Ownership of Internet of Things-Generated Data in Multi-Owned Buildings. Electronics, 8(11), 1258. https://doi.org/10.3390/electronics8111258

Hanus, N., Newkirk, A., & Stratton, H. (2023). Organizational and psychological measures for data center energy efficiency: Barriers and mitigation strategies. Energy Efficiency, 16(1), 1. https://doi.org/10.1007/s12053-022-10078-1

Hanus, N., Wong-Parodi, G., Small, M., & Grossmann, I. (2018). The role of psychology and social influences in energy efficiency adoption. ENERGY EFFICIENCY, 11(2), 371–391. https://doi.org/10.1007/s12053-017-9568-6

Hariri, M. E., & Youssef, T. (2022). Impact of Electricity Pricing Mechanisms on Grid-Interactive Buildings. The Institute of Electrical and Electronics Engineers, Inc. (IEEE) Conference Proceedings. https://doi.org/10.1109/TESC53336.2022.9917257

Harris, N., Shealy, T., Parrish, K., & Granderson, J. (2019). Cognitive barriers during monitoring-based commissioning of buildings. SUSTAINABLE CITIES AND SOCIETY, 46. https://doi.org/10.1016/j.scs.2018.12.017

Hartmann, K., & Palm, J. (2023). The role of thermal energy communities in Germany's heating transition. FRONTIERS IN SUSTAINABLE CITIES, 4. https://doi.org/10.3389/frsc.2022.1027148

Haselsteiner, E., Rizvanolli, B. V., Paola Villoria Sáez, & Kontovourkis, O. (2021). Drivers and Barriers Leading to a Successful Paradigm Shift toward Regenerative Neighborhoods. Sustainability, 13(9), 5179. https://doi.org/10.3390/su13095179

Hassan, A., Negash, Y., & Hanum, F. (2024). An assessment of barriers to digital transformation in circular Construction: An application of stakeholder theory. AIN SHAMS ENGINEERING JOURNAL, 15(7). https://doi.org/10.1016/j.asej.2024.102787

Hassanain, M., Alamoudi, A., Al-Hammad, A., & Abdallah, A. (2020). Barriers to the implementation of POE practices in the Saudi Arabian building industry. ARCHITECTURAL ENGINEERING AND DESIGN MANAGEMENT, 16(2), 150–165. https://doi.org/10.1080/17452007.2019.1706440

He, W., King, M., Luo, X., Dooner, M., Li, D., & Wang, J. (2021). Technologies and economics of electric energy storages in power systems: Review and perspective. ADVANCES IN APPLIED ENERGY, 4. https://doi.org/10.1016/j.adapen.2021.100060

Heffernan, E., Pan, W., Liang, X., & de Wilde, P. (2015). Zero carbon homes: Perceptions from the UK construction industry. Energy Policy, 79, 23. https://doi.org/10.1016/j.enpol.2015.01.005

Heider, E., & Brockman, C. (2009). BUILDINGS ALIVE! ESTABLISHING THE COST OF LIVING BUILDINGS STRIVING FOR NET ZERO PERFORMANCE. JOURNAL OF GREEN BUILDING, 4(3), 61–71. https://doi.org/10.3992/jgb.4.3.61

Helal, J., Trabucco, D., Ruggiero, D., Miglietta, P., & Perrucci, G. (2024). Embodied Carbon Premium for Cantilevers. Buildings, 14(4), 871. https://doi.org/10.3390/buildings14040871

Hemström, K., Mahapatra, K., & Gustavsson, L. (2017). Architects' perception of the innovativeness of the Swedish construction industry. Construction Innovation, 17(2), 244–260. https://doi.org/10.1108/CI-06-2015-0038

Herrador, M. (2024). Assessment of the first-ever circular economy framework of Cambodia: Barriers, international opportunities and recommendations. JOURNAL OF CLEANER PRODUCTION, 438. https://doi.org/10.1016/j.jclepro.2024.140778

Herrando, M., & Ramos, A. (2022). Photovoltaic-Thermal (PV-T) Systems for Combined Cooling, Heating and Power in Buildings: A Review. Energies, 15(9), 3021. https://doi.org/10.3390/en15093021

Hirokawa, K. H. (2009). At home with nature: Early reflections on green building laws and the transformation of the built environment. *Environmental Law*, *39*(3), 507–576.

Hodorog, A., Petri, I., Rezgui, Y., & Hippolyte, J. (2021). Building information modelling knowledge harvesting for energy efficiency in the Construction industry. CLEAN TECHNOLOGIES AND ENVIRONMENTAL POLICY, 23(4), 1215–1231. https://doi.org/10.1007/s10098-020-02000-z

Hohmann, B. (2019). Strategies for a sustainable energy transition: The case of the housing sector in Graz, Austria (WOS:000562136800179). 323. https://doi.org/10.1088/1755-1315/323/1/012180

Höjer, M., & Mjörnell, K. (2018). Measures and Steps for More Efficient Use of Buildings. Sustainability, 10(6), 1949. https://doi.org/10.3390/su10061949

Holmes, K., Zeitler, E., Kerxhalli-Kleinfield, M., & DeBoer, R. (2021). Scaling Deep Decarbonization Technologies. EARTHS FUTURE, 9(11). https://doi.org/10.1029/2021EF002399

Honold, A., Lützkendorf, T., & Lohse, R. (2020). Business Models in the context of carbon mitigation: New Questions and Approaches illustrated by the Example of Energy Performance Contracting in Germany. IOP Conference Series. Earth and Environmental Science, 588(2). https://doi.org/10.1088/1755-1315/588/2/022062

Horup, L., Reymann, M., Rørbech, J. T., Ryberg, M., & Birkved, M. (2019). Partially dynamic life cycle assessment of windows indicates potential thermal over-optimization. IOP Conference Series. Earth and Environmental Science, 323(1). https://doi.org/10.1088/1755-1315/323/1/012152

Hosseini, M., Memari, S., Martek, I., Kocaturk, T., Bararzadeh, M., & Arashpour, M. (2024). Dismantling linear lock-ins in the Australian AEC industry: A pathway to a circular economy. SUSTAINABLE DEVELOPMENT. https://doi.org/10.1002/sd.3082

Hou, J., Liu, Y., Wu, Y., Zhou, N., & Feng, W. (2016). Comparative study of commercial building energy-efficiency retrofit policies in four pilot cities in China. ENERGY POLICY, 88, 204–215. https://doi.org/10.1016/j.enpol.2015.10.016

Hoxha, V., & Lecaj, V. (2024). Regulatory barriers to implementing sustainable buildings in Kosovo. PROPERTY MANAGEMENT, 42(2), 293–309. https://doi.org/10.1108/PM-01-2022-0008

Hoyland, S., Kjestveit, K., & Skotnes, R. (2023). Exploring the complexity of hydrogen perception and acceptance among key stakeholders in Norway. INTERNATIONAL JOURNAL OF HYDROGEN ENERGY, 48(21), 7896–7908. https://doi.org/10.1016/j.ijhydene.2022.11.144

Hu, M., & Qiu, Y. (2019). A comparison of building energy codes and policies in the USA, Germany, and China: Progress toward the net-zero building goal in three countries. CLEAN TECHNOLOGIES AND ENVIRONMENTAL POLICY, 21(2), 291–305. https://doi.org/10.1007/s10098-018-1636-x

Hu, X., Wang, C., & Elshkaki, A. (2024). Material-energy Nexus: A systematic literature review. RENEWABLE & SUSTAINABLE ENERGY REVIEWS, 192. https://doi.org/10.1016/j.rser.2023.114217

Huang, P., Zhang, X., Copertaro, B., Saini, P. K., Yan, D., Wu, Y., & Chen, X. (2020). A Technical Review of Modeling Techniques for Urban Solar Mobility: Solar to Buildings, Vehicles, and Storage (S2BVS). Sustainability, 12(17), 7035. https://doi.org/10.3390/su12177035

Huang, Y. (2020). Factors Affecting Residents' Adoption of Energy-efficient Lighting in Rural Areas of China. IOP Conference Series. Earth and Environmental Science, 495(1). https://doi.org/10.1088/1755-1315/495/1/012016

Hui, F., Ulya, P., Wilson, S., Meyliawati, A., & Aye, L. (2020). Green Buildings in Makassar, Indonesia. In GREEN BUILDING IN DEVELOPING COUNTRIES: POLICY, STRATEGY AND TECHNOLOGY (WOS:000489143800008; pp. 109–127). https://doi.org/10.1007/978-3-030-24650-1_610.1007/978-3-030-24650-1

Huong, Q., Lou, E., & Le Hoai, N. (2021). Enhancing BIM Diffusion through Pilot Projects in Vietnam. ENGINEERING JOURNAL-THAILAND, 25(7), 167–176. https://doi.org/10.4186/ej.2021.25.7.167

Hurlimann, A., Browne, G., Warren-Myers, G., & Francis, V. (2018). Barriers to climate change adaptation in the Australian construction industry—Impetus for regulatory reform. BUILDING AND ENVIRONMENT, 137, 235–245. https://doi.org/10.1016/j.buildenv.2018.04.015

Husgafvel, R., & Sakaguchi, D. (2022). Circular Economy Development in the Construction Sector in Japan. WORLD, 3(1), 1–26. https://doi.org/10.3390/world3010001

Hussain, B., Ali Naqvi, S. A., & Anwar, S. (2023). Analyzing the Impact of Critical Barriers on the Stakeholder's Adoption Behavior toward Green Building Technologies in Pakistan. International Journal of Management Research and Emerging Science, 13(1). https://doi.org/10.56536/ijmres.v13i1.371

Hwang, B., Shan, M., & Lye, J. (2018). Adoption of sustainable construction for small contractors: Major barriers and best solutions. CLEAN TECHNOLOGIES AND ENVIRONMENTAL POLICY, 20(10), 2223–2237. https://doi.org/10.1007/s10098-018-1598-z

IEEE, Alizadehsalehi, S., Hadavi, A., & Huang, J. (2019). BIM/MR-Lean Construction Project Delivery Management System (WOS:000589702100010). 2019 IEEE TECHNOLOGY & ENGINEERING MANAGEMENT CONFERENCE (TEMSCON). https://doi.org/10.1109/temscon.2019.8813574

IEEE, Lukasser, G., Estaji, A., Sauter, T., Wilker, S., Kobelrausch, M., Leibold, J., & Sengl, D. (2022). Integrating Photovoltaics and Sun Blinds for Smart Shading Systems (WOS:000946662000154). 990–993. https://doi.org/10.1109/ISIE51582.2022.9831737

Ielegems, E., Herssens, J., Nuyls, E., & Vanrie, J. (2019). DRIVERS AND BARRIERS FOR UNIVERSAL DESIGNING: A SURVEY ON ARCHITECTS' PERCEPTIONS. *JOURNAL OF ARCHITECTURAL AND PLANNING RESEARCH*, *36*(3), 181–197.

Ilgın, H. E., Saviharju, A., Karjalainen, M., & Hirvilammi, T. (2024). Life Cycle Assessment of an Office Building in Finland Using a Custom Assessment Tool. Buildings, 14(7), 1944. https://doi.org/10.3390/buildings14071944

Ince, R. N. (2015). Urban Retrofit: Pressures, Policy and People in Domestic Retrofit at the City Level. PQDT - Global. https://ezproxy.bu.edu/login?qurl=https://www.proquest.com/docview/2566020949?accountid=9676&bdid=77468&_bd=rOV8txljL8PUqMk%2BixHhZzNoHs8%3D

IOP Publishing, Kamaruddin, T., Hamid, R., & Abd Ghani, S. (2020). Social aspect implementation in sustainable construction (WOS:000594080900036). 849. https://doi.org/10.1088/1757-899X/849/1/012036

IOP, Jamaludin, S., Hamid, S., & Noor, S. (2020). Assessing the Challenges of Integration Affordable and Sustainable Housing from Economic Perspectives (WOS:000614604800089). 498. https://doi.org/10.1088/1755-1315/498/1/012089

IOP, Ng, L., Tan, L., & Seow, T. (2018). Constraints to 3R construction waste reduction among contractors in Penang (WOS:000454977100103). 140. https://doi.org/10.1088/1755-1315/140/1/012103

IOP, Polychroni, E., & Androutsopoulos, A. (2020). Innovative financial schemes for buildings' energy renovation (WOS:000538681000055). 410. https://doi.org/10.1088/1755-1315/410/1/012055

IOP, Suhaida, M., Tan, K., & Leong, Y. (2013). Green buildings in Malaysia towards greener environment: Challenges for policy makers (WOS:000322788100121). 16. https://doi.org/10.1088/1755-1315/16/1/012121

Iqbal, M., Ma, J., Ahmad, N., Hussain, K., Usmani, M., & Ahmad, M. (2021). Sustainable construction through energy management practices in developing economies: An analysis of barriers in the construction sector. ENVIRONMENTAL SCIENCE AND POLLUTION RESEARCH, 28(26), 34793–34823. https://doi.org/10.1007/s11356-021-12917-7

Iqbal, M., Ma, J., Ahmad, N., Ullah, Z., & Ahmed, R. (2021). Uptake and Adoption of Sustainable Energy Technologies: Prioritizing Strategies to Overcome Barriers in the Construction Industry by Using an Integrated AHP-TOPSIS Approach. ADVANCED SUSTAINABLE SYSTEMS, 5(7). https://doi.org/10.1002/adsu.202100026

Italian Architectural Heritage and Photovoltaic Systems. Matching Style with Sustainability. (2021). Sustainability, 13(4), 2108. https://doi.org/10.3390/su13042108

Izaola, B., Akizu-Gardoki, O., & Oregi, X. (2022). Life Cycle Analysis Challenges through Building Rating Schemes within the European Framework. Sustainability, 14(9), 5009. https://doi.org/10.3390/su14095009

Jack, M., Mirfin, A., & Anderson, B. (2021). The role of highly energy-efficient dwellings in enabling 100% renewable electricity. ENERGY POLICY, 158. https://doi.org/10.1016/j.enpol.2021.112565

Jahangir, M., Mokhtari, R., Salmanpour, F., & Yousefi, H. (2024). Urban energy planning towards achieving an economically and environmentally optimized energy flow by 2050 based on different scenarios (a case study). ENVIRONMENT DEVELOPMENT AND SUSTAINABILITY. https://doi.org/10.1007/s10668-024-04754-8

Jahed, N., Aktaş, Y. D., Rickaby, P., & Ayşe Güliz Bilgin Altınöz. (2020). Policy Framework for Energy Retrofitting of Built Heritage: A Critical Comparison of UK and Turkey. Atmosphere, 11(6), 674. https://doi.org/10.3390/atmos11060674

Jalilzadehazhari, E., & Kurkinen, E.-L. (2022). Drivers and barrios in using augmented reality in renovation projects—Literature review. E3S Web of Conferences, 362, n/a. https://doi.org/10.1051/e3sconf/202236207002

Janhunen, E., & Junnila, S. (2022). The contribution of smart buildings to low-carbon built environment. IOP Conference Series. Earth and Environmental Science, 1101(2), 022010. https://doi.org/10.1088/1755-1315/1101/2/022010

Jansen, B. W., Jin-Ah Duijghuisen, Gerard van Bortel, & Gruis, V. (2023). Comparing Circular Kitchens: A Study of the Dutch Housing Sector. Buildings, 13(7), 1698. https://doi.org/10.3390/buildings13071698

Jayalath, A., Navaratnam, S., Gunawardena, T., Mendis, P., & Aye, L. (2021). Airborne and impact sound performance of modern lightweight timber buildings in the Australian construction industry. CASE STUDIES IN CONSTRUCTION MATERIALS, 15. https://doi.org/10.1016/j.cscm.2021.e00632

Jayawardana, J., Sandanayake, M., Jayasinghe, S., Kulatunga, A., & Zhang, G. (2024). Key barriers and mitigation strategies towards sustainable prefabricated construction—A case of developing economies. ENGINEERING CONSTRUCTION AND ARCHITECTURAL MANAGEMENT. https://doi.org/10.1108/ECAM-09-2023-0978

Jayaweera, R., Nop, S., Karagianni, C., Waibel, M., & Schwede, D. (2022). Sustainable building arenas: Constructing a governance framework for a sustainability transition in Cambodia's urban built environment. IOP Conference Series. Earth and Environmental Science, 1078(1), 012084. https://doi.org/10.1088/1755-1315/1078/1/012084

Jiang, H., Yao, R., Han, S., Du, C., Yu, W., Chen, S., Li, B., Yu, H., Li, N., Peng, J., & Li, B. (2020). How do urban residents use energy for winter heating at home? A large-scale survey in the hot summer and cold winter climate zone in the Yangtze River region. ENERGY AND BUILDINGS, 223. https://doi.org/10.1016/j.enbuild.2020.110131

Jing, K., Qing, C., & Yee, H. (2023). Readiness of Malaysian on Sustainable Development in Solar Energy Application. INTERNATIONAL JOURNAL OF SUSTAINABLE CONSTRUCTION ENGINEERING AND TECHNOLOGY, 14(1), 189–201. https://doi.org/10.30880/ijscet.2023.14.01.017

John Kaiser Calautit, & Hughes, B. R. (2016). Sustainable Buildings: Opportunities, challenges, aims and vision. Sustainable Buildings, 1, n/a. https://doi.org/10.1051/sbuild/2016001

Juan, Y.-K., Lai, W.-Y., & Shih, S.-G. (2017). Building information modeling acceptance and readiness assessment in Taiwanese architectural firms. Journal of Civil Engineering and Management, 23(3), 356–367. https://doi.org/10.3846/13923730.2015.1128480

Kachirayil, F., Weinand, J., Scheller, F., & McKenna, R. (2022). Reviewing local and integrated energy system models: Insights into flexibility and robustness challenges. APPLIED ENERGY, 324. https://doi.org/10.1016/j.apenergy.2022.119666

Kaewunruen, S., Teuffel, P., Cavdar, A., Valta, O., Tambovceva, T., & Bajare, D. (2024). Comparisons of stakeholders' influences, inter-relationships, and obstacles for circular economy implementation on existing building sectors. SCIENTIFIC REPORTS, 14(1). https://doi.org/10.1038/s41598-024-61863-0

Kamranfar, S., Azimi, Y., Gheibi, M., Fathollahi-Fard, A., & Hajiaghaei-Keshteli, M. (2022). Analyzing Green Construction Development Barriers by a Hybrid Decision-Making Method Based on DEMATEL and the ANP. BUILDINGS, 12(10). https://doi.org/10.3390/buildings12101641

Kamranfar, S., Damirchi, F., Pourvaziri, M., Xalikovich, P., Mahmoudkelayeh, S., Moezzi, R., & Vadiee, A. (2023). A Partial Least Squares Structural Equation Modelling Analysis of the Primary Barriers to Sustainable Construction in Iran. SUSTAINABILITY, 15(18). https://doi.org/10.3390/su151813762

Kanai, J., Fabio, V., Miras, M., & Gastiarena, L. (2024). Making Green Heritage Schools Work: Nature-Based Solutions and Historical Preservation When Infrastructure Fails. SUSTAINABILITY, 16(16). https://doi.org/10.3390/su16166981

Kang, J., Hao, B., Li, Y., Lin, H., & Xue, Z. (2022). The Application and Development of LVDC Buildings in China. ENERGIES, 15(19). https://doi.org/10.3390/en15197045

Kanyilmaz, A., Birhane, M., Fishwick, R., & del Castillo, C. (2023). Reuse of Steel in the Construction Industry: Challenges and Opportunities. INTERNATIONAL JOURNAL OF STEEL STRUCTURES, 23(5), 1399–1416. https://doi.org/10.1007/s13296-023-00778-4

Karlsson, I., Rootzén, J., Toktarova, A., Odenberger, M., Johnsson, F., & Göransson, L. (2020). Roadmap for Decarbonization of the Building and Construction Industry—A Supply Chain Analysis Including Primary Production of Steel and Cement. Energies, 13(16), 4136. https://doi.org/10.3390/en13164136

Kashkash, S., Czoboly, O., & Orban, Z. (2023). Effect of Moisture Condition and the Composition of Aggregate from Demolition Waste on Strength and Workability Properties of Recycled Concrete. Buildings, 13(7), 1870. https://doi.org/10.3390/buildings13071870

Katerusha, D. (2021). Barriers to the use of recycled concrete from the perspective of executing companies and possible solution approaches—Case study Germany and Switzerland. Resources Policy, 73, 1. https://doi.org/10.1016/j.resourpol.2021.102212

Kazemi, M., & Kazemi, A. (2022). Financial barriers to residential buildings' energy efficiency in Iran. ENERGY EFFICIENCY, 15(5). https://doi.org/10.1007/s12053-022-10039-8

Kazemi, M., & Udall, J. (2023). Behavioral barriers to the use of renewable and energy-efficient technologies in residential buildings in Iran. ENERGY EFFICIENCY, 16(7). https://doi.org/10.1007/s12053-023-10162-0

Ke, Z., Zhang, H., Jia, X., Yan, J., Lv, X., Yu, H., Gao, N., Zeng, W., Liu, Y., & Wong, N. (2024). Research on energy efficiency and decarbonization pathway of nearly zero energy buildings based on system dynamic simulation. DEVELOPMENTS IN THE BUILT ENVIRONMENT, 17. https://doi.org/10.1016/j.dibe.2023.100310

Kelly, N., Edkins, A. J., Smyth, H., & Konstantinou, E. (2013). Reinventing the role of the project manager in mobilising knowledge in construction. International Journal of Managing Projects in Business, 6(4), 654. https://doi.org/10.1108/IJMPB-12-2011-0080

Kesidou, S., & Sorrell, S. (2018). Low-carbon innovation in non-domestic buildings: The importance of supply chain integration. ENERGY RESEARCH & SOCIAL SCIENCE, 45, 195–213. https://doi.org/10.1016/j.erss.2018.07.018

Keskin, F. S. (2021). Integration of Lifecycle Assessment into Structural Intervention Scenarios for Assessing Environmental Impacts of Vulnerable Buildings. PQDT - UK & Ireland. https://ezproxy.bu.edu/login?qurl=https://www.proquest.com/docview/2796568324?accountid=9676&bdid=81940&_bd=h4LLxRmQC5TWd0JqyLPt%2BteQU2w%3D

Khaba, S., & Bhar, C. (2017). Modeling the key barriers to lean construction using interpretive structural modeling. Journal of Modelling in Management, 12(4), 652–670. https://doi.org/10.1108/JM2-07-2015-0052

Khafiso, T., Aigbavboa, C., & Adekunle, S. (2024). Barriers to the adoption of energy management systems in residential buildings. FACILITIES, 42(15/16), 107–125. https://doi.org/10.1108/F-12-2023-0113

Khalid, Y., Ngwaka, U., Papworth, J., Ling-Chin, J., & Smallbone, A. (2023). Evaluation of decarbonisation options for heritage church buildings. JOURNAL OF BUILDING ENGINEERING, 77. https://doi.org/10.1016/j.jobe.2023.107462

Khalil, A. (2018). Developing a Strategy for the Implementation of Sustainable Construction Practices in Libya. PQDT - UK & Ireland. https://ezproxy.bu.edu/login?qurl=https://www.proquest.com/docview/2342402186?accountid=9676&bdid=81940&_bd=O5RA6wHVqlTO1HMdfRZqqTQmr%2FQ%3D

Khalil, A., Rathnasinghe, A., & Kulatunga, U. (2021). Challenges for the Implementation of Sustainable Construction Practices in Libya. CONSTRUCTION ECONOMICS AND BUILDING, 21(3), 243–261. https://doi.org/10.5130/AJCEB.v21i3.7647

Khan, M., Dani, A. A., Lim, J. B. P., & Roy, K. (2024). Appraising the Feasibility of 3D Printing Construction in New Zealand Housing. Buildings, 14(4), 1084. https://doi.org/10.3390/buildings14041084

Khozema Ahmed Ali, Ahmad, M. I., & Yusup, Y. (2020). Issues, Impacts, and Mitigations of Carbon Dioxide Emissions in the Building Sector. Sustainability, 12(18), 7427. https://doi.org/10.3390/su12187427

Kim, K. P. (2020). Cloud-BIM Enabled Cyber-Physical Data and Service Platforms for Building Component Reuse. Sustainability, 12(24), 10329. https://doi.org/10.3390/su122410329

Klöckner, C. A., & Nayum, A. (2017). Psychological and structural facilitators and barriers to energy upgrades of the privately owned building stock. *Energy*, *140*, 1005.

Knoeri, C., Schwarz, M., & Nakhle, C. (2019). Energy transition and technical energy regulations in the building sector. IOP Conference Series. Earth and Environmental Science, 323(1). https://doi.org/10.1088/1755-1315/323/1/012181

Kordana-Obuch, S., Starzec, M., Wojtoń, M., & Słyś, D. (2023). Greywater as a Future Sustainable Energy and Water Source: Bibliometric Mapping of Current Knowledge and Strategies. Energies, 16(2), 934. https://doi.org/10.3390/en16020934

Koster, G., van Sark, W., & Ricker, B. (2024). Solar potential for social benefit: Maps to sustainably address energy poverty utilizing open spatial data in data poor settings. ENERGY FOR SUSTAINABLE DEVELOPMENT, 80. https://doi.org/10.1016/j.esd.2024.101453

Koutra, S., Terés-Zubiaga, J., Bouillard, P., & Becue, V. (2023). “Decarbonizing Europe” A critical review on positive energy districts approaches. SUSTAINABLE CITIES AND SOCIETY, 89. https://doi.org/10.1016/j.scs.2022.104356

Kozminska, U. (2019). Circular design: Reused materials and the future reuse of building elements in architecture. Process, challenges and case studies. IOP Conference Series. Earth and Environmental Science, 225(1). https://doi.org/10.1088/1755-1315/225/1/012033

KPMG’s Singapore Budget 2022 proposal highlights ESG, global tax and enterprise support measures to build lasting companies on a fragile planet. (2022). LiveNews.Co.Nz. https://ezproxy.bu.edu/login?qurl=https://www.proquest.com/docview/2644775334?accountid=9676&bdid=77468&_bd=8QYkGez%2Boy1KL4k7hWSrUKBZuEI%3D

Krueger, K., Stoker, A., & Gaustad, G. (2019). “Alternative” materials in the green building and construction sector; Examples, barriers, and environmental analysis. Smart and Sustainable Built Environment, 8(4), 270–291. https://doi.org/10.1108/SASBE-09-2018-0045

Kudirat Olabisi Ayinla, & Adamu, Z. (2018). Bridging the digital divide gap in BIM technology adoption. Engineering, Construction and Architectural Management, 25(10), 1398–1416. https://doi.org/10.1108/ECAM-05-2017-0091

Kuppusamy, S., Chew, H., Mari, T., & Chai, C. (2019). Implementation of green building materials in construction industry in Johor Bahru, Malaysia (WOS:000493910800006). 268. https://doi.org/10.1088/1755-1315/268/1/012006

Kusuma, B., Soemardi, B. W., Pribadi, K. S., & Yuliar, S. (2019). Indonesian contractor technological learning mechanism and its considerations. IOP Conference Series. Materials Science and Engineering, 650(1). https://doi.org/10.1088/1757-899X/650/1/012001

Lam, P. T., & YU, J. S. (2016). Developing and managing photovoltaic facilities based on third-party ownership business models in buildings. Facilities, 34(13/14), 855–872. https://doi.org/10.1108/F-04-2015-0019

Langston, C., & Zhang, W. (2021). DfMA: Towards an Integrated Strategy for a More Productive and Sustainable Construction Industry in Australia. SUSTAINABILITY, 13(16). https://doi.org/10.3390/su13169219

Larrea-Sáez, L., Muñoz, E., Cuevas, C., & Casas-Ledón, Y. (2024). Optimizing insulation and heating systems for social housing in Chile: Insights for sustainable energy policies. ENERGY, 290. https://doi.org/10.1016/j.energy.2023.130024

Lassandro, P., Devitofrancesco, A., Bellazzi, A., Cascardi, A., De Aloysio, G., Laghi, L., & Malvezzi, R. (2024). Facing the Constraints to the Deep Energy Renovation Process of Residential Built Stock in European Markets. SUSTAINABILITY, 16(1). https://doi.org/10.3390/su16010294

Latha, H., Patil, S., & Kini, P. G. (2023). Influence of architectural space layout and building perimeter on the energy performance of buildings: A systematic literature review. International Journal of Energy and Environmental Engineering, 14(3), 431–474. https://doi.org/10.1007/s40095-022-00522-4

Lavikka, R., Chauhan, K., Peltokorpi, A., & Seppänen, O. (2021). Value creation and capture in systemic innovation implementation: Case of mechanical, electrical and plumbing prefabrication in the Finnish construction sector. CONSTRUCTION INNOVATION-ENGLAND, 21(4), 837–856. https://doi.org/10.1108/CI-05-2020-0070

Le Dréau, J., Lopes, R., ’Connell, S., Finn, D., Hu, M., Queiroz, H., Alexander, D., Satchwell, A., Österreicher, D., Polly, B., Arteconi, A., Pereira, F., Hall, M., Kirant-Mitic, T., Cai, H., Johra, H., Kazmi, H., Li, R., Liu, A., … Saeed, M. (2023). Developing energy flexibility in clusters of buildings: A critical analysis of barriers from planning to operation. ENERGY AND BUILDINGS, 300. https://doi.org/10.1016/j.enbuild.2023.113608

Le, A., Navodana Rodrigo, Niluka Domingo, & Senaratne, S. (2023). Policy Mapping for Net-Zero-Carbon Buildings: Insights from Leading Countries. Buildings, 13(11), 2766. https://doi.org/10.3390/buildings13112766

Le, D., Salomone, R., Nguyen, Q., Versele, A., & Piccardo, C. (2024). Status and barriers to circular bio-based building material adoption in developed economies: The case of Flanders, Belgium. JOURNAL OF ENVIRONMENTAL MANAGEMENT, 367. https://doi.org/10.1016/j.jenvman.2024.121965

Leal, W., Fedoruk, M., Zahvoyska, L., & Avila, L. (2021). Identifying and Comparing Obstacles and Incentives for the Implementation of Energy Saving Projects in Eastern and Western European Countries: An Exploratory Study. SUSTAINABILITY, 13(9). https://doi.org/10.3390/su13094944

Leerbeck, K., Bacher, P., Grønborg, R., Tveit, A., Corradi, O., & Madsen, H. (2020). Control of heat pumps with CO2 emission intensity forecasts. *arXiv.Org*, n/a.

Leite, F., Cho, Y., Behzadan, A., Lee, S., Choe, S., Fang, Y., Akhavian, R., & Hwang, S. (2016). Visualization, Information Modeling, and Simulation: Grand Challenges in the Construction Industry. JOURNAL OF COMPUTING IN CIVIL ENGINEERING, 30(6). https://doi.org/10.1061/(ASCE)CP.1943-5487.0000604

Leoto, R., & Lizarralde, G. (2019). Challenges in evaluating strategies for reducing a building’s environmental impact through Integrated Design. BUILDING AND ENVIRONMENT, 155, 34–46. https://doi.org/10.1016/j.buildenv.2019.03.041

Lesnyak, E., Belkot, T., Hurka, J., Hörding, J. P., Kuhlmann, L., Paulau, P., Schnabel, M., Schönfeldt, P., & Middelberg, J. (2023). Applied Digital Twin Concepts Contributing to Heat Transition in Building, Campus, Neighborhood, and Urban Scale. Big Data and Cognitive Computing, 7(3), 145. https://doi.org/10.3390/bdcc7030145

Less, B., Casquero-Modrego, N., & Walker, I. (2022). Home Energy Upgrades as a Pathway to Home Decarbonization in the US: A Literature Review. ENERGIES, 15(15). https://doi.org/10.3390/en15155590

Levesque, A., Pietzcker, R., Baumstark, L., & Luderer, G. (2021). Deep decarbonisation of buildings energy services through demand and supply transformations in a 1.5°C scenario. ENVIRONMENTAL RESEARCH LETTERS, 16(5). https://doi.org/10.1088/1748-9326/abdf07

Li, F. G., Trutnevyte, E., & Strachan, N. (2015). A review of socio-technical energy transition (STET) models. Technological Forecasting and Social Change, 100, 290. https://doi.org/10.1016/j.techfore.2015.07.017

Li, J., & Colombier, M. (2009). Managing carbon emissions in China through building energy efficiency. JOURNAL OF ENVIRONMENTAL MANAGEMENT, 90(8), 2436–2447. https://doi.org/10.1016/j.jenvman.2008.12.015

Li, J., & Gou, Z. (2024). Addressing the development gap in net-zero energy buildings: A comparative study of China, India, and the United States. ENERGY FOR SUSTAINABLE DEVELOPMENT, 79. https://doi.org/10.1016/j.esd.2024.101418

Li, J., Zhang, J., Liu, C., & Luo, X. (2023). What hinders the implementation of carbon emission reduction in the construction industry: Evidence from integrated multi-criteria decision-making in China. ENGINEERING CONSTRUCTION AND ARCHITECTURAL MANAGEMENT. https://doi.org/10.1108/ECAM-05-2023-0511

Li, R., Satchwell, A., Finn, D., Christensen, T., Kummert, M., Le Dréau, J., Lopes, R., Madsen, H., Salom, J., Henze, G., & Wittchen, K. (2022). Ten questions concerning energy flexibility in buildings. BUILDING AND ENVIRONMENT, 223. https://doi.org/10.1016/j.buildenv.2022.109461

Li, S., Zhou, C., & Wang, S. (2019). Does modernization affect carbon dioxide emissions? A panel data analysis. SCIENCE OF THE TOTAL ENVIRONMENT, 663, 426–435. https://doi.org/10.1016/j.scitotenv.2019.01.373

Li, T., Shapiro, M., Heidarinejad, M., & Stephens, B. (2024). Ten questions concerning building electrification. BUILDING AND ENVIRONMENT, 261. https://doi.org/10.1016/j.buildenv.2024.111653

Li, Y., Liu, Z., & Li, C. (2022). OVERVIEW OF GOVERNMENT STRATEGIES ON GREEN BUILDING IN SINGAPORE. *JOURNAL OF GREEN BUILDING*, *17*(4), 219–241.

Li, Y., Zhu, N., & Qin, B. (2019). Major Barriers to the New Residential Building Energy-Efficiency Promotion in China: Frontlines' Perceptions. ENERGIES, 12(6). https://doi.org/10.3390/en12061073

Liao, H., Ren, R., & Lu, L. (2023). Existing Building Renovation: A Review of Barriers to Economic and Environmental Benefits. International Journal of Environmental Research and Public Health, 20(5), 4058. https://doi.org/10.3390/ijerph20054058

Likita, A., Jelodar, M., Vishnupriya, V., Rotimi, J., & Vilasini, N. (2022). Lean and BIM Implementation Barriers in New Zealand Construction Practice. BUILDINGS, 12(10). https://doi.org/10.3390/buildings12101645

Lim, B., Liu, A., & Oo, B. (2019). Awareness and Practices of Sustainable Construction in Australia: Consultant Quantity Surveyors' Perception (WOS:000524614000060). 2124. https://doi.org/10.1063/1.5117120

Liu, B., Yang, H., & Bai, Y. (2024). Barriers to the Development of The Industrialized House Building in China. The Institute of Electrical and Electronics Engineers, Inc. (IEEE) Conference Proceedings. https://doi.org/10.1109/ICIT58233.2024.10540956

Liu, C., Sharples, S., & Mohammadpourkarbasi, H. (2023). A Review of Building Energy Retrofit Measures, Passive Design Strategies and Building Regulation for the Low Carbon Development of Existing Dwellings in the Hot Summer–Cold Winter Region of China. Energies, 16(10), 4115. https://doi.org/10.3390/en16104115

Liu, D. (2013). CSR and Sustainability in the Building and Construction Industry: Green Lease Case Study. ProQuest Dissertations and Theses. https://ezproxy.bu.edu/login?qurl=https://www.proquest.com/docview/1530436508?accountid=9676&bdid=81940&_bd=ltquWy0rcVBYuDFrUzu5CJyOpbc%3D

Liu, G., Li, X., Tan, Y., & Zhang, G. (2020). Building green retrofit in China: Policies, barriers and recommendations. ENERGY POLICY, 139. https://doi.org/10.1016/j.enpol.2020.111356

Liu, J., Wu, P., Jiang, Y., & Wang, X. (2021). Explore potential barriers of applying circular economy in construction and demolition waste recycling. JOURNAL OF CLEANER PRODUCTION, 326. https://doi.org/10.1016/j.jclepro.2021.129400

Liu, Z., Yu, C., Qian, Q., Huang, R., You, K., Visscher, H., & Zhang, G. (2023). Incentive initiatives on energy-efficient renovation of existing buildings towards carbon-neutral blueprints in China: Advancements, challenges and prospects. ENERGY AND BUILDINGS, 296. https://doi.org/10.1016/j.enbuild.2023.113343

Lotz, M. T., Barkhausen, R., Herbst, A., Pfaff, M., Durand, A., & Rehfeldt, M. (2022). Potentials and Prerequisites on the Way to a Circular Economy: A Value Chain Perspective on Batteries and Buildings. Sustainability, 14(2), 956. https://doi.org/10.3390/su14020956

Lucchi, E. (2022). Integration between photovoltaic systems and cultural heritage: A socio-technical comparison of international policies, design criteria, applications, and innovation developments. Energy Policy, 171, 113303.

Lucchi, E. (2022). Integration between photovoltaic systems and cultural heritage: A socio-technical comparison of international policies, design criteria, applications, and innovation developments. ENERGY POLICY, 171. https://doi.org/10.1016/j.enpol.2022.113303

Lucchi, E., Baiani, S., & Altamura, P. (2023). Design criteria for the integration of active solar technologies in the historic built environment: Taxonomy of international recommendations. ENERGY AND BUILDINGS, 278. https://doi.org/10.1016/j.enbuild.2022.112651

Ludwig, G. (2019). The Role of Law in Transformative Environmental PoliciesA Case Study of "Timber in Buildings Construction in Germany." SUSTAINABILITY, 11(3). https://doi.org/10.3390/su11030842

Lund, H., Jakob Zinck Thellufsen, Aggerholm, S., Wittchen, K. B., Nielsen, S., Brian Vad Mathiesen, & Möller, B. (2014). Heat Saving Strategies in Sustainable Smart Energy Systems. International Journal of Sustainable Energy Planning and Management, 4, 3. https://doi.org/10.5278/ijsepm.2014.4.2

Luo, T., Xue, X., Wang, Y., Xue, W., & Tan, Y. (2021). A systematic overview of prefabricated construction policies in China. JOURNAL OF CLEANER PRODUCTION, 280. https://doi.org/10.1016/j.jclepro.2020.124371

Lygnerud, K., Klugman, S., Fransson, N., & Nilsson, J. (2022). Risk assessment of industrial excess heat collaborations e Empirical data from new and ongoing installations. ENERGY, 255. https://doi.org/10.1016/j.energy.2022.124452

Lynch, H. (2014). Passivhaus in the UK : the challenges of an emerging market: A case study of innovation using mixed methods research. PQDT - UK & Ireland. https://ezproxy.bu.edu/login?qurl=https://www.proquest.com/docview/2351328288?accountid=9676&bdid=81940&_bd=%2BR%2FIrL3UOd9gJHMw%2BiMZCYbnPKg%3D

Lynch, N. (2022). Unbuilding the city: Deconstruction and the circular economy in Vancouver. ENVIRONMENT AND PLANNING A-ECONOMY AND SPACE, 54(8), 1586–1603. https://doi.org/10.1177/0308518X221116891

Ma, M., Tam, V. W. Y., Le, K. N., & Li, W. (2020). Challenges in current construction and demolition waste recycling: A China study. *Waste management (New York, N.Y.)*, *118*, 610–625. https://doi.org/10.1016/j.wasman.2020.09.030

Ma, S. (2024). SUSTAINABLE HOUSING DEVELOPMENT IN CHINA: DOES FINANCIAL INSTITUTIONS OVERCOME THE RISKS AND CHALLENGES TO SUSTAINABLE HOUSING? TECHNOLOGICAL AND ECONOMIC DEVELOPMENT OF ECONOMY, 30(3), 627–645. https://doi.org/10.3846/tede.2024.20581

Mac Uidhir, T., Rogan, F., Collins, M., Curtis, J., & Gallachóir, B. (2020). Improving energy savings from a residential retrofit policy: A new model to inform better retrofit decisions. ENERGY AND BUILDINGS, 209. https://doi.org/10.1016/j.enbuild.2019.109656

MacLaren, V., & Ikiz, E. (2022). Meeting urban GHG reduction goals with waste diversion: Multi-residential buildings. Buildings & Cities, 3(1), 1042. https://doi.org/10.5334/bc.277

Madadizadeh, A., Siddiqui, K., & Aliabadi, A. A. (2024). Review: The Economics Landscape for Building Decarbonization. Sustainability, 16(14), 6214. https://doi.org/10.3390/su16146214

Mahmod, A., Mohd, S., Masirin, M., Tajudin, S., Bakar, I., Zainorabidin, A., Kifli, A., & Hua, L. (2016). Construction of Buildings on Peat: Case Studies and Lessons Learned (WOS:000387756300053). 47. https://doi.org/10.1051/matecconf/20164703013

Mahmoodi, M., Rasheed, E., & Le, A. (2024). Systematic Review on the Barriers and Challenges of Organisations in Delivering New Net Zero Emissions Buildings. Buildings, 14(6), 1829. https://doi.org/10.3390/buildings14061829

Mahpour, A. (2018). Prioritizing barriers to adopt circular economy in construction and demolition waste management. RESOURCES CONSERVATION AND RECYCLING, 134, 216–227. https://doi.org/10.1016/j.resconrec.2018.01.026

Maia, I., Kranzl, L., & Müller, A. (2021). New step-by-step retrofitting model for delivering optimum timing. APPLIED ENERGY, 290. https://doi.org/10.1016/j.apenergy.2021.116714

Malla, V., Prasad, K., & Delhi, V. (2024). Analysing inhibitors to BIM implementation: A social network theoretical perspective. CONSTRUCTION INNOVATION-ENGLAND. https://doi.org/10.1108/CI-06-2023-0128

Mamter, S., Abdul-Aziz, A. R., & Mamat, M. (2017). Stimulating a Sustainable Construction through Holistic BIM Adoption: The Root Causes of Recurring Low BIM Adoption in Malaysia. IOP Conference Series. Materials Science and Engineering, 216(1). https://doi.org/10.1088/1757-899X/216/1/012056

Mangan, S. D. (2023). A Performance-Based Decision Support Workflow for Retrofitting Residential Buildings. Sustainability, 15(3), 2567. https://doi.org/10.3390/su15032567

Maniak-Huesser, M., Tellnes, L., & Escamilla, E. (2021). Mind the Gap: A Policy Gap Analysis of Programmes Promoting Timber Construction in Nordic Countries. SUSTAINABILITY, 13(21). https://doi.org/10.3390/su132111876

Mao, C., Shen, Q., Pan, W., & Ye, K. (2015). Major Barriers to Off-Site Construction: The Developer's Perspective in China. JOURNAL OF MANAGEMENT IN ENGINEERING, 31(3). https://doi.org/10.1061/(ASCE)ME.1943-5479.0000246

Wuni, I. Y. (2022). Mapping the barriers to circular economy adoption in the construction industry: A systematic review, Pareto analysis, and mitigation strategy map. Building and Environment, 223, 109453.

Maqsood, T., Wong, P. S., Malik Khalfan, & Yang, R. J. (2021). Extended Producer Responsibility in the Australian Construction Industry. Sustainability, 13(2), 620. https://doi.org/10.3390/su13020620

Maqsoom, A., Zulqarnain, M., Irfan, M., Ullah, F., Alqahtani, F., & Khan, K. (2023). Drivers of, and Barriers to, the Adoption of Mixed Reality in the Construction Industry of Developing Countries. BUILDINGS, 13(4). https://doi.org/10.3390/buildings13040872

Marco Lamperti Tornaghi, Loli, A., & Negro, P. (2018). Balanced Evaluation of Structural and Environmental Performances in Building Design. Buildings, 8(4), 52. https://doi.org/10.3390/buildings8040052

Marhani, M., & Muksain, M. (2018). GBI assessment checklist: Level of awareness of the contractors in the Malaysian construction industry (WOS:000445893500024). 117. https://doi.org/10.1088/1755-1315/117/1/012024

Markel Arbulu, Perez-Bezos, S., Figueroa-Lopez, A., & Oregi, X. (2024). Opportunities and Barriers of Calibrating Residential Building Performance Simulation Models Using Monitored and Survey-Based Occupant Behavioural Data: A Case Study in Northern Spain. Buildings, 14(7), 1911. https://doi.org/10.3390/buildings14071911

Marszal-Pomianowska, A., Motoasca, E., Pothof, I., Felsmann, C., Heiselberg, P., Cadenbach, A., Leusbrock, I., ’Donovan, K., Petersen, S., & Schaffer, M. (2024). Strengths, weaknesses, opportunities and threats of demand response in district heating and cooling systems. From passive customers to valuable assets. SMART ENERGY, 14. https://doi.org/10.1016/j.segy.2024.100135

Martin, H., Chebrolu, D., Chadee, A., & Brooks, T. (2024). Too good to waste: Examining circular economy opportunities, barriers, and indicators for sustainable construction and demolition waste management. SUSTAINABLE PRODUCTION AND CONSUMPTION, 48, 460–480. https://doi.org/10.1016/j.spc.2024.05.026

Martin, J., & Samels, J. E. (2012). The Sustainable University; Green Goals and New Challenges for Higher Education Leaders. The Sustainable University. https://ezproxy.bu.edu/login?qurl=https://www.proquest.com/docview/2131353040?accountid=9676&bdid=77468&_bd=PsjKy9eEHsdeH48%2Fx10JH5bLPkg%3D

Martín-Consuegra, F., Ludueña, C. A., De Frutos, F., Frutos, B., Alonso, C., & Oteiza, I. (2024). Expectations and Outcomes when Quantifying Energy Improvements Achieved by Building Envelope Retrofitting. Sustainability, 16(8), 3214. https://doi.org/10.3390/su16083214

Martinez, E., Reid, C., & Tommelein, I. (2019). Lean construction for affordable housing: A case study in Latin America. CONSTRUCTION INNOVATION-ENGLAND, 19(4), 570–593. https://doi.org/10.1108/CI-02-2019-0015

Martínez, S., Harmsen, R., Menkveld, M., Faaij, A., & Kramer, G. (2022). Municipalities as key actors in the heat transition to decarbonise buildings: Experiences from local planning and implementation in a learning context. ENERGY POLICY, 169. https://doi.org/10.1016/j.enpol.2022.113169

Märzinger, T., & Österreicher, D. (2020). Extending the Application of the Smart Readiness Indicator—A Methodology for the Quantitative Assessment of the Load Shifting Potential of Smart Districts. Energies, 13(13), 3507. https://doi.org/10.3390/en13133507

Mata, É., Wanemark, J., Cheng, S. H., E Ó Broin, Hennlock, M., & Sandvall, A. (2021). Systematic map of determinants of buildings’ energy demand and CO2 emissions shows need for decoupling. Environmental Research Letters, 16(5). https://doi.org/10.1088/1748-9326/abe5d7

Matheus Koengkan, Fuinhas, J. A., Auza, A., & Ursavaş, U. (2023). The Impact of Energy Efficiency Regulations on Energy Poverty in Residential Dwellings in the Lisbon Metropolitan Area: An Empirical Investigation. Sustainability, 15(5), 4214. https://doi.org/10.3390/su15054214

Matheus Koengkan, Fuinhas, J. A., Radulescu, M., Kazemzadeh, E., Nooshin Karimi Alavijeh, Santiago, R., & Teixeira, M. (2023). Assessing the Role of Financial Incentives in Promoting Eco-Friendly Houses in the Lisbon Metropolitan Area—Portugal. Energies, 16(4), 1839. https://doi.org/10.3390/en16041839

Mathis, C. (2014). Bringing ASHRAE Home. *ASHRAE Journal*, *56*(5), 27–28.

Mccarty, J., Scott, A., & Rysanek, A. (2021). Determining the retrofit viability of Vancouver’s single-detached homes: An expert elicitation. BUILDINGS & CITIES, 2(1), 354–377. https://doi.org/10.5334/bc.85

Meacham, B. J. (2010). Accommodating innovation in building regulation: Lessons and challenges. *Building Research and Information*, *38*(6), 686.

Meckling, J., Sterner, T., & Wagner, G. (2017). Policy sequencing toward decarbonization. NATURE ENERGY, 2(12), 918–922. https://doi.org/10.1038/s41560-017-0025-8

Mehaffy, M. W. (2024). UN-Sustainable Urbanism: The Challenge of “Lock-In.” Sustainability, 16(17), 7301. https://doi.org/10.3390/su16177301
Mehibel, M. (2018). Sustainable housing design and development in relation to the city of Jijel, Algeria. PQDT - UK & Ireland. https://ezproxy.bu.edu/login?qurl=https://www.proquest.com/docview/2341280385?accountid=9676&bdid=81940&_bd=skr5ywg1yxrs5bjRgVEOO%2FwnI5c%3D
Mehta, K., & Zörner, W. (2023). Cracking the code: Mapping residential building energy performance in rural Central Asia through building typologies. SN Applied Sciences, 5(12), 349. https://doi.org/10.1007/s42452-023-05607-1
Meinardi, F., Bruni, F., & Brovelli, S. (2017). Luminescent solar concentrators for building-integrated photovoltaics. Nature Reviews. Materials, 2(12), 17072. https://doi.org/10.1038/natrevmats.2017.72
Mellross, M., & Fraser, B. (2012). DEVELOPING MUNICIPAL POLICY AND PROGRAMS TO ACCELERATE MARKET TRANSFORMATION IN THE BUILDING SECTOR. JOURNAL OF GREEN BUILDING, 7(4), 46–61. https://doi.org/10.3992/jgb.7.4.46
Menegazzo, D., Lombardo, G., Bobbo, S., De Carli, M., & Fedele, L. (2022). State of the Art, Perspective and Obstacles of Ground-Source Heat Pump Technology in the European Building Sector: A Review. Energies, 15(7), 2685. https://doi.org/10.3390/en15072685
Mengistu, D., Ashene, D., & Beyene, M. (2024). Micro and Small Enterprises’ Development in the Ethiopian Construction Industry: The Challenges and Improvement Regulatory Framework. JOURNAL OF CONSTRUCTION IN DEVELOPING COUNTRIES, 29(1), 67–85. https://doi.org/10.21315/jcdc-12-22-0214
Mengistu, D., Ashene, D., & Halabo, H. (2023). Technology and innovation development in Ethiopian construction industry: The challenges and improvement mechanisms. JOURNAL OF ENGINEERING DESIGN AND TECHNOLOGY. https://doi.org/10.1108/JEDT-10-2022-0528
Merrett, N. (2024a). Bringing down barriers to “old build” efficiency. *H & V News*, 26–27.
Merrett, N. (2024b). Energy efficiency is not just a heat pump issue. *H & V News*, 18–20.
Michelsen, C., Rosenschon, S., & Schulz, C. (2015). Small might be beautiful, but bigger performs better: Scale economies in “green” refurbishments of apartment housing. Energy Economics, 50, 240–250. https://doi.org/10.1016/j.eneco.2015.05.012
Mišík, M., Oravcová, V., & Vicenová, R. (2024). Energy efficiency of buildings in Central and Eastern Europe: Room for improvement. Energy Efficiency, 17(4), 32. https://doi.org/10.1007/s12053-024-10215-y
Moghaddasi, H., Culp, C., & Vanegas, J. (2021). Net Zero Energy Communities: Integrated Power System, Building and Transport Sectors. Energies, 14(21), 7065. https://doi.org/10.3390/en14217065
Mohamed, M., Ibrahim, A., Bashir, F., Chammam, A., Gnaba, H., Kadhim, S., & Khalilpoor, N. (2023). An assessment of the barriers to the adoption of green building technologies in Saudi Arabia. INTERNATIONAL JOURNAL OF LOW-CARBON TECHNOLOGIES, 18, 872–880. https://doi.org/10.1093/ijlct/ctad064
Mohammad, W., Abdullah, M., Ismail, S., & Takim, R. (2018). Building Information Modeling (BIM) adoption challenges for contractor’s organisations in Malaysia (WOS:000481577600148). 2016. https://doi.org/10.1063/1.5055550
Mok, K., Shen, G., & Yang, R. (2018). Stakeholder complexity in large scale green building projects: A holistic analysis towards a better understanding. ENGINEERING CONSTRUCTION AND ARCHITECTURAL MANAGEMENT, 25(11), 1454–1474. https://doi.org/10.1108/ECAM-09-2016-0205
Molnár, G., Cabeza, L., Chatterjee, S., & Ürge-Vorsatz, D. (2024). Modelling the building-related photovoltaic power production potential in the light of the EU’s Solar Rooftop Initiative. APPLIED ENERGY, 360. https://doi.org/10.1016/j.apenergy.2024.122708
Momade, M., Durdyev, S., Tam, N., Shahid, S., Mbachu, J., & Momade, Y. (2022). Factors influencing adoption of construction technologies in Vietnam’s residential construction projects. INTERNATIONAL JOURNAL OF BUILDING PATHOLOGY AND ADAPTATION. https://doi.org/10.1108/IJBPA-03-2022-0048
Momennia Rankohi, S. (2022). Investigating the Concept of Integration in Construction Projects: Cases of Integrated Project Delivery and Design for Manufacturing and Assembly. ProQuest Dissertations and Theses. https://ezproxy.bu.edu/login?qurl=https://www.proquest.com/docview/3102062916?accountid=9676&bdid=81940&_bd=uYZ0vbT5Jhpd3%2BwPtBMP3s2kCs8%3D

Moncaster, A., Malmqvist, T., Forman, T., Pomponi, F., & Anderson, J. (2022). Embodied carbon of concrete in buildings, Part 2: Are the messages accurate? Buildings & Cities, 3(1), 334. https://doi.org/10.5334/bc.199

Montero, O., Brischoux, P., Callegari, S., Fraga, C., Rüetschi, M., Vionnet, E., Calame, N., Rognon, F., Patel, M., & Hollmuller, P. (2022). Large Air-to-Water Heat Pumps for Fuel-Boiler Substitution in Non-Retrofitted Multi-Family Buildings—Energy Performance, CO2 Savings, and Lessons Learned in Actual Conditions of Use. Energies, 15(14), 5033. https://doi.org/10.3390/en15145033

Moradi, S., & Sormunen, P. (2023). Implementing Lean Construction: A Literature Study of Barriers, Enablers, and Implications. BUILDINGS, 13(2). https://doi.org/10.3390/buildings13020556

Morgan, G. T., Coleman, S., Robinson, J. B., Touchie, M. F., Poland, B., Jakubiec, A., Lach, N., & Cao, Y. (2022). Wellbeing as an emergent property of social practice. Buildings & Cities, 3(1), 756. https://doi.org/10.5334/bc.262

Morris, J., Allinson, D., Harrison, J., & Lomas, K. J. (2016). Benchmarking and tracking domestic gas and electricity consumption at the local authority level. Energy Efficiency, 9(3), 723. https://doi.org/10.1007/s12053-015-9393-8

Morrison, G. M. (2021). A Rapid Review on Community Connected Microgrids. Sustainability, 13(12), 6753. https://doi.org/10.3390/su13126753

Moshood, T., Rotimi, J., & Shahzad, W. (2024). Enhancing sustainability considerations in construction industry projects. ENVIRONMENT DEVELOPMENT AND SUSTAINABILITY. https://doi.org/10.1007/s10668-024-04946-2

Moyo, T., & Chigara, B. (2023). Barriers to lean construction implementation in Zimbabwe. JOURNAL OF ENGINEERING DESIGN AND TECHNOLOGY, 21(3), 733–757. https://doi.org/10.1108/JEDT-01-2021-0044

Muhammad Sami Ur Rehman, Shafiq, M. T., Ullah, F., & Khaled Galal Ahmed. (2023). A critical appraisal of traditional methods of construction progress monitoring. Built Environment Project and Asset Management, 13(6), 830–845. https://doi.org/10.1108/BEPAM-02-2023-0040

Mulligan, T., Mollaoglu-Korkmaz, S., Cotner, R., & Goldsberry, A. (2014). PUBLIC POLICY AND IMPACTS ON ADOPTION OF SUSTAINABLE BUILT ENVIRONMENTS: LEARNING FROM THE CONSTUCTION INDUSTRY PLAYMAKERS. JOURNAL OF GREEN BUILDING, 9(2), 182–202. https://doi.org/10.3992/1943-4618-9.2.182

Mustaffa, N., & Kudus, S. (2022). Challenges and way forward towards best practices of energy efficient building in Malaysia. ENERGY, 259. https://doi.org/10.1016/j.energy.2022.124839

Naber, E., Volk, R., & Schultmann, F. (2017). From the building level energy performance assessment to the national level: How are uncertainties handled in building stock models (WOS:000404873600150). 180, 1443–1452. https://doi.org/10.1016/j.proeng.2017.04.307

Nadel, S. (2019). Electrification in the Transportation, Buildings, and Industrial Sectors: A Review of Opportunities, Barriers, and Policies. Current Sustainable / Renewable Energy Reports, 6(4), 158–168. https://doi.org/10.1007/s40518-019-00138-z

Nair, G., Fransson, Å., & Olofsson, T. (2021). Perspectives of building professionals on the use of LCA tools in Swedish climate declaration (WOS:000668011100075). 246. https://doi.org/10.1051/e3sconf/202124613004

Narula, K., Ploiner, C., Getzinger, G., & Patel, M. (2022). Impact of energy efficiency and decarbonisation policies for buildings: A comparative assessment of Austria and Switzerland. ENERGY AND BUILDINGS, 268. https://doi.org/10.1016/j.enbuild.2022.112175

Nastasi, B. (2016). Renewable energy generation and integration in Sustainable Buildings – a focus on eco-fuels. Sustainable Buildings, 1, n/a. https://doi.org/10.1051/sbuild/2016003

Ndekugri, I., Ankrah, N. A., & Adaku, E. (2022). The design coordination role at the pre-construction stage of construction projects. Building Research and Information, 50(4), 452–466. https://doi.org/10.1080/09613218.2021.1971061

Negash, Y., Hassan, A., Tseng, M., Wu, K., & Ali, M. (2021). Sustainable construction and demolition waste management in Somaliland: Regulatory barriers lead to technical and environmental barriers. JOURNAL OF CLEANER PRODUCTION, 297. https://doi.org/10.1016/j.jclepro.2021.126717

Nerval, C. (2024). Industry needs policy certainty to win the sustainability battle. *Property Week*, 17–17.

Newton, S., Shirazi, A., & Christensen, P. (2023). Defining and demonstrating a smart technology configuration to improve energy performance and occupant comfort in existing buildings: A conceptual framework. International Journal of Building Pathology and Adaptation, 41(1), 182–200. https://doi.org/10.1108/IJBPA-04-2021-0046

NezamiFar, E. (2020). Building Occupant Environmental Behaviour (Boeb) Model for Leed-Certified Buildings. PQDT - UK & Ireland. https://ezproxy.bu.edu/login?qurl=https://www.proquest.com/docview/2497466992?accountid=9676&bdid=81940&_bd=oBkq%2Bqp99AaXRoIPO9LbVLmQAHA%3D

Nguyen, H., Skitmore, M., Gray, M., Zhang, X., & Olanipekun, A. (2017). Will green building development take off? An exploratory study of barriers to green building in Vietnam. RESOURCES CONSERVATION AND RECYCLING, 127, 8–20. https://doi.org/10.1016/j.resconrec.2017.08.012

Nguyen, T., & Nguyen, D. (2021). Barriers in BIM Adoption and the Legal Considerations in Vietnam. INTERNATIONAL JOURNAL OF SUSTAINABLE CONSTRUCTION ENGINEERING AND TECHNOLOGY, 12(1), 283–295. https://doi.org/10.30880/ijscet.2021.12.01.027

Nidam, Y., Irani, A., Bemis, J., & Reinhart, C. (2023). Census-based urban building energy modeling to evaluate the effectiveness of retrofit programs. ENVIRONMENT AND PLANNING B-URBAN ANALYTICS AND CITY SCIENCE, 50(9), 2394–2406. https://doi.org/10.1177/23998083231154576

Nikolaidou, E., Walker, I., Coley, D., Allen, S., Fosas, D., & Roberts, M. (2022). Towards Active Buildings: Stakeholder Perceptions of the Next Generation of Buildings. Energies, 15(15), 5706. https://doi.org/10.3390/en15155706

Nilchian, S., Sardroud, J. M., Darabpour, M., & Tafreshi, S. T. (2022). Features and Conditions of Building Information Modeling Contracts. Buildings, 12(11), 1839. https://doi.org/10.3390/buildings12111839

Nilimaa, J. (2023). Smart materials and technologies for sustainable concrete construction. DEVELOPMENTS IN THE BUILT ENVIRONMENT, 15. https://doi.org/10.1016/j.dibe.2023.100177

Nordt, A., Raven, R., Malekpour, S., & Sharp, D. (2024). Decarbonising industry supply chains: Incumbent-oriented transition intermediation for industry energy transition. ENVIRONMENTAL INNOVATION AND SOCIETAL TRANSITIONS, 51. https://doi.org/10.1016/j.eist.2024.100843

Novikova, A., Csoknyai, T., & Szalay, Z. (2018). Low carbon scenarios for higher thermal comfort in the residential building sector of South Eastern Europe. Energy Efficiency, 11(4), 845–875. https://doi.org/10.1007/s12053-017-9604-6

Nur Soleha Abdul Rahim, Balqish Abdul Rahman, Fazdliel Aswad Ibrahim, Ishak, N., & Ayob, A. (2023). The Contractors' Perception on the Development of Green Building Projects in Penang. IOP Conference Series. Earth and Environmental Science, 1238(1), 012020. https://doi.org/10.1088/1755-1315/1238/1/012020

Nußholz, J. L. K., Nygaard Rasmussen, F., & Milios, L. (2019). Circular building materials: Carbon saving potential and the role of business model innovation and public policy. Resources, Conservation and Recycling, 141, 308–316. https://doi.org/10.1016/j.resconrec.2018.10.036

Nweye, K., Sankaranarayanan, S., & Nagy, Z. (2023). MERLIN: Multi-agent offline and transfer learning for occupant-centric operation of grid-interactive communities. APPLIED ENERGY, 346. https://doi.org/10.1016/j.apenergy.2023.121323

Nyawa, S., Gnekpe, C., & Tchuente, D. (2023). Transparent machine learning models for predicting decisions to undertake energy retrofits in residential buildings. ANNALS OF OPERATIONS RESEARCH. https://doi.org/10.1007/s10479-023-05217-5

Obergassel, W., Xia-Bauer, C., & Thomas, S. (2023). Strengthening global climate governance and international cooperation for energy-efficient buildings. ENERGY EFFICIENCY, 16(8). https://doi.org/10.1007/s12053-023-10177-7

Obrist, M., Kannan, R., McKenna, R., Schmidt, T., & Kober, T. (2023). High-temperature heat pumps in climate pathways for selected industry sectors in Switzerland. ENERGY POLICY, 173. https://doi.org/10.1016/j.enpol.2022.113383

Oduro, S., Pittri, H., Simons, B., Baah, B., Anteh, E., & Oduro, J. (2024). Awareness of net zero energy buildings among construction professionals in the Ghanaian construction industry. BUILT ENVIRONMENT PROJECT AND ASSET MANAGEMENT. https://doi.org/10.1108/BEPAM-01-2024-0001

Oguntona, O. A., & Aigbavboa, C. O. (2019). Barriers Militating Against the Adoption of Biomimicry as a Sustainable Construction Practice. MATEC Web of Conferences, 266, n/a. https://doi.org/10.1051/matecconf/201926603010

Ohene, E., Chan, A. P. C., Darko, A., & Nani, G. (2023). Navigating toward net zero by 2050: Drivers, barriers, and strategies for net zero carbon buildings in an emerging market. Building and Environment, 242, 110472. https://doi.org/10.1016/j.buildenv.2023.110472

Ojelabi, R. A., Mohammed, T. A., & Oladiran, O. J. (2024). Awareness and Implementation Challenges of the Green Retrofitting in Building Enclosure in the Nigerian Construction Industry. IOP Conference Series. Earth and Environmental Science, 1342(1), 012023. https://doi.org/10.1088/1755-1315/1342/1/012023

Oke, A., Aigbavboa, C., & Khangale, T. (2018). Effect of Skills Shortage on Sustainable Construction (WOS:000465822600029). 600, 303–309. https://doi.org/10.1007/978-3-319-60450-3_29

Oke, A., Aliu, J., Ehiosun, L., Kineber, A., & Stephen, S. (2024). Adoption of distributed ledger technology for construction projects: A study of the challenges in a developing country. JOURNAL OF ENGINEERING DESIGN AND TECHNOLOGY. https://doi.org/10.1108/JEDT-11-2023-0474

Oke, D., Aghimien, D., Aigbavboa, C., & Musenga, C. (2019). Drivers of Sustainable Construction Practices in the Zambian Construction Industry (WOS:000471031703095). 158, 3246–3252. https://doi.org/10.1016/j.egypro.2019.01.995

Olanrewaju, O., Enegbuma, W., Donn, M., & Chileshe, N. (2022). Building information modelling and green building certification systems: A systematic literature review and gap spotting. SUSTAINABLE CITIES AND SOCIETY, 81. https://doi.org/10.1016/j.scs.2022.103865

Olatunji Ayodeji Aiyetan & Das Dillip. (2018). System Dynamics Approach to Mitigating Skilled Labour Shortages in the Construction Industry: A South Africa Context. Construction Economics and Building, 18(4), 45. https://doi.org/10.5130/AJCEB.v18i4.6041

Olatunji, O. A. (2014). Views on building information modelling, procurement and contract management. Proceedings of the Institution of Civil Engineers. Management, Procurement and Law, 167(3), 117–126. https://doi.org/10.1680/mpal.13.00011

Olatz Nicolas, & Molina-Costa, P. (2021). Demand Aggregation as a Strategy for Untapping Buildings' Energy Renovation Potential: Diagnosis and Prioritization Methodology and Case Study from the Basque Country. Sustainability, 13(24), 13881. https://doi.org/10.3390/su132413881

Olawumi, T., & Chan, D. (2020). Concomitant impediments to the implementation of smart sustainable practices in the built environment. SUSTAINABLE PRODUCTION AND CONSUMPTION, 21, 239–251. https://doi.org/10.1016/j.spc.2019.09.001

Olawumi, T., Chan, D., Wong, J., & Chan, A. (2018). Barriers to the integration of BIM and sustainability practices in construction projects: A Delphi survey of international experts. JOURNAL OF BUILDING ENGINEERING, 20, 60–71. https://doi.org/10.1016/j.jobe.2018.06.017

Olivadese, R., Remøy, H., Berizzi, C., & Hobma, F. (2017). Reuse into housing: Italian and Dutch regulatory effects. Property Management, 35(2), 165. https://doi.org/10.1108/PM-10-2015-0054

Oliveira, R., Almeida, R. M., Figueiredo, A., & Romeu Vicente. (2021). A Case Study on a Stochastic-Based Optimisation Approach towards the Integration of Photovoltaic Panels in Multi-Residential Social Housing. Energies, 14(22), 7615. https://doi.org/10.3390/en14227615

Oliveira, R., Lopes, J., & Abreu, M. (2021). Sustainability Perspective to Support Decision Making in Structural Retrofitting of Buildings: A Case Study. SYSTEMS, 9(4). https://doi.org/10.3390/systems9040078

Olsthoorn, M., Schleich, J., & Faure, C. (2019). Exploring the diffusion of low-energy houses: An empirical study in the European Union. ENERGY POLICY, 129, 1382–1393. https://doi.org/10.1016/j.enpol.2019.03.043

Oluleye, B. I., Chan, D. W., & Olawumi, T. O. (2022). Barriers to circular economy adoption and concomitant implementation strategies in building construction and demolition waste management: A PRISMA and interpretive structural modeling approach. *Habitat International*, *126*, 102615.

Oluleye, B., Chan, D., Olawumi, T., & Saka, A. (2023). Assessment of symmetries and asymmetries on barriers to circular economy adoption in the construction industry towards zero waste: A survey of international experts. BUILDING AND ENVIRONMENT, 228. https://doi.org/10.1016/j.buildenv.2022.109885

Omar, M., Ayob, A., Zakaria, M., Rahim, N., Mokhtar, H., Rani, H., & Rahman, F. (2023). Perspective of Construction Building Professionals on Low-Carbon Materials in Malaysia. JOURNAL OF CONSTRUCTION IN DEVELOPING COUNTRIES, 28(2), 139–162. https://doi.org/10.21315/jcdc-04-22-0081

Omopariola, E., Olanrewaju, O., Albert, I., Oke, A., & Ibiyemi, S. (2024). Sustainable construction in the Nigerian construction industry: Unsustainable practices, barriers and strategies. JOURNAL OF ENGINEERING DESIGN AND TECHNOLOGY, 22(4), 1158–1184. https://doi.org/10.1108/JEDT-11-2021-0639

Omotayo, T., Ross, J., Oyetunji, A., & Udeaja, C. (2024). Systems Thinking Interplay Between Project Complexities, Stakeholder Engagement, and Social Dynamics Roles in Influencing Construction Project Outcomes. SAGE OPEN, 14(2). https://doi.org/10.1177/21582440241255872

Onecha, B., Dotor, A., & Marmolejo-Duarte, C. (2021). Beyond Cultural and Historic Values, Sustainability as a New Kind of Value for Historic Buildings. Sustainability, 13(15), 8248. https://doi.org/10.3390/su13158248

Onososen, A., & Musonda, I. (2022). Barriers to BIM-Based Life Cycle Sustainability Assessment for Buildings: An Interpretive Structural Modelling Approach. BUILDINGS, 12(3). https://doi.org/10.3390/buildings12030324

Onososen, A., Musonda, I., Onatayo, D., Tjebane, M., Saka, A., & Fagbenro, R. (2023). Impediments to Construction Site Digitalisation Using Unmanned Aerial Vehicles (UAVs). DRONES, 7(1). https://doi.org/10.3390/drones7010045

Onubogu, N. O., Kok-Keong Chong, & Ming-Hui, T. (2021). Review of Active and Passive Daylighting Technologies for Sustainable Building. International Journal of Photoenergy, 2021. https://doi.org/10.1155/2021/8802691

Optimising energy efficiency for the comfort of the nZEB experimental building: A new approach. (2023). AIP Conference Proceedings, 2928(1). https://doi.org/10.1063/5.0170895

Osei-Tutu, S., Ayarkwa, J., Dickson Osei-Asibey, Nani, G., & Afful, A. E. (2023). Barriers impeding circular economy (CE) uptake in the construction industry. Smart and Sustainable Built Environment, 12(4), 892–918. https://doi.org/10.1108/SASBE-03-2022-0049

Oshiro, K., Masui, T., & Kainuma, M. (2018). Transformation of Japan’s energy system to attain net-zero emission by 2050. CARBON MANAGEMENT, 9(5), 493–501. https://doi.org/10.1080/17583004.2017.1396842

Osuizugbo, I., Nnodu, C., Onokwai, A., & Olojo, A. (2024). Perceived Factors Inhibiting Environmental Impact Assessment Practice in Construction Industry. PRACTICE PERIODICAL ON STRUCTURAL DESIGN AND CONSTRUCTION, 29(1). https://doi.org/10.1061/PPSCFX.SCENG-1257

Ouf, M., Osman, M., Bitzilos, M., & Gunay, B. (2024). Can you lower the thermostat? Perceptions of demand response programs in a sample from Quebec. ENERGY AND BUILDINGS, 306. https://doi.org/10.1016/j.enbuild.2024.113933

Oyefusi, O., Arowoiya, V., & Chan, M. (2024). Hybrid MCDM approach for analyzing barriers and formulating strategies for the adoption of modular construction in developing countries. ENGINEERING CONSTRUCTION AND ARCHITECTURAL MANAGEMENT. https://doi.org/10.1108/ECAM-01-2024-0082

Oyewobi, L., & Jimoh, R. (2022). Barriers to Adoption of Sustainable Procurement in the Nigerian Public Construction Sector. SUSTAINABILITY, 14(22). https://doi.org/10.3390/su142214832

Pacheco, M., & Lamberts, R. (2013). Assessment of technical and economical viability for large-scale conversion of single family residential buildings into zero energy buildings in Brazil: Climatic and cultural considerations. ENERGY POLICY, 63, 716–725. https://doi.org/10.1016/j.enpol.2013.07.133

Padovani, F., Sommerfeldt, N., Longobardi, F., & Pearce, J. (2021). Decarbonizing rural residential buildings in cold climates: A techno-economic analysis of heating electrification. ENERGY AND BUILDINGS, 250. https://doi.org/10.1016/j.enbuild.2021.111284

Páez, P., Araya, F., Salazar, L., Giménez, Z., Sánchez, O., Sierra-Varela, L., & Neculman, B. (2024). Exploring Perceptions toward Emotional Intelligence in Chilean Construction Using a Qualitative Approach. BUILDINGS, 14(4). https://doi.org/10.3390/buildings14040905

Paiho, S., & Ahvenniemi, H. (2017). Non-Technical Barriers to Energy Efficient Renovation of Residential Buildings and Potential Policy Instruments to overcome Them-Evidence from Young Russian Adults. Buildings, 7(4), 101. https://doi.org/10.3390/buildings7040101

Pallonetto, F. (2022). Advanced Energy Management Systems and Demand-Side Measures for Buildings towards the Decarbonisation of Our Society. In TRANSITIONING TO AFFORDABLE AND CLEAN ENERGY (WOS:001237316800006; Vol. 7, p. +). https://doi.org/10.3390/books978-3-03897-777-3

Panakaduwa, C., Coates, P., & Munir, M. (2024). Identifying sustainable retrofit challenges of historical Buildings: A systematic review. Energy and Buildings, 114226.

Passoni, C., Caruso, M., Marini, A., Pinho, R., & Landolfo, R. (2022). The Role of Life Cycle Structural Engineering in the Transition towards a Sustainable Building Renovation: Available Tools and Research Needs. Buildings, 12(8), 1107. https://doi.org/10.3390/buildings12081107

Pearce, A., Doshi, P., & Carneal, M. (2016). *Leverage Points for Sustainability: Targeting Stakeholders, Innovations, and Decision Types of Maximal Influence* (WOS:000389279902018). 1181–1191.

Peavey, J. B., Hudson, E., Summy, Z. A., & Violette, J. (2023). 3D Concrete Printed Houses: Barriers to Adoption and Construction Practices. *Cityscape*, *25*(1), 163–175.

Pedrosa, M., Arantes, A., & Cruz, C. (2023). Barriers to Adopting Lean Methodology in the Portuguese Construction Industry. BUILDINGS, 13(8). https://doi.org/10.3390/buildings13082047

Pelenur, M. J., & Cruickshank, H. J. (2014). Motivations to adopting energy efficiency measures in the home. Proceedings of the Institution of Civil Engineers. Energy, 167(3), 103–116. https://doi.org/10.1680/ener.14.00013

Perceived Benefits of Energy Efficiency in the Spanish Residential Market and Their Relation to Sociodemographic and Living Conditions. (2021). Applied Sciences, 11(2), 875. https://doi.org/10.3390/app11020875

Pérez-Navarro, J., Bueso, M., & Vázquez, G. (2023). Drivers of and Barriers to Energy Renovation in Residential Buildings in Spain-The Challenge of Next Generation EU Funds for Existing Buildings. BUILDINGS, 13(7). https://doi.org/10.3390/buildings13071817

Perry, C., PE, & Nadel, S. (2021). Electrifying Space Heating in Existing Commercial Buildings: Opportunities and Challenges. *ASHRAE Transactions*, *127*, 62–70.

Persson, J., & Grönkvist, S. (2015). Drivers for and barriers to low-energy buildings in Sweden. JOURNAL OF CLEANER PRODUCTION, 109, 296–304. https://doi.org/10.1016/j.jclepro.2014.09.094

Piantanida, P., Ostorero, C. L., Vottari, A., Manni, V., Valzano, L. S., Farnetani, R., & Surra, M. (2023). Strategies for a Positive Anthropogenic Impact in Postwar Buildings. Sustainability, 15(10), 7809. https://doi.org/10.3390/su15107809

Pimentel, M., Arantes, A., & Cruz, C. (2022). Barriers to the Adoption of Reverse Logistics in the Construction Industry: A Combined ISM and MICMAC Approach. SUSTAINABILITY, 14(23). https://doi.org/10.3390/su142315786

Pittri, H., Godawatte, A., Agyekum, K., Botchway, E., Dompey, A., Oduro, S., & Asamoah, E. (2024). Examining the barriers to implementing design for deconstruction in the construction industry of a developing country. CONSTRUCTION INNOVATION-ENGLAND. https://doi.org/10.1108/CI-09-2023-0239

Plavina, A., & Gruner, M. (2019). Selbukassa—A Case Study for Aiming at Low Emission Buildings through Extensive Reuse of Materials. IOP Conference Series. Earth and Environmental Science, 352(1). https://doi.org/10.1088/1755-1315/352/1/012067

Poncin Stéphane. (2021). Energy Policies for Eco-Friendly Households in Luxembourg: A Study Based on the LuxHEI Model. Environmental Modeling & Assessment, 26(1), 37–61. https://doi.org/10.1007/s10666-020-09725-7

Popescu, C., Apostu, S., Radulescu, I., Muresan, J., & Brezoi, A. (2024). Energizing the Now: Navigating the Critical Landscape of Today's Energy Challenges-An In-Depth Review. ENERGIES, 17(3). https://doi.org/10.3390/en17030675

Popovski, E., Aydemir, A., Fleiter, T., Bellstaedt, D., Büchele, R., & Steinbach, J. (2019). The role and costs of large-scale heat pumps in decarbonising existing district heating networks—A case study for the city of Herten in Germany. ENERGY, 180, 918–933. https://doi.org/10.1016/j.energy.2019.05.122

Prabatha, T., Hewage, K., & Sadiq, R. (2022). An Energy Performance Contract Optimization Approach to Meet the Competing Stakeholder Expectations under Uncertainty: A Canadian Case Study. Sustainability, 14(7), 4334. https://doi.org/10.3390/su14074334

Primozic, L., & Kutnar, A. (2024). Key decision factors of professional stakeholders (architects, engineers, constructors) when deciding for sustainable construction. FRONTIERS IN BUILT ENVIRONMENT, 10. https://doi.org/10.3389/fbuil.2024.1420163

Puček, M. M., Khoja, A., Bazzan, E., & Gyuris, P. (2023). A Data Structure for Digital Building Logbooks: Achieving Energy Efficiency, Sustainability, and Smartness in Buildings across the EU. Buildings, 13(4), 1082. https://doi.org/10.3390/buildings13041082

Qin Xiaoxiang, Yin Junjia, Nuzul Azam Haron, Alias, A. H., Law, T. H., & Nabilah Abu Bakar. (2024). Status, Challenges and Future Directions in the Evaluation of Net-Zero Energy Building Retrofits: A Bibliometrics-Based Systematic Review. Energies, 17(15), 3826. https://doi.org/10.3390/en17153826

Qiu, D., Xue, J., Zhang, T., Wang, J., & Sun, M. (2023). Federated reinforcement learning for smart building joint peer-to-peer energy and carbon allowance trading. APPLIED ENERGY, 333. https://doi.org/10.1016/j.apenergy.2022.120526

R P N P Weerasinghe, & Yang, R. J. (2022). Impact of technology developments on multicriteria performances of BIPV in non-domestic buildings. IOP Conference Series. Earth and Environmental Science, 1101(2), 022029. https://doi.org/10.1088/1755-1315/1101/2/022029

R.GREENAN1 & B. MUIR2. (2017). NEW ZEALAND'S BUILDING PERFORMANCE PATHWAYS. International Journal of Sustainable Development and Planning, 12(2), 252. https://doi.org/10.2495/SDP-V12-N2-252-263

Rahim, N., Ismail, S., Subramaniam, C., Habib, S., & Durdyev, S. (2023). Building Information Modelling Strategies in Sustainable Housing Construction Projects in Malaysia. SUSTAINABILITY, 15(3). https://doi.org/10.3390/su15032313

Rajabi, M., Sardroud, J., & Kheyroddin, A. (2021). Green standard model using machine learning: Identifying threats and opportunities facing the implementation of green building in Iran. ENVIRONMENTAL SCIENCE AND POLLUTION RESEARCH, 28(44), 62796–62808. https://doi.org/10.1007/s11356-021-14991-3

Rajendra, P., & Mohanasundaram, T. (2024). Factors driving consumer adoption of smart and green building materials: The role of civil engineers and architects. JOURNAL OF ASIAN ARCHITECTURE AND BUILDING ENGINEERING. https://doi.org/10.1080/13467581.2024.2373819

Rajski, K., Englart, S., & Sohani, A. (2024). Analysis of Greywater Recovery Systems in European Single-Family Buildings: Economic and Environmental Impacts. Sustainability, 16(12), 4912. https://doi.org/10.3390/su16124912

Raman, N. S., Devraj, A. M., Barooah, P., & Meyn, S. P. (2020). Reinforcement Learning for Control of Building HVAC Systems. The Institute of Electrical and Electronics Engineers, Inc. (IEEE) Conference Proceedings, 2326. https://doi.org/10.23919/ACC45564.2020.9147629

Rameezdeen, R., Zuo, J., & Stevens, J. (2017). Practices, drivers and barriers of implementing green leases: Lessons from South Australia. Journal of Corporate Real Estate, 19(1), 36–52. https://doi.org/10.1108/JCRE-04-2016-0018

Ramirez, J., Velazquez, D., & Velez-Zapata, C. (2022). The potential role of peace, justice, and strong institutions in Colombia?s areas of limited statehood for energy diversification towards governance in energy democracy. ENERGY POLICY, 168. https://doi.org/10.1016/j.enpol.2022.113135

Raouf, A., & Al-Ghamdi, S. (2019). Building information modelling and green buildings: Challenges and opportunities. ARCHITECTURAL ENGINEERING AND DESIGN MANAGEMENT, 15(1), 1–28. https://doi.org/10.1080/17452007.2018.1502655

Rasheed, A., Booth, C., & Horry, R. (2023). Stakeholder perceptions of the benefits and barriers of implementing environmental management systems in the Maldivian construction industry. JOURNAL OF HOUSING AND THE BUILT ENVIRONMENT, 38(4), 2821–2850. https://doi.org/10.1007/s10901-023-10067-5

Rauniyar, A., Singh, A., Kineber, A., Chandra, S., Awuzie, B., Kumar, V., Krishnaraj, L., Al-Otaibi, A., Alabdullatief, A., & Alfraidi, S. (2024). A strategic roadmap for combating barriers negating the implementation of prefabricated net-zero carbon buildings. DEVELOPMENTS IN THE BUILT ENVIRONMENT, 18. https://doi.org/10.1016/j.dibe.2024.100432

Razmjoo, A., Nezhad, M., Kaigutha, L., Marzband, M., Mirjalili, S., Pazhoohesh, M., Memon, S., Ehyaei, M., & Piras, G. (2021). Investigating Smart City Development Based on Green Buildings, Electrical Vehicles and Feasible Indicators. SUSTAINABILITY, 13(14). https://doi.org/10.3390/su13147808

Reda, F., Ruggiero, S., Auvinen, K., & Temmes, A. (2021). Towards low-carbon district heating: Investigating the socio-technical challenges of the urban energy transition. SMART ENERGY, 4. https://doi.org/10.1016/j.segy.2021.100054

Reindl, K., & Palm, J. (2021). Installing PV: Barriers and enablers experienced by non-residential property owners. RENEWABLE & SUSTAINABLE ENERGY REVIEWS, 141. https://doi.org/10.1016/j.rser.2021.110829

Reis, D., De Domenico, A., Lopes, L., & Almeida, M. (2023). Strategies and Actions for Achieving Carbon Neutrality in Portuguese Residential Buildings by 2050. SUSTAINABILITY, 15(21). https://doi.org/10.3390/su152115626

Reis, I. F. G., Gonçalves, I., Lopes, M. A. R., & Antunes, C. H. (2022). Collective self-consumption in multi-tenancy buildings–To what extent do consumers' goals influence the energy system's performance? Sustainable Cities and Society, 80, 103688. https://doi.org/10.1016/j.scs.2022.103688

Reyes-Veras, P., Renukappa, S., & Suresh, S. (2021). CHALLENGES FACED BY THE ADOPTION OF BIG DATA IN THE DOMINICAN REPUBLIC CONSTRUCTION INDUSTRY: AN EMPIRICAL STUDY. JOURNAL OF INFORMATION TECHNOLOGY IN CONSTRUCTION, 26, 812–831. https://doi.org/10.36680/j.itcon.2021.044

Rios, F., Grau, D., & Bilec, M. (2021). Barriers and Enablers to Circular Building Design in the US: An Empirical Study. JOURNAL OF CONSTRUCTION ENGINEERING AND MANAGEMENT, 147(10). https://doi.org/10.1061/(ASCE)CO.1943-7862.0002109

Roadblocks to Low Temperature District Heating. (2020). Energies, 13(22), 5893. https://doi.org/10.3390/en13225893

Rocha, L., Rui Fernandes Póvoas, & Restivo, J. (2023). The Right to Comfort in Social Housing: Energy and Thermal Performances as Parameters of a Systemic Analysis. Buildings, 13(5), 1173. https://doi.org/10.3390/buildings13051173

Rock, S., Hosseini, M. R., Nikmehr, B., Martek, I., Abrishami, S., & Durdyev, S. (2019). Barriers to "green operation" of commercial office buildings; Perspectives of Australian facilities managers. Facilities, 37(13/14), 1048–1065. https://doi.org/10.1108/F-08-2018-0101

Rodrigues, A. M., Olubimbola Oladimeji, Guedes, A. L. A., Chinelli, C. K., Haddad, A. N., & Soares, C. A. P. (2023). The Project Manager's Core Competencies in Smart Building Project Management. Buildings, 13(8), 1981. https://doi.org/10.3390/buildings13081981

Rodriguez, N., Katooziani, A., & Jeelani, I. (2024). Barriers to energy-efficient design and construction practices: A comprehensive analysis. JOURNAL OF BUILDING ENGINEERING, 82. https://doi.org/10.1016/j.jobe.2023.108349

Romero-Lankao, P., Wilson, A., Sperling, J., Miller, C., Zimny-Schmitt, D., Sovacool, B., Gearhart, C., Muratori, M., Bazilian, M., Zünd, D., Young, S., Brown, M., & Arent, D. (2021). Of actors, cities and energy systems: Advancing the transformative potential of urban electrification. PROGRESS IN ENERGY, 3(3). https://doi.org/10.1088/2516-1083/abfa25

Romo, R., Alejo-Reyes, A., & Orozco, F. (2024). Statistical Analysis of Lean Construction Barriers to Optimize Its Implementation Using PLS-SEM and PCA. BUILDINGS, 14(2). https://doi.org/10.3390/buildings14020486

Rose, C., & Stegemann, J. (2019). Characterising existing buildings as material banks (E-BAMB) to enable component reuse. PROCEEDINGS OF THE INSTITUTION OF CIVIL ENGINEERS-ENGINEERING SUSTAINABILITY, 172(3), 129–140. https://doi.org/10.1680/jensu.17.00074

Rosner, Y., Amitay, Z., & Perlman, A. (2022). Consumer's attitude, socio-demographic variables and willingness to purchase green housing in Israel. ENVIRONMENT DEVELOPMENT AND SUSTAINABILITY, 24(4), 5295–5316. https://doi.org/10.1007/s10668-021-01659-8

Rotimi, F., Almughrabi, F., Samarasinghe, D., & Silva, C. (2022). Specific Skill Requirements within Prefabricated Residential Construction: Stakeholders' Perspectives. BUILDINGS, 12(1). https://doi.org/10.3390/buildings12010043

Rozwadowska, M., & Szymanski, P. (2019). *Barriers that Limit the Scaling of RES Projects in Polish Cities* (WOS:000561117205083). 11583–11595.

Ruá, M. J., Pitarch, Á. M., Arín, I., & Reig, L. (2024). A Roof Refurbishment Strategy to Improve the Sustainability of Building Stock: A Case Study. Sustainability, 16(5), 2028. https://doi.org/10.3390/su16052028

Ruffino, E., Piga, B., Casasso, A., & Sethi, R. (2022). Heat Pumps, Wood Biomass and Fossil Fuel Solutions in the Renovation of Buildings: A Techno-Economic Analysis Applied to Piedmont Region (NW Italy). Energies, 15(7), 2375. https://doi.org/10.3390/en15072375

Ruiz, S., Jimenez, J., & Carvajal, S. (2022). Zero Energy Balance Buildings: Definitions, Current Challenges and Future Opportunities. IEEE LATIN AMERICA TRANSACTIONS, 20(3), 417–429. https://doi.org/10.1109/TLA.2022.9667140

Rumsey, P., PE, Garrec, J., & Levasseur, A., PE. (2021). How Building Decarbonization Can Transform HVAC. *ASHRAE Journal*, *63*(9), 14–27.

Saadatian, A., & Olbina, S. (2024). Barriers to the implementation of energy-efficiency practices in the US retail sector: Facilities managers' perceptions compared by facility size and business revenue. FACILITIES, 42(7/8), 622–640. https://doi.org/10.1108/F-11-2023-0095

Sabri, R., & Olagoke, O. (2021). Safeguarding the colonial era's ecclesiastical heritage: Towards a sustainable protection-use model. JOURNAL OF CULTURAL HERITAGE MANAGEMENT AND SUSTAINABLE DEVELOPMENT, 11(1), 121–134. https://doi.org/10.1108/JCHMSD-01-2020-0017

Sachs, J., Schmidt-Traub, G., Mazzucato, M., Messner, D., Nakicenovic, N., & Rockström, J. (2019). Six Transformations to achieve the Sustainable Development Goals. NATURE SUSTAINABILITY, 2(9), 805–814. https://doi.org/10.1038/s41893-019-0352-9

Safonov, G., Potashnikov, V., Lugovoy, O., Safonov, M., Dorina, A., & Bolotov, A. (2020). The low carbon development options for Russia. Climatic Change, 162(4), 1929–1945. https://doi.org/10.1007/s10584-020-02780-9

Saha, S., Hiremath, R., & Sanjay, P. (2022). Barriers to adoption of green buildings – a review. Cardiometry, 22, 377–385. https://doi.org/10.18137/cardiometry.2022.22.377385

Sajid, Z., Aftab, U., & Ullah, F. (2024). Barriers to adopting circular procurement in the construction industry: The way forward. SUSTAINABLE FUTURES, 8. https://doi.org/10.1016/j.sftr.2024.100244

Sakr, D., Sherif, A., & El-Haggar, S. (2010). Environmental management systems' awareness: An investigation of top 50 contractors in Egypt. JOURNAL OF CLEANER PRODUCTION, 18(3), 210–218. https://doi.org/10.1016/j.jclepro.2009.09.021

Salazar, J., Guevara, J., Espinosa, M., Rivera, F., & Franco, J. (2022). Decarbonization of the Colombian Building Sector: Social Network Analysis of Enabling Stakeholders. BUILDINGS, 12(10). https://doi.org/10.3390/buildings12101531

Saliu, L., Monko, R., Zulu, S., & Maro, G. (2024). Barriers to the Integration of Building Information Modeling (BIM) in Modular Construction in Sub-Saharan Africa. BUILDINGS, 14(8). https://doi.org/10.3390/buildings14082448

Saloux, E., Zhang, K., & Candanedo, J. A. (2023). A Critical Perspective on Current Research Trends in Building Operation: Pressing Challenges and Promising Opportunities. Buildings, 13(10), 2566. https://doi.org/10.3390/buildings13102566

Samarasinghe, D., Baghaei, N., & Stemmet, L. (2020). Persuasive Virtual Reality: Promoting Earth Buildings in New Zealand (WOS:000788445100016). 12064, 208–220. https://doi.org/10.1007/978-3-030-45712-9_16

Sánchez-García, L., Averfalk, H., & Persson, U. (2021). Further investigations on the Effective Width for district heating systems. ENERGY REPORTS, 7, 351–358. https://doi.org/10.1016/j.egyr.2021.08.096

Sandin, S., Neij, L., & Mickwitz, P. (2019). Transition governance for energy efficiency—Insights from a systematic review of Swedish policy evaluation practices. Energy, Sustainability and Society, 9(1), 1–18. https://doi.org/10.1186/s13705-019-0203-6

Sanga, S. A. (2020). Compliance with Building Material Specifications among Informal Skilled Construction Workers in Dar es Salaam, Tanzania. Journal of Construction in Developing Countries, 25(2), 63–91. https://doi.org/10.21315/jcdc2020.25.2.3

Santana-Sosa, A., & Kovacic, I. (2022). Barriers, Opportunities and Recommendations to Enhance the Adoption of Timber within Multi-Storey Buildings in Austria. Buildings, 12(9), 1416. https://doi.org/10.3390/buildings12091416

Santos, P., Génesis Camila Cervantes, Zaragoza-Benzal, A., Byrne, A., Karaca, F., Ferrández, D., Salles, A., & Bragança, L. (2024). Circular Material Usage Strategies and Principles in Buildings: A Review. Buildings, 14(1), 281. https://doi.org/10.3390/buildings14010281

Sao, A., Goswami, A., Verma, A., Kataria, R., & Shahi, R. (2023). Challenges in the adoption of building information technology (BIM) technology in Indian construction industry (ICI). JOURNAL OF INFORMATION & OPTIMIZATION SCIENCES, 44(8), 1577–1593. https://doi.org/10.47974/JIOS-1477

Sarafidis, Y., Demertzis, N., Georgopoulou, E., Avrami, L., Mirasgedis, S., & Kaminiaris, O. (2024). Socioeconomic Impacts of Climate Mitigation Actions in Greece: Quantitative Assessment and Public Perception. ATMOSPHERE, 15(4). https://doi.org/10.3390/atmos15040454

Sardroud, J. M., Mehdizadehtavasani, M., Khorramabadi, A., & Ranjbardar, A. (2018). Barriers Analysis to Effective Implementation of BIM in the Construction Industry. *ISARC. Proceedings of the International Symposium on Automation and Robotics in Construction*, *35*, 1–8.

Sasges, G., & Ziegler, A. (2023). We Have Eaten the Rivers: The Past, Present, and Unsustainable Future of Hydroelectricity in Vietnam. SUSTAINABILITY, 15(11). https://doi.org/10.3390/su15118969

Scamman, D., Solano-Rodríguez, B., Pye, S., Chiu, L., Smith, A., Cassarino, T., Barrett, M., & Lowe, R. (2020). Heat Decarbonisation Modelling Approaches in the UK: An Energy System Architecture Perspective. ENERGIES, 13(8). https://doi.org/10.3390/en13081869

Schaffert, M., Warch, D., & Müller, H. (2024). A Planning Support System for Monitoring Aging Neighborhoods in Germany. Geomatics, 4(1), 66. https://doi.org/10.3390/geomatics4010004

Scherz, M., Kreiner, H., Alaux, N., & Passer, A. (2023). Transition of the procurement process to Paris-compatible buildings: Consideration of environmental life cycle costing in tendering and awarding. The International Journal of Life Cycle Assessment, 28(7), 843–861. https://doi.org/10.1007/s11367-023-02153-1

Scherz, M., Wieser, A. A., Passer, A., & Kreiner, H. (2022). Implementation of Life Cycle Assessment (LCA) in the Procurement Process of Buildings: A Systematic Literature Review. Sustainability, 14(24), 16967. https://doi.org/10.3390/su142416967

Schildt, J., Booth, C., Horry, R., & Wiejak-Roy, G. (2023). Stakeholder Opinions of Implementing Environmental Management Systems in the Construction Sector of the US. BUILDINGS, 13(5). https://doi.org/10.3390/buildings13051241

Schmidt, J., & Griffin, C. (2013). *Barriers to the design and use of cross-laminated timber structures in high-rise multi-family housing in the United States* (WOS:000350208600277). 2225–2231.

Schwartz, E. K. (n.d.). *ENERGY AND COST OPTIMIZATION FOR CASE STUDY IN BOULDER, COLORADO*.

Schwartz, E. K., & Krarti, M. (2022). Review of Adoption Status of Sustainable Energy Technologies in the US Residential Building Sector. Energies, 15(6), 2027. https://doi.org/10.3390/en15062027

Schweber, L., & Leiringer, R. (2012). Beyond the technical: A snapshot of energy and buildings research. BUILDING RESEARCH AND INFORMATION, 40(4), 481–492. https://doi.org/10.1080/09613218.2012.675713

Scipioni, S., Russ, M., & Niccolini, F. (2021). From Barriers to Enablers: The Role of Organizational Learning in Transitioning SMEs into the Circular Economy. Sustainability, 13(3), 1021. https://doi.org/10.3390/su13031021

Sedighi, M., Qhazvini, P. P., & Amidpour, M. (2023). Algae-Powered Buildings: A Review of an Innovative, Sustainable Approach in the Built Environment. Sustainability, 15(4), 3729. https://doi.org/10.3390/su15043729

Sesana, M. M., Rivallain, M., & Graziano Salvalai. (2020). Overview of the Available Knowledge for the Data Model Definition of a Building Renovation Passport for Non-Residential Buildings: The ALDREN Project Experience. Sustainability, 12(2), 642. https://doi.org/10.3390/su12020642

Shah, S. (2012). Sustainable Refurbishment. Sustainable Refurbishment. https://ezproxy.bu.edu/login?qurl=https://www.proquest.com/docview/2131181696?accountid=9676&bdid=77468&_bd=vpVlqiaDQwhTLb16TZ3QHA2uLW4%3D

Shaker, M., Eustace, B., Erukala, H., Patel, R., Mohammed, M., Jabri, M., Desai, K., Goyal, R., & Chang, B. (2022). Analysis of Survey on Barriers to the Implementation of Sustainable Projects. SUSTAINABILITY, 14(24). https://doi.org/10.3390/su142416830

Shapiro, S. (2016). The realpolitik of building codes: Overcoming practical limitations to climate resilience. BUILDING RESEARCH AND INFORMATION, 44(5–6), 490–506. https://doi.org/10.1080/09613218.2016.1156957

Sheikh Zuhaib, Manton, R., Hajdukiewicz, M., Keane, M. M., & Goggins, J. (2017). Attitudes and approaches of Irish retrofit industry professionals towards achieving nearly zero-energy buildings. International Journal of Building Pathology and Adaptation, 35(1), 16–40. https://doi.org/10.1108/IJBPA-07-2016-0015

Sheikh, N., Laverge, J., & Delghust, M. (2024). A Critical Analysis of Institutional and Regulatory Framework for Building Stock Energy Efficiency and Transition in Pakistan. *Environmental Science & Sustainable Development*, *9*(1), 32–41. https://doi.org/10.21625/essd.v9i1.1066

Shen, L., Yang, J., Zhang, R., Shao, C., & Song, X. (2019). The Benefits and Barriers for Promoting Bamboo as a Green Building Material in ChinaAn Integrative Analysis. SUSTAINABILITY, 11(9). https://doi.org/10.3390/su11092493

Sherif, M., Abotaleb, I., & Alqahtani, F. (2022). Application of Integrated Project Delivery (IPD) in the Middle East: Implementation and Challenges. BUILDINGS, 12(4). https://doi.org/10.3390/buildings12040467

Sherman, R., Naganathan, H., & Parrish, K. (2021). Energy Savings Results from Small Commercial Building Retrofits in the US. Energies, 14(19), 6207. https://doi.org/10.3390/en14196207

Shevchenko, A., Linovsky, S., & Skrobot, O. (2020). Geothermal Energy Use in Western Siberia and Prospects for Its Innovative Application in Construction. IOP Conference Series. Materials Science and Engineering, 953(1). https://doi.org/10.1088/1757-899X/953/1/012006

Shi, Q., Zuo, J., Huang, R., Huang, J., & Pullen, S. (2013). Identifying the critical factors for green construction—An empirical study in China. HABITAT INTERNATIONAL, 40, 1–8. https://doi.org/10.1016/j.habitatint.2013.01.003

Shibeika, A., T Abdel Raheem, & Hittini, B. (2019). Towards conceptual understanding for the adoption of building environmental sustainability assessment methods in the UAE built environment. IOP Conference Series. Earth and Environmental Science, 323(1). https://doi.org/10.1088/1755-1315/323/1/012185

Shilei, L., & Yong, W. (2009). Target-oriented obstacle analysis by PESTEL modeling of energy efficiency retrofit for existing residential buildings in China's northern heating region. Energy Policy, 37(6), 2098–2101. https://doi.org/10.1016/j.enpol.2008.11.039

Shindo, K., Ikai, K., Shinoda, J., Matsumura, R., & Tanabe, S. (2024). Design and control of radiant heating and cooling systems in Japan: Results from expert interviews. JAPAN ARCHITECTURAL REVIEW, 7(1). https://doi.org/10.1002/2475-8876.12451

Shooshtarian, S., Hosseini, M., Kocaturk, T., Arnel, T., & Garofano, N. (2023). Circular economy in the Australian AEC industry: Investigation of barriers and enablers. BUILDING RESEARCH AND INFORMATION, 51(1), 56–68. https://doi.org/10.1080/09613218.2022.2099788

Shooshtarian, S., Maqsood, T., Caldera, S., & Ryley, T. (2022). The impact of regulations on construction and demolition waste market creation and stimulation: Australian stakeholders' perception. IOP Conference Series. Earth and Environmental Science, 1101(6), 062012. https://doi.org/10.1088/1755-1315/1101/6/062012

Shooshtarian, S., Maqsood, T., Wong, P., Caldera, S., Ryley, T., Zaman, A., & Ruiz, A. (2024). Circular economy in action: The application of products with recycled content in construction projects—A multiple case study approach. SMART AND SUSTAINABLE BUILT ENVIRONMENT, 13(2), 370–394. https://doi.org/10.1108/SASBE-08-2023-0213

Simpeh, E., Smallwood, J., Ahadzie, D., & Mensah, H. (2023). Analytical taxonomy of challenges to the implementation of green building projects in South Africa. INTERNATIONAL JOURNAL OF CONSTRUCTION MANAGEMENT, 23(2), 286–296. https://doi.org/10.1080/15623599.2020.1863172

Simpson, K., Murtagh, N., & Owen, A. (2021). Domestic retrofit: Understanding capabilities of micro-enterprise building practitioners. Buildings & Cities, 2(1), 449. https://doi.org/10.5334/bc.106

Sin, O., Yusof, N., & Osmadi, A. (2021). Challenges of Green Office Implementation: A Case Study in Penang, Malaysia. INTERNATIONAL JOURNAL OF SUSTAINABLE CONSTRUCTION ENGINEERING AND TECHNOLOGY, 12(1), 153–163. https://doi.org/10.30880/ijscet.2021.12.01.015

Singh, A., Kumar, V., Hu, J., & Irfan, M. (2023). Investigation of barriers and mitigation strategies to blockchain technology implementation in construction industry: An interpretive structural modeling approach. ENVIRONMENTAL SCIENCE AND POLLUTION RESEARCH, 30(38), 89889–89909. https://doi.org/10.1007/s11356-023-28749-6

Singh, A., Kumar, V., Shoaib, M., Adebayo, T., & Irfan, M. (2023). A strategic roadmap to overcome blockchain technology barriers for sustainable construction: A deep learning-based dual-stage SEM-ANN approach. TECHNOLOGICAL FORECASTING AND SOCIAL CHANGE, 194. https://doi.org/10.1016/j.techfore.2023.122716

Singh, R., Walsh, P., & Mazza, C. (2019). Sustainable Housing: Understanding the Barriers to Adopting Net Zero Energy Homes in Ontario, Canada. SUSTAINABILITY, 11(22). https://doi.org/10.3390/su11226236

Siva, V., Hoppe, T., & Jain, M. (2017). Green Buildings in Singapore; Analyzing a Frontrunner's Sectoral Innovation System. SUSTAINABILITY, 9(6). https://doi.org/10.3390/su9060919

Smedby, N. (2020). Limits to polycentricity? Institutional layering and policy feedbacks of building energy performance requirements in Sweden. ENVIRONMENTAL POLICY AND GOVERNANCE, 30(2), 71–83. https://doi.org/10.1002/eet.1874

Sodangi, M., & Kazmi, Z. (2020). Integrated Evaluation of the Impediments to the Adoption of Coconut Palm Wood as a Sustainable Material for Building Construction. SUSTAINABILITY, 12(18). https://doi.org/10.3390/su12187676

Soorige, D., Karunasena, G., Kulatunga, U., Mahmood, M. N., & De Silva, L. (2022). An Energy Culture Maturity Conceptual Framework on Adopting Energy-Efficient Technology Innovations in Buildings. Journal of Open Innovation : Technology, Market, and Complexity, 8(2), 60. https://doi.org/10.3390/joitmc8020060

Souaid, C., van der Heijden, H., & Elsinga, M. (2022). Perceived Barriers to Nearly Zero-Energy Housing: Empirical Evidence from Kilkenny, Ireland. Energies, 15(17), 6421. https://doi.org/10.3390/en15176421

Souleymane, D., & Ghiaus, C. (2023). Multi-Criteria Decision Analysis for Energy Retrofit of Residential Buildings: Methodology and Feedback from Real Application. Energies, 16(2), 902. https://doi.org/10.3390/en16020902

Soultana (Tanya) Saroglou, Itzhak-Ben-Shalom, H., & Meir, I. A. (2023). Climatic Variability in Altitude: Architecture, Thermal Comfort, and Safety along the Facade of a Residential Tower in the Mediterranean Climate. Buildings, 13(8), 1979. https://doi.org/10.3390/buildings13081979

Sourani, A., & Sohail, M. (2011). Barriers to addressing sustainable construction in public procurement strategies. PROCEEDINGS OF THE INSTITUTION OF CIVIL ENGINEERS-ENGINEERING SUSTAINABILITY, 164(4), 229–237. https://doi.org/10.1680/ensu.2011.164.4.229

Sovacool, B. (2017). Contestation, contingency, and justice in the Nordic low-carbon energy transition. ENERGY POLICY, 102, 569–582. https://doi.org/10.1016/j.enpol.2016.12.045

Sovacool, B., & Brossmann, B. (2010). Symbolic convergence and the hydrogen economy. ENERGY POLICY, 38(4), 1999–2012. https://doi.org/10.1016/j.enpol.2009.11.081

Staller, H., Tritthart, W., & Hajpál, M. (2010). *LCA - USE AND BARRIERS* (WOS:000288408300146). 617–620.

Stemmle, R., Hanna, R., Menberg, K., Østergaard, P. A., Jackson, M., Staffell, I., & Blum, P. (2024). Policies for aquifer thermal energy storage: International comparison, barriers and recommendations. Clean Technologies and Environmental Policy. https://doi.org/10.1007/s10098-024-02892-1

Stevenson, F., & Baborska-Narozny, M. (2018). Housing performance evaluation: Challenges for international knowledge exchange. Building Research and Information, 46(5), 501. https://doi.org/10.1080/09613218.2017.1357095

Stojanovska-Georgievska, L., Sandeva, I., & Spasevska, H. (2017). *An Empirical Survey on the Awareness of Construction Developers about Green Buildings in Macedonia* (WOS:000426982300038). 203–209.

Stojanovska-Georgievska, L., Sandeva, I., & Spasevska, H. (2018). AN EMPIRICAL SURVEY ON THE AWARENESS OF CONSTRUCTION DEVELOPERS ABOUT GREEN BUILDINGS IN MACEDONIA. THERMAL SCIENCE, 22, S897–S907. https://doi.org/10.2298/TSCI170915023S

Strachan, N. (2011). UK energy policy ambition and UK energy modelling-fit for purpose? ENERGY POLICY, 39(3), 1037–1040. https://doi.org/10.1016/j.enpol.2011.01.015

Streimikiene, D., & Balezentis, T. (2020). Willingness to Pay for Renovation of Multi-Flat Buildings and to Share the Costs of Renovation. ENERGIES, 13(11). https://doi.org/10.3390/en13112721

Struhala, K., & Ostry, M. (2022). Life-Cycle Assessment of phase-change materials in buildings: A review. JOURNAL OF CLEANER PRODUCTION, 336. https://doi.org/10.1016/j.jclepro.2022.130359

Suleman, T., Ezema, I., & Aderonmu, P. (2023). Challenges of circular design adoption in the Nigerian built environment: An empirical study. CLEANER ENGINEERING AND TECHNOLOGY, 17. https://doi.org/10.1016/j.clet.2023.100686

Sullivan, P. (2014). REDUCING ENERGY CONSUMPTION IN AMERICA'S GOVERNMENT BUILDINGS. *Proceedings of the International Annual Conference of the American Society for Engineering Management*, 1–1.

Sun, H., Fang, Y., Yin, M., & Shi, F. (2023). Research on the Restrictive Factors of Vigorous Promotion of Prefabricated Buildings in Yancheng under the Background of "Double Carbon." SUSTAINABILITY, 15(2). https://doi.org/10.3390/su15021737

Sun, Y., Wang, J., Wu, J., Shi, W., Ji, D., Wang, X., & Zhao, X. (2020). Constraints Hindering the Development of High-Rise Modular Buildings. APPLIED SCIENCES-BASEL, 10(20). https://doi.org/10.3390/app10207159

Susskind, L., Chun, J., Gant, A., Hodgkins, C., Cohen, J., & Lohmar, S. (2022). Sources of opposition to renewable energy projects in the United States. ENERGY POLICY, 165. https://doi.org/10.1016/j.enpol.2022.112922

Iqbal, M., Ma, J., Ahmad, N., Hussain, K., Usmani, M. S., & Ahmad, M. (2021). Sustainable construction through energy management practices in developing economies: an analysis of barriers in the construction sector. *Environmental Science and Pollution Research*, *28*, 34793-34823.

Svensson, I. (2021). Institutional Work Across Multiple Levels: The Case of Strategic Public Facilities Management in the Making (Doctoral dissertation, Chalmers Tekniska Hogskola (Sweden)).

Taat, N., Abas, N., & Hasmori, M. (2022). The Barriers of Building Information Modelling (BIM) for Construction Safety (WOS:000774710100015). 214, 121–130. https://doi.org/10.1007/978-981-16-7920-9_15

Tafazzoli, M., Shrestha, K., & Dang, H. (2024). *Investigating Barriers to the Application of Automation in the Construction Industry* (WOS:001196835000096). 941–950.

Taherkhani, R. (2023). Barriers to green building implementation in developing countries: The case of Iran. ENVIRONMENT DEVELOPMENT AND SUSTAINABILITY. https://doi.org/10.1007/s10668-023-03816-7

Tajani, F., Morano, P., Felicia Di Liddo, & Doko, E. (2022). A Model for the Assessment of the Economic Benefits Associated with Energy Retrofit Interventions: An Application to Existing Buildings in the Italian Territory. Applied Sciences, 12(7), 3385. https://doi.org/10.3390/app12073385

Tajima, N. (2022). Research on the Regeneration Procedure for Reutilizing Vacant Housing Stock in Japan. IOP Conference Series. Earth and Environmental Science, 1101(4), 042021. https://doi.org/10.1088/1755-1315/1101/4/042021

Takyi-Annan, G., & Zhang, H. (2023). Assessing the impact of overcoming BIM implementation barriers on BIM usage frequency and circular economy in the project lifecycle using Partial least Squares structural Equation modelling (PLS-SEM) analysis. ENERGY AND BUILDINGS, 295. https://doi.org/10.1016/j.enbuild.2023.113329

Talele, S., Traylor, C., Arpan, L., Curley, C., Chien-Fei, C., Day, J., Feiock, R., Hadzikadic, M., Tolone, W. J., Ingman, S., Yeatts, D., Karaguzel, O. T., Lam, K. P., Menassa, C., Pevnitskaya, S., Spiegelhalter, T., Yan, W., Zhu, Y., & Tao, Y. X. (2018). Energy modeling and data structure framework for Sustainable Human-Building Ecosystems (SHBE)—A review. Frontiers in Energy, 12(2), 314–332. https://doi.org/10.1007/s11708-017-0530-2

Tarpio, J., Huuhka, S., & Vestergaard, I. (2022). Barriers to implementing adaptable housing: Architects' perceptions in Finland and Denmark. Journal of Housing and the Built Environment, 37(4), 1859–1881. https://doi.org/10.1007/s10901-021-09913-1

Tehami, M., & Seddiki, M. (2023). Investigation toward the Adoption of Building Information Modelling in Algeria from Architects' Perspective. Journal of Construction in Developing Countries, 28(2), 329–352. https://doi.org/10.21315/jcdc-08-22-0148

Teixeira, I., Ferreira, A. C., Rodrigues, N., & Teixeira, S. (2024). Energy Poverty and Its Indicators: A Multidimensional Framework from Literature. Energies, 17(14), 3445. https://doi.org/10.3390/en17143445

Terblanche, R. (2019). Achieving net zero status in South Africa (WOS:000562136800181). 323. https://doi.org/10.1088/1755-1315/323/1/012182

Teresa Diaz Gonçalves & José Saporiti Machado. (2023). Origins of the Sustainability Concept and Its Application to the Construction Sector in the EU. Sustainability, 15(18), 13775. https://doi.org/10.3390/su151813775

Thao Thi Phuong Bui, Wilkinson, S., Niluka Domingo, & MacGregor, C. (2021). Zero Carbon Building Practices in Aotearoa New Zealand. Energies, 14(15), 4455. https://doi.org/10.3390/en14154455

The role of European municipalities and regions in financing energy upgrades in buildings. (n.d.). *26*.

Thirumal, S., Udawatta, N., Karunasena, G., & Al-Ameri, R. (2024). Barriers to Adopting Digital Technologies to Implement Circular Economy Practices in the Construction Industry: A Systematic Literature Review. Sustainability, 16(8), 3185.

Thomas, T., & Praveen, A. (2020). Emergy parameters for ensuring sustainable use of building materials. JOURNAL OF CLEANER PRODUCTION, 276. https://doi.org/10.1016/j.jclepro.2020.122382

Thomson, L., & Jenkins, D. (2023). The Use of Real Energy Consumption Data in Characterising Residential Energy Demand with an Inventory of UK Datasets. Energies, 16(16), 6069. https://doi.org/10.3390/en16166069

Tian, L., Wright, A., Painter, B., & Pazhoohesh, M. (2023). Factors influencing BIM use in green building construction project management in the UK and China. BUILDING RESEARCH AND INFORMATION, 51(7), 853–870. https://doi.org/10.1080/09613218.2023.2213356

Tigani, D., D van Kan, Tennakoon, G., Geng, L., & Chan, M. (2024). Measuring Embodied Carbon of Buildings: A Review of Methodologies and Benchmarking Towards Net Zero. IOP Conference Series. Earth and Environmental Science, 1363(1), 012030. https://doi.org/10.1088/1755-1315/1363/1/012030

Timilsina, G. R., & Malla, S. (2023). Do Investments in Clean Technologies Reduce Production Costs? Insights from the Literature. Economics of Energy & Environmental Policy, 12(1). https://doi.org/10.5547/2160-5890.12.1.gtim

Tirelli, D., & Besana, D. (2023). Moving toward Net Zero Carbon Buildings to Face Global Warming: A Narrative Review. BUILDINGS, 13(3). https://doi.org/10.3390/buildings13030684

Topriska, E., Kolokotroni, M., Melandri, D., McGuiness, S., Ceclan, A., Christoforidis, G., Fazio, V., Hadjipanayi, M., Hendrick, P., Kacarska, M., Lopez, E., Petersen, K., & Steinbrecher, J. (2018). The Social, Educational, and Market Scenario for nZEB in Europe. BUILDINGS, 8(4). https://doi.org/10.3390/buildings8040051

Torbaghan, S., Gibescu, M., Rawn, B., Müller, H., Roggenkamp, M., & van der Meijden, M. (2015). Investigating the impact of unanticipated market and construction delays on the development of a meshed HVDC grid using dynamic transmission planning. IET GENERATION TRANSMISSION & DISTRIBUTION, 9(15), 2224–2233. https://doi.org/10.1049/iet-gtd.2014.1068

Torcellini, P., PhD PE, MacCracken, M., PE, Narayanamurthy, R., Deru, M., PhD, & Lowe, B. (2021). Energy Storage: Helping Move Toward A Sustainable Future. *ASHRAE Journal*, *63*(7), 24–32.

Torgautov, B., Zhanabayev, A., Tleuken, A., Turkyilmaz, A., Mustafa, M., & Karaca, F. (2021). Circular Economy: Challenges and Opportunities in the Construction Sector of Kazakhstan. Buildings, 11(11), 501. https://doi.org/10.3390/buildings11110501

Toriola-Coker, L. O., Alaka, H., Bello, W. A., Ajayi, S., Adeniyi, A., & Olopade, S. O. (2021). Sustainability Barriers in Nigeria Construction Practice. IOP Conference Series. Materials Science and Engineering, 1036(1). https://doi.org/10.1088/1757-899X/1036/1/012023

Labaran, Y. H., Mato, H., Saini, G., & Musa, A. A. (2024). Towards net zero energy buildings: A review of barriers and facilitators to the adoption of building energy efficiency practices. Environmental Research and Technology, 7(1), 118-130.

Toxopeus, H., & Polzin, F. (2021). Reviewing financing barriers and strategies for urban nature-based solutions. JOURNAL OF ENVIRONMENTAL MANAGEMENT, 289. https://doi.org/10.1016/j.jenvman.2021.112371

Toyin, J., & Mewomo, M. (2023). An investigation of barriers to the application of building information modelling in Nigeria. JOURNAL OF ENGINEERING DESIGN AND TECHNOLOGY, 21(2), 442–468. https://doi.org/10.1108/JEDT-10-2021-0594

Tozer, L. (2019). Deep Decarbonization in Practice: Solutions and Challenges for Low-Carbon Building Retrofi ts. *Canadian Journal of Urban Research*, *28*(2), 32–45.

Tozer, L. (2020). Catalyzing political momentum for the effective implementation of decarbonization for urban buildings. ENERGY POLICY, 136. https://doi.org/10.1016/j.enpol.2019.111042

Tran, Q., & Huang, D. (2022). Using PLS-SEM to analyze challenges hindering success of green building projects in Vietnam. JOURNAL OF ECONOMICS AND DEVELOPMENT, 24(1), 47–64. https://doi.org/10.1108/JED-04-2020-0033

Travezan, J., Harmsen, R., & van Toledo, G. (2013). Policy analysis for energy efficiency in the built environment in Spain. ENERGY POLICY, 61, 317–326. https://doi.org/10.1016/j.enpol.2013.05.096

Trencher, G., Broto, V., Takagi, T., Sprigings, Z., Nishida, Y., & Yarime, M. (2016). Innovative policy practices to advance building energy efficiency and retrofitting: Approaches, impacts and challenges in ten C40 cities. ENVIRONMENTAL SCIENCE & POLICY, 66, 353–365. https://doi.org/10.1016/j.envsci.2016.06.021

Triolo, R. C. (n.d.). *EFFICIENT ELECTRICITY SYSTEMS UNDER HIGH PENETRATION OF RENEWABLE AND DISTRIBUTED RESOURCES*.

Tsoumanis, G., Formiga, J., Bilo, N., Tsarchopoulos, P., Ioannidis, D., & Tzovaras, D. (2021). The Smart Evolution of Historical Cities: Integrated Innovative Solutions Supporting the Energy Transition while Respecting Cultural Heritage. Sustainability, 13(16), 9358. https://doi.org/10.3390/su13169358

Tuerk, A., Frieden, D., Neumann, C., Latanis, K., Tsitsanis, A., Kousouris, S., Llorente, J., Heimonen, I., Reda, F., Ala-Juusela, M., Allaerts, K., Caerts, C., Schwarzl, T., Ulbrich, M., Stosch, A., & Ramschak, T. (2021). Integrating Plus Energy Buildings and Districts with the EU Energy Community Framework: Regulatory Opportunities, Barriers and Technological Solutions. Buildings, 11(10), 468. https://doi.org/10.3390/buildings11100468

Tykkä, S., McCluskey, D., Nord, T., Ollonqvist, P., Hugosson, M., Roos, A., Ukrainski, K., Nyrud, A., & Bajric, F. (2010). Development of timber framed firms in the construction sector—Is EU policy one source of their innovation? FOREST POLICY AND ECONOMICS, 12(3), 199–206. https://doi.org/10.1016/j.forpol.2009.10.003

Udeaja, C., Ekundayo, D., Zhou, L., & Perera, S. (2013). Material Waste in the Construction Industry: A Review of the Legislative and Supply Chain Issues. In REUSE OF MATERIALS AND BYPRODUCTS IN CONSTRUCTION: WASTE MINIMIZATION AND RECYCLING (WOS:000332603200003; pp. 5–27). https://doi.org/10.1007/978-1-4471-5376-4_210.1007/978-1-4471-5376-4

Ullah, K., Witt, E., & Lill, I. (2022). The BIM-Based Building Permit Process: Factors Affecting Adoption. Buildings, 12(1), 45. https://doi.org/10.3390/buildings12010045

Unuigbe, M., Sambo Lyson Zulu, & Johnston, D. (2022). Exploring Factors Influencing Renewable Energy Diffusion in Commercial Buildings in Nigeria: A Grounded Theory Approach. Sustainability, 14(15), 9726. https://doi.org/10.3390/su14159726

Vaidya, P., Greden, L., Eijadi, D., Mcdougall, T., & Cole, R. (2009). Integrated cost-estimation methodology to support high-performance building design. Energy Efficiency, 2(1), 69. https://doi.org/10.1007/s12053-008-9028-4

Valentini, O., Andreadou, N., Bertoldi, P., Lucas, A., Saviuc, I., & Kotsakis, E. (2022). Demand Response Impact Evaluation: A Review of Methods for Estimating the Customer Baseline Load. ENERGIES, 15(14). https://doi.org/10.3390/en15145259

van Reenen, C. A. (2020). Establishing a Heuristic Framework for Effective Attenuation of Traffic Noise Transmission in Typical Naturally Ventilated Classrooms in Urban Schools in Gauteng, South Africa. PQDT - Global. https://ezproxy.bu.edu/login?qurl=https://www.proquest.com/docview/2901488533?accountid=9676&bdid=77468&_bd=tqnm4S0sJBI5V9WKQzjBc00EDyw%3D

Vassiliades, C., Minterides, C., Astara, O., Barone, G., & Vardopoulos, I. (2023). Socio-Economic Barriers to Adopting Energy-Saving Bioclimatic Strategies in a Mediterranean Sustainable Real Estate Setting: A Quantitative Analysis of Resident Perspectives. ENERGIES, 16(24). https://doi.org/10.3390/en16247952

Velichko, E., & Tskhovrebov, E. (2018). Insights into the state of affairs and possible ways to improve secondary material waste management (WOS:000567656900202). 196. https://doi.org/10.1051/matecconf/201819604066

Veliz, K., Walters, J., Busco, C., & Vargas, M. (2023). Modeling barriers to a circular economy for construction demolition waste in the Aysen region of Chile. RESOURCES CONSERVATION & RECYCLING ADVANCES, 18. https://doi.org/10.1016/j.rcradv.2023.200145

Viness, T. (2016). A Case Study of Construction Challenges and Solutions: Installation of Exterior Insulation and Finish System (EIFS) to an Existing Residential Structure (WOS:000389160600013). 1585, 197–215. https://doi.org/10.1520/STP158520140113

Vivian, J., Chinello, M., Zarrella, A., & De Carli, M. (2022). Investigation on Individual and Collective PV Self-Consumption for a Fifth Generation District Heating Network. Energies, 15(3), 1022. https://doi.org/10.3390/en15031022

Vossos, V., Gerber, D., Gaillet-Tournier, M., Nordman, B., Brown, R., Heredia, W., Ghatpande, O., Saha, A., Arnold, G., & Frank, S. (2022). Adoption Pathways for DC Power Distribution in Buildings. ENERGIES, 15(3). https://doi.org/10.3390/en15030786

Wang, H., Pen-Chi Chiang, Cai, Y., Li, C., Wang, X., Tse-Lun, C., Wei, S., & Huang, Q. (2018). Application of Wall and Insulation Materials on Green Building: A Review. Sustainability, 10(9), 3331. https://doi.org/10.3390/su10093331

Wang, J., He, B., Wang, H., & Santamouris, M. (2019). Towards higher quality green building agenda—An overview of the application of green building techniques in China. SOLAR ENERGY, 193, 473–493. https://doi.org/10.1016/j.solener.2019.09.089

Wang, Q., Shen, C., Guo, Z., Zhu, K., Zhang, J., & Huang, M. (2023). Research on the Barriers and Strategies to Promote Prefabricated Buildings in China. BUILDINGS, 13(5). https://doi.org/10.3390/buildings13051200

Wang, S., & Chen, Q. (2024). Development of a Cloud-Based Building Information Modeling Design Configurator to Auto-Link Material Catalogs with Code-Compliant Designs of Residential Buildings. BUILDINGS, 14(7). https://doi.org/10.3390/buildings14072084

Wang, T., & Feng, J. (2022). Linking BIM Definition, BIM Capability Maturity, and Integrated Project Delivery in the AECO Industry: The Influences of BIM Diffusion and Moral Hazard. Journal of Urban Planning and Development, 148(3). https://doi.org/10.1061/(ASCE)UP.1943-5444.0000839

Wang, X., Teigland, R., & Hollberg, A. (2022). A Pathway to Climate Neutral Buildings: Definitions, Policy and Stakeholder Understanding in Sweden and China. IOP Conference Series. Earth and Environmental Science, 1078(1), 012122. https://doi.org/10.1088/1755-1315/1078/1/012122

Wang, X., Yuan, J., You, K., Ma, X., & Li, Z. (2023). Using Real Building Energy Use Data to Explain the Energy Performance Gap of Energy-Efficient Residential Buildings: A Case Study from the Hot Summer and Cold Winter Zone in China. Sustainability, 15(2), 1575. https://doi.org/10.3390/su15021575

Wang, Y. (2018). Overview of State Policies for Energy Efficiency in Buildings. Current Sustainable / Renewable Energy Reports, 5(1), 101–108. https://doi.org/10.1007/s40518-018-0100-1

Wang, Y., Chong, D., & Liu, X. (2021). Evaluating the Critical Barriers to Green Construction Technologies Adoption in China. SUSTAINABILITY, 13(12). https://doi.org/10.3390/su13126510

Waqar, A., Alharbi, L., Alotaibi, F., Alrasheed, K., Khan, A., & Almujibah, H. (2024). Challenges of Blockchain Implementation in Construction. JOURNAL OF ENGINEERING, 2024. https://doi.org/10.1155/2024/2442345

Waqar, A., Othman, I., Falqi, I., Almujibah, H., Alshehri, A., Alsulamy, S., & Benjeddou, O. (2023). Assessment of Barriers to Robotics Process Automation (RPA) Implementation in Safety Management of Tall Buildings. BUILDINGS, 13(7). https://doi.org/10.3390/buildings13071663

Waqar, A., Othman, I., Saad, N., Qureshi, A., Azab, M., & Khan, A. (2023). Complexities for adopting 3D laser scanners in the AEC industry: Structural equation modeling. APPLICATIONS IN ENGINEERING SCIENCE, 16. https://doi.org/10.1016/j.apples.2023.100160

Waqar, A., Qureshi, A., & Alaloul, W. (2023). Barriers to Building Information Modeling (BIM) Deployment in Small Construction Projects: Malaysian Construction Industry. SUSTAINABILITY, 15(3). https://doi.org/10.3390/su15032477

Waqar, A., Shafiq, N., Othman, I., Alsulamy, S., Alshehri, A., & Falqi, I. (2024). Deterrents to the IoT for smart buildings and infrastructure development: A partial least square modeling approach. HELIYON, 10(10). https://doi.org/10.1016/j.heliyon.2024.e31035

Wardani, S., Handayani, N., & Wibowo, M. (2022). Barriers for Implementing Reverse Logistics in the Construction Sectors. JOURNAL OF INDUSTRIAL ENGINEERING AND MANAGEMENT-JIEM, 15(3), 385–415. https://doi.org/10.3926/jiem.3539

Warren-Myers, G., Moosavi, S., Hurlimann, A., Raisbeck, P., Bush, J., March, A., & Browne, G. (2024). Barriers to and facilitators of climate change action in architecture practice. JOURNAL OF CLEANER PRODUCTION, 469. https://doi.org/10.1016/j.jclepro.2024.143149

Wasse, A., & Dai, K. (2024). Building Information Modeling Adoption, Implementation, and Challenges in the Ethiopian Construction Industry. JOURNAL OF ARCHITECTURAL ENGINEERING, 30(3). https://doi.org/10.1061/JAEIED.AEENG-1657

Weber, M., Pressburger, L., Chau, L., Khan, Z., Waite, T., Westphal, M., Ling, G., Ho, C., & Evans, M. (2024). Carbon neutrality in Malaysia and Kuala Lumpur: Insights from stakeholder-driven integrated assessment modeling. FRONTIERS IN ENERGY RESEARCH, 12. https://doi.org/10.3389/fenrg.2024.1336045

Weiss, J., Dunkelberg, E., & Vogelpohl, T. (2012). Improving policy instruments to better tap into homeowner refurbishment potential: Lessons learned from a case study in Germany. ENERGY POLICY, 44, 406–415. https://doi.org/10.1016/j.enpol.2012.02.006

Welege, N., Pan, W., & Kumaraswamy, M. (2023a). Engaging Stakeholders to Overcome the Common Constraints for Delivering Low Carbon Buildings in High-Rise High-Density Cities. JOURNAL OF CONSTRUCTION ENGINEERING AND MANAGEMENT, 149(1). https://doi.org/10.1061/JCEMD4.COENG-12327

Welege, N., Pan, W., & Kumaraswamy, M. (2023b). Stakeholder collaboration to mitigate constraints to delivering low-carbon buildings: Insights from high-rise high-density cities. ENGINEERING CONSTRUCTION AND ARCHITECTURAL MANAGEMENT. https://doi.org/10.1108/ECAM-02-2022-0166

Whitman, C., Prizeman, O., Walker, P., & Gwilliam, J. (2020). Heritage retrofit and cultural empathy; a discussion of challenges regarding the energy performance of historic UK timber-framed dwellings. INTERNATIONAL JOURNAL OF BUILDING PATHOLOGY AND ADAPTATION, 38(2), 386–404. https://doi.org/10.1108/IJBPA-02-2019-0023

Wiik, M. K. (2023). A comparative assessment of the development of GHG emission criteria and benchmark values for buildings in Norway. Journal of Physics: Conference Series, 2654(1), 012131. https://doi.org/10.1088/1742-6596/2654/1/012131

Wijesiri, W. M. M., Devapriya, K. A. K., Rathnasiri, P., & Tharindu Lakruwan Wickremanayake Karunaratne. (2022). A framework to implement green adaptive reuse for existing buildings in Sri Lanka. Intelligent Buildings International, 14(5), 581–605. https://doi.org/10.1080/17508975.2021.1906204

Willan, C., Janda, K. B., & Kenington, D. (2021). Seeking the Pressure Points: Catalysing Low Carbon Changes from the Middle-Out in Offices and Schools. Energies, 14(23), 8087. https://doi.org/10.3390/en14238087

Williams, J., Jones, R., & Torn, M. (2021). Observations on the transition to a net-zero energy system in the United States. ENERGY AND CLIMATE CHANGE, 2. https://doi.org/10.1016/j.egycc.2021.100050

Williams, M., Parker, C., Dillon, A., Billings, B., & Powell, K. (2024). Sustainability and affordability of building electrification: A state-by-state holistic approach for multifamily buildings. SUSTAINABLE CITIES AND SOCIETY, 109. https://doi.org/10.1016/j.scs.2024.105515

Wilson, E., Munankarmi, P., Less, B., Reyna, J., & Rothgeb, S. (2024). Heat pumps for all? Distributions of the costs and benefits of residential air-source heat pumps in the United States. JOULE, 8(4). https://doi.org/10.1016/j.joule.2024.01.022

Wimala, M., Akmalah, E., & Sururi, M. (2016). Breaking through the Barriers to Green Building Movement in Indonesia: Insights from Building Occupants (WOS:000390613000071). 100, 469–474. https://doi.org/10.1016/j.egypro.2016.10.204

Wise, F., Moncaster, A., & Jones, D. (2021). Rethinking retrofit of residential heritage buildings. Buildings & Cities, 2(1), 495. https://doi.org/10.5334/bc.94

Wolf, A. (2022). Lumber Lowdown. *Builder*, *45*(3), 20.

Wong, F., Oldfield, P., & Osmond, P. (2024). Industry perceptions of mass engineered timber (MET) construction: A comparison of South-East Asia with other regions. ARCHITECTURAL ENGINEERING AND DESIGN MANAGEMENT. https://doi.org/10.1080/17452007.2024.2401561

Wróblewski, P., & Niekurzak, M. (2022). Assessment of the Possibility of Using Various Types of Renewable Energy Sources Installations in Single-Family Buildings as Part of Saving Final Energy Consumption in Polish Conditions. Energies, 15(4), 1329. https://doi.org/10.3390/en15041329

Wu, P., Jin, R., Xu, Y., Lin, F., Dong, Y., & Pan, Z. (2021). THE ANALYSIS OF BARRIERS TO BIM IMPLEMENTATION FOR INDUSTRIALIZED BUILDING CONSTRUCTION: A CHINA STUDY. JOURNAL OF CIVIL ENGINEERING AND MANAGEMENT, 27(1), 1–13. https://doi.org/10.3846/jcem.2021.14105
Wu, Y., Wu, Y., Cimen, H., Vasquez, J., & Guerrero, J. (2022). Towards collective energy Community: Potential roles of microgrid and blockchain to go beyond P2P energy trading. APPLIED ENERGY, 314. https://doi.org/10.1016/j.apenergy.2022.119003
Wu, Z., Jiang, M., Cai, Y., Wang, H., & Li, S. (2019). What Hinders the Development of Green Building? An Investigation of China. INTERNATIONAL JOURNAL OF ENVIRONMENTAL RESEARCH AND PUBLIC HEALTH, 16(17). https://doi.org/10.3390/ijerph16173140
Wuni, I., Abankwa, D., Koc, K., Adukpo, S., & Antwi-Afari, M. (2024). Critical barriers to the adoption of integrated digital delivery in the construction industry. JOURNAL OF BUILDING ENGINEERING, 83. https://doi.org/10.1016/j.jobe.2024.108474
Xia, X. M., & Wang, F. (2020). Economic Feasibility and Comprehensive Benefit Evaluation of Rural Household Biogas Utilization: Evidence from China. IOP Conference Series. Earth and Environmental Science, 510(3). https://doi.org/10.1088/1755-1315/510/3/032034
Xia-Bauer, C., Gokarakonda, S., Guo, S., Filippidou, F., Thomas, S., Maheshwari, J., & Vishwanathan, S. (2024). Comparative analysis of residential building decarbonization policies in major economies: Insights from the EU, China, and India. ENERGY EFFICIENCY, 17(5). https://doi.org/10.1007/s12053-024-10225-w
Xian, S., & Chen, H. (2015). Revitalisation of industrial buildings in Hong Kong: New measures, new constraints? HABITAT INTERNATIONAL, 47, 298–306. https://doi.org/10.1016/j.habitatint.2015.02.004
Xiao, H., Lai, W., Chen, A., Lai, S., He, W., Deng, X., Zhang, C., & Ren, H. (2024). Application of Photovoltaic and Solar Thermal Technologies in Buildings: A Mini-Review. Coatings, 14(3), 257. https://doi.org/10.3390/coatings14030257
Xing, R., Hanaoka, T., Kanamori, Y., & Masui, T. (2018). Achieving zero emission in China's urban building sector: Opportunities and barriers. Current Opinion in Environmental Sustainability, 30, 115–122. https://doi.org/10.1016/j.cosust.2018.05.005
Xu, L., Cherian, J., Zaheer, M., Muhammad Safdar Sial, Comite, U., Cismas, L. M., Juan Felipe Espinosa Cristia, & Oláh, J. (2022). The Role of Healthcare Employees' Pro-Environmental Behavior for De-Carbonization: An Energy Conservation Approach from CSR Perspective. Energies, 15(9), 3429. https://doi.org/10.3390/en15093429
Xu, P., Chan, E., & Lam, P. (2009). *A Framework for Energy Efficiency Retrofits of Existing Buildings (EEREB) in China* (WOS:000281119900076). 501–507.
Xu, Y., Chong, H., & Chi, M. (2023). Modelling the blockchain adoption barriers in the AEC industry. ENGINEERING CONSTRUCTION AND ARCHITECTURAL MANAGEMENT, 30(1), 125–153. https://doi.org/10.1108/ECAM-04-2021-0335
Xu, Y., Zhang Ruijie, Fan Xiaomin, & Wang Qiutong. (2022). How does green technology innovation affect urbanization? An empirical study from provinces of China. Environmental Science and Pollution Research, 29(24), 36626–36639. https://doi.org/10.1007/s11356-021-18117-7
Xu, Z., Li, X., Ma, L., Lu, Y., & Liu, G. (2024). Unraveling the Knowledge Roadmap of Building Policy Mixes: A Scientometric Analysis. SUSTAINABILITY, 16(1). https://doi.org/10.3390/su16010428
Yan, R., Xiang, X., Cai, W., & Ma, M. (2022). Decarbonizing residential buildings in the developing world: Historical cases from China. SCIENCE OF THE TOTAL ENVIRONMENT, 847. https://doi.org/10.1016/j.scitotenv.2022.157679
Yang, H., Gao, W., Wei, X., Wang, Y., & Li, Y. (2024). Techno-economic comparative analysis of PV third–party ownership between customer and developer sides in Japan. Journal of Energy Storage, 80, 110062. https://doi.org/10.1016/j.est.2023.110062
Yang, P. (2022). Urban expansion of Energiewende in Germany: A systematic bibliometric analysis and literature study. ENERGY SUSTAINABILITY AND SOCIETY, 12(1). https://doi.org/10.1186/s13705-022-00373-1
Yang, Y., Chew, B., Loo, H., & Tan, L. (2017). Green Commercial Building Insurance in Malaysia (WOS:000405771500070). 1818. https://doi.org/10.1063/1.4976935
Yasser Yahya Al-Ashmori, Idris Bin Othman, Hisham Bin Mohamad, Rahmawati, Y., & Napiah, M. (2019). Establishing the Level of BIM implementation – A Case Study in Melaka, Malaysia. IOP Conference Series. Materials Science and Engineering, 601(1). https://doi.org/10.1088/1757-899X/601/1/012024
Ye, Y., Dehwah, A., Faulkner, C., Sathyanarayanan, H., & Lei, X. (2023). A Perspective of Decarbonization Pathways in Future Buildings in the United States. BUILDINGS, 13(4). https://doi.org/10.3390/buildings13041003

Yeoh, J., Lee, S., & Ong, K. (2018). *Development of a BIM-Based Framework to Support the Analysis of "Design-for-Disassembly" Strategies* (WOS:000541103300075). 759–768.

Yeung, H., Ridwan, T., Tariq, S., & Zayed, T. (2022). BEAM Plus implementation in Hong Kong: Assessment of challenges and policies. INTERNATIONAL JOURNAL OF CONSTRUCTION MANAGEMENT, 22(14), 2830–2844. https://doi.org/10.1080/15623599.2020.1827692

Yin, B., Laing, R., Leon, M., & Mabon, L. (2018). An evaluation of sustainable construction perceptions and practices in Singapore. SUSTAINABLE CITIES AND SOCIETY, 39, 613–620. https://doi.org/10.1016/j.scs.2018.03.024

York, J., Vedula, S., & Lenox, M. (2018). IT'S NOT EASY BUILDING GREEN: THE IMPACT OF PUBLIC POLICY, PRIVATE ACTORS, AND REGIONAL LOGICS ON VOLUNTARY STANDARDS ADOPTION. ACADEMY OF MANAGEMENT JOURNAL, 61(4), 1492–1523. https://doi.org/10.5465/amj.2015.0769

Young, R. A. (2012). Stewardship of the Built Environment; Sustainability, Preservation, and Reuse. Stewardship of the Built Environment. https://ezproxy.bu.edu/login?qurl=https://www.proquest.com/docview/2131850619?accountid=9676&bdid=77468&_bd=07D5kcoJjDqtehmYWHkA%2FrJ6eDU%3D

Youssef, E., Zaid, R., & Mohamed, E. K. (2022). Economic barriers to green innovations in Morocco: The case of a zero-energy house. IOP Conference Series. Earth and Environmental Science, 975(1), 012005. https://doi.org/10.1088/1755-1315/975/1/012005

Yu, M. G., PhD, Ma, X., PhD, Huang, B., PhD, Devaprasad, K., Brown, F., PhD, & Wu, D., PhD. (2024). Enhancing Building Energy Efficiency through Advanced Sizing and Dispatch Methods for Energy Storage. *ASHRAE Transactions*, *130*, 512–519.

Yuan, H. (2017). Barriers and countermeasures for managing construction and demolition waste: A case of Shenzhen in China. JOURNAL OF CLEANER PRODUCTION, 157, 84–93. https://doi.org/10.1016/j.jclepro.2017.04.137

Yuan, Z., Ni, G., Wang, L., Qiao, Y., Sun, C., Xu, N., & Wang, W. (2020). Research on the Barrier Analysis and Strength Measurement of a Prefabricated Building Design. SUSTAINABILITY, 12(7). https://doi.org/10.3390/su12072994

Zachariadis, T., Michopoulos, A., Vougiouklakis, Y., Piripitsi, K., Ellinopoulos, C., & Struss, B. (2018). Determination of Cost-Effective Energy Efficiency Measures in Buildings with the Aid of Multiple Indices. Energies, 11(1), 191. https://doi.org/10.3390/en11010191

Zalina Shari, & Soebarto, V. (2014). Investigating sustainable practices in the Malaysian office building developments. Construction Innovation, 14(1), 17–37. https://doi.org/10.1108/CI-12-2012-0064

Zaman, A., Chan, Y., Jonescu, E., & Stewart, I. (2022). Critical Challenges and Potential for Widespread Adoption of Mass Timber Construction in Australia-An Analysis of Industry Perceptions. BUILDINGS, 12(9). https://doi.org/10.3390/buildings12091405

Zambrano-Prado, P., Orsini, F., Rieradevall, J., Josa, A., & Gabarrell, X. (2021). Potential Key Factors, Policies, and Barriers for Rooftop Agriculture in EU Cities: Barcelona, Berlin, Bologna, and Paris. FRONTIERS IN SUSTAINABLE FOOD SYSTEMS, 5. https://doi.org/10.3389/fsufs.2021.733040

Zapata-Lancaster, G. (2014). Low carbon non-domestic building design process. An ethnographic comparison of design in Wales and England. Structural Survey, 32(2), 140. https://doi.org/10.1108/SS-07-2013-0029

Zapata-Lancaster, G., & Tweed, C. (2014). Designers' enactment of the policy intentions. An ethnographic study of the adoption of energy regulations in England and Wales. ENERGY POLICY, 72, 129–139. https://doi.org/10.1016/j.enpol.2014.04.033

Železná, J., Felicioni, L., Trubina, N., Vlasatá, B., Růžička, J., & Veselka, J. (2024). Whole Life Carbon Assessment of Buildings: The Process to Define Czech National Benchmarks. Buildings, 14(7), 1936. https://doi.org/10.3390/buildings14071936

Zeno, B., Schnieders Jürgen, Conner, W., Kaufmann Berthold, Lepp Laszlo, Norwood Zack, Simmonds, A., & Theoboldt Ingo. (2022). Retrofit with Passive House components. Energy Efficiency, 15(1). https://doi.org/10.1007/s12053-021-10008-7

Zhang, F., Deng, H., Margolis, R., & Su, J. (2015). Analysis of distributed-generation photovoltaic deployment, installation time and cost, market barriers, and policies in China. *Energy Policy*, *81*, 43–43.

Zhang, G., & Setunge, S. (2009). *Critical Factors Influencing Building Service Upgrade in Heritage Property* (WOS:000281119901091). 2049–2056.

Zhang, H., Hewage, K., Karunathilake, H., Feng, H., & Sadiq, R. (2021). Research on policy strategies for implementing energy retrofits in the residential buildings. JOURNAL OF BUILDING ENGINEERING, 43. https://doi.org/10.1016/j.jobe.2021.103161

Zhang, L., & Zhou, J. (2015). Drivers and barriers of developing low-carbon buildings in China: Real estate developers’ perspectives. *International Journal of Environmental Technology and Management*, *18*(3), 254.

Zhang, L., Song, G., Ma, X., Zhan, C., & Zhang, S. (2020). Decarbonising residential building energy towards achieving the intended nationally determined contribution at subnational level under uncertainties. JOURNAL OF CLEANER PRODUCTION, 272. https://doi.org/10.1016/j.jclepro.2020.122760

Zhang, N., Hwang, B., Deng, X., & Tay, F. (2020). Collaborative contracting in the Singapore construction industry: Current status, major barriers and best solutions. ENGINEERING CONSTRUCTION AND ARCHITECTURAL MANAGEMENT, 27(10), 3115–3133. https://doi.org/10.1108/ECAM-08-2019-0451

Zhang, S., Xiang, X., Ma, Z., Ma, M., & Zou, C. (2021). Carbon Neutral Roadmap of Commercial Building Operations by Mid-Century: Lessons from China. BUILDINGS, 11(11). https://doi.org/10.3390/buildings11110510

Zhang, Y., Wang, H., Gao, W., Wang, F., Zhou, N., Kammen, D. M., & Ying, X. (2019). A Survey of the Status and Challenges of Green Building Development in Various Countries. Sustainability, 11(19), 5385. https://doi.org/10.3390/su11195385

Zhao, L., Zhang, L., Sun, J., & He, P. (2022). Can public participation constraints promote green technological innovation of Chinese enterprises? The moderating role of government environmental regulatory enforcement. TECHNOLOGICAL FORECASTING AND SOCIAL CHANGE, 174. https://doi.org/10.1016/j.techfore.2021.121198

Zhao, X., Hwang, B., & Lu, Q. (2018). Typology of business model innovations for delivering zero carbon buildings. JOURNAL OF CLEANER PRODUCTION, 196, 1213–1226. https://doi.org/10.1016/j.jclepro.2018.06.018

Zheng, M., & Jørgensen, B. N. (2018). A discussion of building automation and stakeholder engagement for the readiness of energy flexible buildings. Energy Informatics, 1(1). https://doi.org/10.1186/s42162-018-0061-z

Zheng, X., Sun, C., & Liu, J. (2024). Exploring stakeholder engagement in urban village renovation projects through a mixed-method approach to social network analysis: A case study of Tianjin. HUMANITIES & SOCIAL SCIENCES COMMUNICATIONS, 11(1). https://doi.org/10.1057/s41599-023-02536-7

Zhou, Y., & Herr, C. M. (2023). A Review of Advanced Façade System Technologies to Support Net-Zero Carbon High-Rise Building Design in Subtropical China. Sustainability, 15(4), 2913. https://doi.org/10.3390/su15042913

Zilberova, I., Mailyan, V., & Zilberov, R. (2023). Organization of major repairs of apartment buildings with energy-saving technologies. E3S Web of Conferences, 376, n/a. https://doi.org/10.1051/e3sconf/202337603022

zu Ermgassen, S., Drewniok, M., Bull, J., Walker, C., Mancini, M., Ryan-Collins, J., & Serrenho, A. (2022). A home for all within planetary boundaries: Pathways for meeting England?s housing needs without transgressing national climate and biodiversity goals. ECOLOGICAL ECONOMICS, 201. https://doi.org/10.1016/j.ecolecon.2022.107562

Zuberi, M., Chambers, J., & Patel, M. (2021). Techno-economic comparison of technology options for deep decarbonization and electrification of residential heating. ENERGY EFFICIENCY, 14(7). https://doi.org/10.1007/s12053-021-09984-7

Zuberi, M., Narula, K., Klinke, S., Chambers, J., Streicher, K., & Patel, M. (2021). Potential and costs of decentralized heat pumps and thermal networks in Swiss residential areas. INTERNATIONAL JOURNAL OF ENERGY RESEARCH, 45(10), 15245–15264. https://doi.org/10.1002/er.6801

Zulu, S., Zulu, E., Chabala, M., & Chunda, N. (2023). Drivers and barriers to sustainability practices in the Zambian Construction Industry. INTERNATIONAL JOURNAL OF CONSTRUCTION MANAGEMENT, 23(12), 2116–2125. https://doi.org/10.1080/15623599.2022.2045425

Zwickl-Bernhard, S., Auer, H., & Golab, A. (2022). Equitable decarbonization of heat supply in residential multi-apartment rental buildings: Optimal subsidy allocation between the property owner and tenants. ENERGY AND BUILDINGS, 262. https://doi.org/10.1016/j.enbuild.2022.112013

Supplementary References: Legislative Sources

Legislative Source Title	Published date	Publication

House Transportation and Infrastructure Subcommittee on Economic Development, Public Buildings, and Emergency Management Holds Hearing on Federal Protective Service Effectiveness	July 23, 2024 Tuesday	CQ Transcriptions
House Agriculture Subcommittee on Livestock and Foreign Agriculture Holds Hearing on Beef Supply	July 28, 2021 Wednesday	CQ Transcriptions
House Natural Resources Subcommittee on Indigenous Peoples of the United States Holds Hearing on Federal Funding Shortfalls in Indian Country	November 19, 2019 Tuesday	CQ Transcriptions
Senate Homeland Security and Governmental Affairs Committee Holds Hearing on Pending Nominations	July 25, 2024 Thursday	CQ Transcriptions
House Financial Services Committee Holds Hearing on Monetary Policy and State of the Economy	July 14, 2021 Wednesday	CQ Transcriptions
House Financial Services Subcommittee on Housing, Community Development, and Insurance Holds Hearing on Freedmen in Native American Communities	July 27, 2021 Tuesday	CQ Transcriptions
House Financial Services Committee Holds Hearing on Affordable Housing Crisis and Gentrification	January 14, 2020 Tuesday	CQ Transcriptions
Senate Banking, Housing and Urban Affairs Committee Holds Hearing on Pending Nominations	July 13, 2021 Tuesday	CQ Transcriptions
House Transportation and Infrastructure Subcommittee on Highways and Transit Holds Hearing on Transportation's Regulatory Administration	July 24, 2024 Wednesday	CQ Transcriptions
House Appropriations Subcommittee on Military Construction, Veterans Affairs and Related Agencies Holds Hearing on the Fiscal Year 2025 Navy and Marine Corps Military Construction Budget Request	April 11, 2024 Thursday	CQ Transcriptions
House Transportation and Infrastructure Subcommittee on Coast Guard and Maritime Transportation Holds Hearing on Commercial and Passenger Vessel Safety	November 14, 2019 Thursday	CQ Transcriptions
House Appropriations Subcommittee on Homeland Security Holds Hearing on Fiscal Year 2023 Department of Homeland Security Budget	April 27, 2022 Wednesday	CQ Transcriptions
Senate Environment and Public Works Committee Holds Hearing on USACE Water Infrastructure Implementation	January 12, 2022 Wednesday	CQ Transcriptions
House Appropriations Subcommittee on Homeland Security Holds Hearing on Fiscal Year 2024 Budget Request for the Department of Homeland Security	March 29, 2023 Wednesday	CQ Transcriptions
House Appropriations Subcommittee on Commerce, Justice and Science Holds Hearing on Executive Office for Immigration Review	March 7, 2019 Thursday	CQ Transcriptions
House Armed Services Subcommittee on Strategic Forces Holds Hearing on the Fiscal Year 2025 Nuclear Forces and Atomic Energy Defense Budget Request	April 30, 2024 Tuesday	CQ Transcriptions
House Appropriations Subcommittee on Transportation, and Housing and Urban Development and Related Agencies Holds Hearing on Fiscal Year 2025 Department of Housing and Urban Development Budget Request	May 1, 2024 Wednesday	CQ Transcriptions
House Transportation and Infrastructure Subcommittee on Coast Guard and Maritime Transportation Holds Hearing on Fiscal Year 2023 Coast Guard and Maritime Transportation Programs Budget	April 27, 2022 Wednesday	CQ Transcriptions
House Small Business Committee Holds Hearing on SBA Small Business Size Standards	February 6, 2024 Tuesday	CQ Transcriptions
House Veterans' Affairs Committee Holds Hearing on VA Infrastructure	May 27, 2021 Thursday	CQ Transcriptions

Senate Foreign Relations Subcommittee on State Department and USAID Management, International Operations and Bilateral International Development Holds Hearing on USAID Localization	March 9, 2023 Thursday	CQ Transcriptions
Senate Environment and Public Works Committee Holds Hearing on Pending Legislation	September 7, 2022 Wednesday	CQ Transcriptions
House Transportation and Infrastructure Subcommittee on Coast Guard and Maritime Transportation Holds Hearing on Coast Guard Manpower Shortages	March 6, 2024 Wednesday	CQ Transcriptions
House Energy and Commerce Subcommittee on Environment and Climate Change Holds Hearing on the New Coronavirus and Environmental Justice	June 9, 2020 Tuesday	CQ Transcriptions
House Education and Labor Committee Holds Hearing on Public Education and the New Coronavirus	June 15, 2020 Monday	CQ Transcriptions
Senate Armed Services Committee Holds Hearing on the Fiscal Year 2024 Department of Energy and National Nuclear Security Administration Atomic Energy Defense Activities Defense Authorization Request	April 26, 2023 Wednesday	CQ Transcriptions
House Science, Space and Technology Committee Holds Hearing on National Institute of Standards and Technology Priorities	May 22, 2024 Wednesday	CQ Transcriptions
House Appropriations Subcommittee on Energy and Water Development Holds Hearing on Members Priorities	April 9, 2019 Tuesday	CQ Transcriptions
House Appropriations Subcommittee on Energy and Water Development Holds Hearing on Members Priorities	April 9, 2019 Tuesday	CQ Transcriptions
House Education and the Workforce Committee Holds Hearing on Labor Department Policies and Priorities	May 1, 2024 Wednesday	CQ Transcriptions
U.S.-China Economic and Security Review Commission Holds Hearing on Issues Surrounding China	February 7, 2019 Thursday	CQ Transcriptions
Senate Environment and Public Works Subcommittee on Clean Air, Climate and Nuclear Safety Holds Hearing on Pending Nominations	April 6, 2022 Wednesday	CQ Transcriptions
Senate Appropriations Subcommittee on Homeland Security Holds Hearing on the Fiscal Year 2023 Homeland Security Budget Request	May 4, 2022 Wednesday	CQ Transcriptions
House Homeland Security Committee Holds Hearing on the Way Forward on Homeland Security	March 17, 2021 Wednesday	CQ Transcriptions
House Ways and Means Committee Holds Hearing on Tax Code and Infrastructure Investment	May 19, 2021 Wednesday	CQ Transcriptions
Bipartisan Policy Center Holds Webinar on Voting	December 15, 2020 Tuesday	CQ Transcriptions
Senate Banking, Housing and Urban Affairs Committee Holds Hearing on Investing in Rural Communities	April 20, 2021 Tuesday	CQ Transcriptions
House Transportation and Infrastructure Committee Holds Hearing on Department of Transportation's Policies and Programs	September 20, 2023 Wednesday	CQ Transcriptions
House Education and Labor Committee Holds Hearing on Labor Department Policies and Priorities	June 14, 2022 Tuesday	CQ Transcriptions

President Joe Biden Delivers Remarks at Bipartisan Infrastructure Deal Signing Ceremony	November 15, 2021 Monday	CQ Transcriptions
Senate Rules and Administration Committee Holds Hearing on General Election Preparations	July 22, 2020 Wednesday	CQ Transcriptions
House Armed Services Subcommittees on Readiness and Seapower and Projection Forces Hold Joint Hearing on Sealift and Mobility Requirements and the National Defense Strategy	March 11, 2020 Wednesday	CQ Transcriptions
House Judiciary Committee Holds Hearing on Homeland Security Oversight	April 28, 2022 Thursday	CQ Transcriptions
Senate Foreign Relations Committee Holds Hearing on Pending Nominations	October 20, 2021 Wednesday	CQ Transcriptions
House Financial Services Committee Holds Hearing on Housing Finance Policy	October 22, 2019 Tuesday	CQ Transcriptions
Senate Health, Education, Labor and Pensions Committee Holds Hearing on the Nomination of Julie Su to be Deputy Labor Secretary	March 16, 2021 Tuesday	CQ Transcriptions
House Judiciary Subcommittee on Antitrust, Commercial and Administrative Law Holds Hearing on Online Platforms and Market Power	July 16, 2019 Tuesday	CQ Transcriptions
House Foreign Affairs Subcommittee on Europe, Eurasia, Energy and the Environment Holds Hearing on U.S.-Europe Alliance	March 26, 2019 Tuesday	CQ Transcriptions
House Armed Services Committee Holds Hearing on Fiscal Year 2025 Department of the Army Budget Request	April 16, 2024 Tuesday	CQ Transcriptions
Senate Homeland Security and Governmental Affairs Committee Holds Hearing on the Nomination of Alejandro Mayorkas to be Homeland Security Secretary	January 19, 2021 Tuesday	CQ Transcriptions
Senate Armed Services Subcommittee on Emerging Threats and Capabilities Holds Hearing on Defense Innovation	April 6, 2022 Wednesday	CQ Transcriptions
CNN "STATE OF THE UNION"	July 30, 2023 Sunday	CQ Transcriptions
Middle East Policy Council Holds Briefing on Future of U.S. Engagement in the Middle East	April 12, 2019 Friday	CQ Transcriptions
American Constitution Society Holds News Teleconference on Institutional Racism and the Legal System	June 5, 2020 Friday	CQ Transcriptions
House Oversight And Reform Committee Holds Roundtable on Environmental Justice	July 21, 2021 Wednesday	CQ Transcriptions
Senate Finance Committee Holds Hearing on the Tax Code and Domestic Manufacturing	March 16, 2021 Tuesday	CQ Transcriptions
House Select Economic Disparity and Fairness in Growth Committee Holds Hearing on Family Caregiving and Economic Growth	December 8, 2021 Wednesday	CQ Transcriptions
Senate Foreign Relations Committee Holds Hearing on the Nomination of John Sullivan to be Ambassador to Russia	October 30, 2019 Wednesday	CQ Transcriptions

House Appropriations Subcommittee on Financial Services and General Government Holds Hearing on Universal Broadband	May 18, 2021 Tuesday	CQ Transcriptions
House Transportation and Infrastructure Subcommittee on Aviation Holds Hearing on Future of Aviation	March 12, 2019 Tuesday	CQ Transcriptions
Senate Homeland Security and Governmental Affairs Committee Holds Hearing on Pending Nominations	March 4, 2021 Thursday	CQ Transcriptions
Senate Armed Services Holds Hearing on Pending Nominations	April 30, 2019 Tuesday	CQ Transcriptions
Senate Judiciary Committee Holds Hearing on Voting Rights	April 20, 2021 Tuesday	CQ Transcriptions
Senate Veterans' Affairs Committee Holds Hearing on Pending Nominations	May 19, 2021 Wednesday	CQ Transcriptions
House Judiciary Subcommittee on The Constitution, Civil Rights and Civil Liberties Holds Hearing on Litigation Reform and Qualified Immunity, Part One	March 31, 2022 Thursday	CQ Transcriptions
Senate Health, Education, Labor, and Pensions Committee Holds Hearing on Labor and the Right to Organize	July 22, 2021 Thursday	CQ Transcriptions
US-China Economic and Security Review Commission Holds Hearing on US -China Relations	September 4, 2019 Wednesday	CQ Transcriptions
Senate Armed Services Committee Holds Hearing on Pending Nominations	February 17, 2022 Thursday	CQ Transcriptions
House Armed Services Subcommittee on Military Personnel Holds Hearing on Transgender Military Service Policy	February 27, 2019 Wednesday	CQ Transcriptions
House Appropriations Subcommittee on Energy and Water Development Holds Hearing on Fiscal 2020 Budget Request for the Energy Department and National Nuclear Security Administration	April 2, 2019 Tuesday	CQ Transcriptions
US-China Economic and Security Review Commission Holds Meeting on China's Nuclear Forces	June 10, 2021 Thursday	CQ Transcriptions
Senate Armed Services Subcommittee on Personnel Holds Hearing on Military Personnel Policies and Family Readiness	February 27, 2019 Wednesday	CQ Transcriptions
House Appropriations Subcommittee on Agriculture, Rural Development and Food and Drug Administration Holds Hearing on Economic Research and National Institute of Food-Agriculture Relocation	March 27, 2019 Wednesday	CQ Transcriptions
House Appropriations Subcommittee on Interior and Environment Holds Hearing on Non-Tribal Programs - Public Witnesses, Morning Session	February 6, 2020 Thursday	CQ Transcriptions
Senate Appropriations Subcommittee on Transportation, Housing and Urban Development Holds Hearing on Fiscal 2022 Budget Request for the Transportation Department	June 16, 2021 Wednesday	CQ Transcriptions
House Financial Services Committee Holds Hearing on Consumer Financial Protection Bureau Review	March 7, 2019 Thursday	CQ Transcriptions
House Appropriations Subcommittee on Commerce, Justice, Science and Related Agencies Holds Hearing on Fiscal Year 2023 National Science Foundation Budget Request	May 11, 2022 Wednesday	CQ Transcriptions

House Armed Services Committee Holds Hearing on the Fiscal Year 2024 Department of the Army Budget Request	April 19, 2023 Wednesday	CQ Transcriptions
House Transportation and Infrastructure Subcommittee on Coast Guard and Maritime Transportation Holds Hearing on US-Flag Fleet and Shipbuilding Industry	July 9, 2024 Tuesday	CQ Transcriptions
House Appropriations Subcommittee on Labor, Health and Human Services and Education Holds Hearing on Fiscal 2022 Budget Request for the Labor Department	April 28, 2021 Wednesday	CQ Transcriptions
House Energy and Commerce Subcommittee on Innovation, Data and Commerce Holds Hearing on Safeguarding Data and Innovation	October 18, 2023 Wednesday	CQ Transcriptions
House Appropriations Subcommittee on Labor, Health and Human Services and Education Holds Hearing on Unaccompanied Children Program Oversight	July 24, 2019 Wednesday	CQ Transcriptions
House Oversight and Reform Subcommittee on Environment Holds Hearing on PFAS Contamination and Corporate Accountability	September 10, 2019 Tuesday	CQ Transcriptions
Senate Energy and Natural Resources Committee Holds Hearing on Fiscal 2020 Budget Request for the Energy Department	April 2, 2019 Tuesday	CQ Transcriptions
House Homeland Security Committee Holds Hearing on Elections	August 28, 2020 Friday	CQ Transcriptions
House Armed Services Subcommittee on Readiness Holds Hearing on Modernizing Military Depots	October 28, 2021 Thursday	CQ Transcriptions
House Transportation and Infrastructure Subcommittee on Railroads, Pipelines, and Hazardous Materials Holds Hearing on the Surface Transportation Board and Passenger Rail s	November 18, 2020 Wednesday	CQ Transcriptions
House Appropriations Subcommittee on Interior, Environment and Related Agencies Holds Hearing on Agency Spending Restrictions During Government Shutdown	February 6, 2019 Wednesday	CQ Transcriptions
House Foreign Affairs Committee Holds Hearing on the NATO Alliance	March 13, 2019 Wednesday	CQ Transcriptions
Senate Judiciary Committee Holds Hearing on the Nomination of Ketanji Brown Jackson to be an Associate Justice on the Supreme Court of the United States , Day 4	March 24, 2022 Thursday	CQ Transcriptions
Senate Appropriations Subcommittee on Interior and Environment Holds Hearing on Fiscal 2020 Budget Request for Indian Health Service	May 1, 2019 Wednesday	CQ Transcriptions
Senate Appropriations Subcommittee on Interior and Environment Holds Hearing on Fiscal 2020 Budget Request for Indian Health Service	May 1, 2019 Wednesday	CQ Transcriptions
House Ways and Means Committee Holds Hearing on Legislative Proposals for Paid Family and Medical Leave	January 28, 2020 Tuesday	CQ Transcriptions
House Appropriations Subcommittee on Commerce, Justice and Science Holds Hearing on Climate Change Research	February 26, 2019 Tuesday	CQ Transcriptions
House Education and Labor Committee Holds Hearing on HHS Policies and Priorities	June 16, 2021 Wednesday	CQ Transcriptions
Senate Armed Services Committee Holds Hearing on The Nomination of Mark Esper to be Defense Secretary	July 16, 2019 Tuesday	CQ Transcriptions

House Financial Services Subcommittee on Investor Protection, Entrepreneurship and Capital Markets Holds Hearing on Bond Rating Agencies	July 21, 2021 Wednesday	CQ Transcriptions
Senate Judiciary Committee Holds Hearing on Pending Nominations	December 1, 2021 Wednesday	CQ Transcriptions
House Appropriations Subcommittee on Transportation and Housing and Urban Development Holds Hearing on Fiscal 2021 Budget Request for the Department of Transportation	February 27, 2020 Thursday	CQ Transcriptions
House Appropriations Committee Holds Markup on Fiscal 2022 Homeland Security and Defense Appropriations	July 13, 2021 Tuesday	CQ Transcriptions
Atlantic Council Holds Discussion on US Interests in Ukraine , Part One	December 4, 2019 Wednesday	CQ Transcriptions
House Transportation and Infrastructure Subcommittee on Highways and Transit Holds Hearing on Automated Commercial Motor Vehicles	September 13, 2023 Wednesday	CQ Transcriptions
House Financial Services Subcommittee on Oversight and Investigations Holds Hearing on CDBF Disaster Recovery	July 15, 2021 Thursday	CQ Transcriptions
House Armed Services Committee Holds Markup on the National Defense Authorization Act for Fiscal 2024, Part I	June 21, 2023 Wednesday	CQ Transcriptions
House Armed Services Committee Holds Hearing on Fiscal 2020 Defense Authorization	March 26, 2019 Tuesday	CQ Transcriptions
Senate Indian Affairs Holds Hearing on the 1866 Reconstruction Treaties	July 27, 2022 Wednesday	CQ Transcriptions
Senate Judiciary Committee Holds Hearing on Pending Nominations	June 9, 2021 Wednesday	CQ Transcriptions
Senate Armed Services Subcommittee on Seapower Holds Hearing on Navy and Marine Corps Investment Programs	June 8, 2021 Tuesday	CQ Transcriptions
MSNBC Hosts 2019 Democratic Presidential Candidates Debate in Atlanta	November 20, 2019 Wednesday	CQ Transcriptions
Senate Commerce, Science and Transportation Subcommittee on Security Holds Hearing on the Coast Guard and National Interests in the Arctic	December 8, 2020 Tuesday	CQ Transcriptions
Senate Health, Education, Labor and Pensions Committee Holds Hearing on Pending Nominations	September 13, 2022 Tuesday	CQ Transcriptions
House Judiciary Committee Holds Hearing on Justice Department Oversight	July 28, 2020 Tuesday	CQ Transcriptions
Senate Appropriations Subcommittee on Financial Services and General Government Holds Hearing on Fiscal 2022 Budget Request for the Treasury Department	June 23, 2021 Wednesday	CQ Transcriptions
House Oversight and Reform Subcommittee on National Security Holds Hearing on Pathway for Peace in Afghanistan	February 19, 2021 Friday	CQ Transcriptions

Senate Armed Services Committee Holds Hearing on U.S. Strategic Command and U.S. Northern Command	February 26, 2019 Tuesday	CQ Transcriptions
House Judiciary Subcommittee on Antitrust, Commercial and Administrative Law Holds Hearing on Impacts of T-Mobile / Sprint Merger	March 12, 2019 Tuesday	CQ Transcriptions
House Homeland Security Subcommittee on Border Security, Facilitation and Operations Holds Hearing on ICE Contractors' New Coronavirus Response	July 13, 2020 Monday	CQ Transcriptions
House Appropriations Subcommittee on Homeland Security Holds Hearing on the Fiscal Year 2023 Coast Guard Budget Request	May 12, 2022 Thursday	CQ Transcriptions
House Foreign Affairs Subcommittee on Middle East, North Africa and International Terrorism Holds Hearing on Idlib , Syria	March 11, 2020 Wednesday	CQ Transcriptions
Senate Judiciary Subcommittee on Competition Policy, Antitrust and Consumer Rights Holds Hearing on Examining Competition in App Stores	April 21, 2021 Wednesday	CQ Transcriptions
House Armed Services Committee Holds Markup on Fiscal 2020 National Defense Authorization Act	June 12, 2019 Wednesday	CQ Transcriptions
House Armed Services Committee Holds Markup on Fiscal 2020 National Defense Authorization Act	June 12, 2019 Wednesday	CQ Transcriptions
House Armed Services Committee Holds Markup on Fiscal 2020 National Defense Authorization Act	June 12, 2019 Wednesday	CQ Transcriptions
House Armed Services Committee Holds Markup on Fiscal 2020 National Defense Authorization Act	June 12, 2019 Wednesday	CQ Transcriptions
House Armed Services Committee Holds Markup on Fiscal 2020 National Defense Authorization Act	June 12, 2019 Wednesday	CQ Transcriptions
House Armed Services Committee Holds Markup on Fiscal 2020 National Defense Authorization Act	June 12, 2019 Wednesday	CQ Transcriptions
House Armed Services Committee Holds Markup on Fiscal 2020 National Defense Authorization Act	June 12, 2019 Wednesday	CQ Transcriptions
House Foreign Affairs Committee Holds Hearing on Doha Agreement Enforcement	February 15, 2024 Thursday	CQ Transcriptions
House Financial Services Committee Holds Hearing on Practices of Private Funds	November 19, 2019 Tuesday	CQ Transcriptions
House Energy and Commerce Subcommittee on Energy Holds Hearing on Clean Energy Legislation	March 24, 2021 Wednesday	CQ Transcriptions
Hudson Institute Holds Webinar on Foreign Assistance and Religious Freedom	October 27, 2020 Tuesday	CQ Transcriptions
House Judiciary Subcommittee on Courts, Intellectual Property, and the Internet Hold Hearing on the Supreme Court	February 18, 2021 Thursday	CQ Transcriptions
Senate Health, Education, Labor and Pensions Committee Holds Hearing on Unions Improving Working Families Lives	November 14, 2023 Tuesday	CQ Transcriptions
Senate Environment and Public Works Committee Holds Hearing on Pending Nominations	March 11, 2020 Wednesday	CQ Transcriptions

House Appropriations Subcommittee On Transportation, Housing And Urban Development Holds Hearing on HUD's Housing Contracts Management During Shutdown	February 12, 2019 Tuesday	CQ Transcriptions
House Energy and Commerce Subcommittee on Energy Holds Hearing on FERC Oversight	June 12, 2019 Wednesday	CQ Transcriptions
House Energy and Commerce Subcommittee on Energy Holds Hearing on FERC Oversight	June 12, 2019 Wednesday	CQ Transcriptions
House Energy and Commerce Subcommittee on Energy Holds Hearing on FERC Oversight	June 12, 2019 Wednesday	CQ Transcriptions
House Judiciary Committee Holds Hearing on FBI Oversight	February 5, 2020 Wednesday	CQ Transcriptions
Senate Agriculture, Nutrition and Forestry Committee Holds Hearing on Farmers, Families and Rural Community Challenges and Opportunities	May 26, 2022 Thursday	CQ Transcriptions
House Natural Resources Committee Holds Hearing on US Park Police Response to Protests at DC's Lafayette Square	July 28, 2020 Tuesday	CQ Transcriptions
Senate Foreign Relations Committee Holds Hearing on Pending Nominations	March 12, 2019 Tuesday	CQ Transcriptions
White House Holds Regular News Briefing, as Released by the White House	June 22, 2021 Tuesday	CQ Transcriptions
House Judiciary Committee Holds Hearing on Impeachment: House Intelliegence Committee Counsel Presentations of Evidence	December 9, 2019 Monday	CQ Transcriptions
Senate Appropriations Subcommittee on Energy and Water Development Holds Hearing on Fiscal 2022 Budget Request for the Army Corps of Engineers and Bureau of Reclamation	June 9, 2021 Wednesday	CQ Transcriptions
White House Holds Regular News Briefing	June 22, 2021 Tuesday	CQ Transcriptions
House Foreign Affairs Committee Holds Hearing on State Department Foreign Policy Strategy and Fiscal 2022 Budget Request	June 7, 2021 Monday	CQ Transcriptions
Senate Budget Committee Holds Hearing on Budget Process Overhaul	June 19, 2019 Wednesday	CQ Transcriptions
Senate Budget Committee Holds Hearing on Budget Process Overhaul	June 19, 2019 Wednesday	CQ Transcriptions
Senate Budget Committee Holds Hearing on Budget Process Overhaul	June 19, 2019 Wednesday	CQ Transcriptions
House Homeland Security Committee Holds Hearing on Fiscal 2022 Budget Request for the Department of Homeland Security	June 17, 2021 Thursday	CQ Transcriptions
House Financial Services Committee Holds Hearing on Wells Fargo 's Consumer Issues	March 12, 2019 Tuesday	CQ Transcriptions
White House Coronavirus Task Force Members Hold News Conference, as Released by the White House	March 23, 2020 Monday	CQ Transcriptions
House Oversight and Reform Select Subcommittee on the Coronavirus Crisis Holds Hearing on Emergent Biosolutions	May 19, 2021 Wednesday	CQ Transcriptions

House Budget Committee Holds Hearing on Member Priorities	March 6, 2019 Wednesday	CQ Transcriptions
Senate Veterans' Affairs Committee Holds Hearing on VA Appropriations and Budget Request s	June 3, 2020 Wednesday	CQ Transcriptions
House Armed Services Subcommittee on Readiness Holds Hearing on Fiscal 2022 Budget Request for Military Readiness	June 9, 2021 Wednesday	CQ Transcriptions
House Natural Resources Committee Holds Hearing on the Fiscal 2021 Budget Request for the Interior Department	March 4, 2020 Wednesday	CQ Transcriptions
House Oversight and Reform Committee Holds Hearing	March 7, 2019 Thursday	CQ Transcriptions
House Armed Services Committee Holds Markup on Fiscal 2020 National Defense Authorization Act	June 12, 2019 Wednesday	CQ Transcriptions

Legislative Source

Legislative Source Title	Publication location	Publication	Publication	Notes
Congressional Record ID: CR-1984-0808	U.S. Federal	Congressional Record Retro (1873-1997)	LexisNexis Congressional Record Permanent Digital Collection	
Congressional Record ID: CR-1974-0123	U.S. Federal	Congressional Record Retro (1873-1997)	LexisNexis Congressional Record Permanent Digital Collection	
Congressional Record ID: CR-1975-0618	U.S. Federal	Congressional Record Retro (1873-1997)	LexisNexis Congressional Record Permanent Digital Collection	
168 Cong Rec H 1709	U.S. Federal	Congressional Record	CONGRESSIONAL RECORD -- HOUSE	117th Congress, 2nd SessionMarch 2022March 09, 2022HouseHouse of Representatives168 Cong Rec H 1709
168 Cong Rec H 1709	U.S. Federal	Congressional Record	CONGRESSIONAL RECORD -- HOUSE	117th Congress, 2nd SessionMarch 2022March 09, 2022HouseHouse of Representatives168 Cong Rec H 1709
168 Cong Rec H 1709	U.S. Federal	Congressional Record	CONGRESSIONAL RECORD -- HOUSE	117th Congress, 2nd SessionMarch 2022March 09, 2022HouseHouse of Representatives168 Cong Rec H 1709
Congressional Record ID: CR-1977-0314	U.S. Federal	Congressional Record Retro (1873-1997)	LexisNexis Congressional Record Permanent Digital Collection	
42 USCS § 4321	U.S. Federal	USCS - United States Code Service - Titles 1 through 54	United States Code Service	
Congressional Record ID: CR-1931-0209	U.S. Federal	Congressional Record Retro (1873-1997)	LexisNexis Congressional Record Permanent Digital Collection	
Congressional Record ID: CR-1897-0303	U.S. Federal	Congressional Record Retro (1873-1997)	LexisNexis Congressional Record Permanent Digital Collection	
CRS Report No. R47487	U.S. Federal	Congressional Research Service Reports	Congressional Research Service	

§ 15387. Urbanized Area	California	CA - Barclays Official California Code of Regulations	BARCLAYS OFFICIAL CALIFORNIA CODE OF REGULATIONS
§ 233.20 Need and amount of assistance.	U.S. Federal	CFR - Code of Federal Regulations	LEXISNEXIS' CODE OF FEDERAL REGULATIONS
§ 405.1885 Reopening a contractor determination or reviewing entity decision.	U.S. Federal	CFR - Code of Federal Regulations	LEXISNEXIS' CODE OF FEDERAL REGULATIONS

Supplementary References: News Media

News Source Title	Published date	Publication	Countries	Publication type
US ABS still facing supply constraints as disruptions continue	August 25, 2021 Wednesday	Global News + ICIS Chemical Business (ICB)	United Kingdom of Great Britain and Northern Ireland	Web Publication
AFRY's study supports Dutch Ministry of Economic Affairs and Climate Policy decision making for IJV Gamma and Nederwiek I offshore wind tender procedures	June 10, 2024 Monday	Contify Energy News	India, Republic of	Web Publication
US ABS still facing supply constraints as disruptions continue	August 25, 2021 Wednesday	Global News + ICIS Chemical Business (ICB)	United Kingdom of Great Britain and Northern Ireland	Newspaper
Boom highlights housing shortage	October 14, 2023 Saturday	The Chronicle (Toowoomba, Queensland)	Australia	Newspaper
Motor Grader Market Trends, Key Players, Development Status And Growth By 2031	December 1, 2023 Friday	MENAFN - Press Releases (English)	Middle East	Web Publication
Falling approvals show Vic is way off home building target Planning	February 28, 2024 Wednesday	Australian Financial Review	Australia	Newspaper
NEW LAW in California : Gavin Newsom approves measure for U.S. farmworkers in 2024	September 30, 2024 Monday	CE Noticias Financieras English	Mexico	Newspaper
Key Trends And Developments In The Single-Family Housing Construction Industry	September 18, 2023 Monday	MENAFN - Press Releases (English)	Middle East	Web Publication
Soaring construction costs dent home renos	September 1, 2022 Thursday	The Australian	Australia	Newspaper

Construction Equipment Market Analysis: Projecting Growth And Opportunities By 2032	July 4, 2023 Tuesday	MENAFN - Press Releases (English)	Middle East	Web Publication
New homes through floor	July 18, 2024 Thursday	Herald Sun/Sunday Herald Sun (Melbourne, Australia)	Australia	Newspaper
Handheld Laser Distance Meter Market Projected US$ 7.4B Revenue By 2033 Themarketpublicist	November 1, 2023 Wednesday	MENAFN - Market Reports (English)	Middle East	Web Publication
High Voltage Interlock Loop Connector Market To Grow Exponentially As A Result Of Rising Electric Vehicle Adoption And Renewable Energy Expansion | Research By SNS Insider	October 11, 2023 Wednesday	MENAFN - Press Releases (English)	Middle East	Web Publication
TREBOTTI: Leading Turnkey Industrial Construction	August 5, 2024 Monday	CE Noticias Financieras English	Mexico	Newspaper
Expanding Partnership with RenFuel	June 10, 2024 Monday	Market News Publishing	Canada	Newswire
Comstock Expanding Partnership With Renfuel	June 10, 2024 Monday	MENAFN - Press Releases (English)	Middle East	Web Publication
City & Country: Creating a win-win situation for businesses and environment	July 15, 2024	The Edge Malaysia	Malaysia	Newspaper
California approves regulations that will allow wastewater to be reused and blended directly with consumable water sources.	December 27, 2023 Wednesday	CE Noticias Financieras English	Mexico	Newspaper
Record house building activity to keep going	January 19, 2023 Thursday	The Australian	Australia	Newspaper
PPE Detection Market With A CAGR Of 78.9% Achieves Record-Breaking Growth In Meeting Healthcare Requirements By 2027	February 6, 2024 Tuesday	MENAFN - Press Releases (English)	Middle East	Web Publication
Swedbank Monthly newsletter - Macro Focus - Record EU funding enables transformation of the Baltic economies - Jun 22, 2021, The timing of EU funding increases overheating risks	June 22, 2021 Tuesday	Emerging Markets Brokers Reports - Central Eastern Europe	Europe	Newswire
New policy proposal dims hopes of reviving community solar in California	March 6, 2024 Wednesday	Energy Monitor Worldwide	Jordan, Hashemite Kingdom of	Web Publication
Swedbank Monthly newsletter - Macro Focus - Baltic Housing Affordability Index - 2021 Q4 - Mar 16, 2022	March 16, 2022 Wednesday	Emerging Markets Brokers Reports - Central Eastern Europe	Europe	Newswire

Rural homes should be prioritised in shift to heat pumps to tackle fuel poverty	August 3, 2024 Saturday	The Herald	Scotland	News paper
The Latest Construction Innovations And Industry Insights - September 4, 2024	September 4, 2024 Wednesday	MENAFN - Press Releases (English)	Middle East	Web Public ation
Global Polycarbonate Panels Market To Experience A Significant Revenue Cagr From 2023 To 2032 X Herald	September 5, 2023 Tuesday	MENAFN - Market Reports (English)	Middle East	Web Public ation
CMHC says 3.5M more homes needed for affordability	June 25, 2022 Saturday	The Daily Gleaner (New Brunswick)	New Brunswick	News paper
3.5M more homes needed: CMHC	June 25, 2022 Saturday	The Times & Transcript (New Brunswick)	New Brunswick	News paper
Legislation Risks Biden's Multi-Billion Dollar Investment in Hydrogen Future to Fight Climate Change	January 19, 2024 Friday	Gulf Oil & Gas	Egypt, Arab Republic of	Web Public ation
California Bill Would Study Ways To Slash Industrial Heat Process GHGs	May 8, 2024	EnergyWashington Week		Newsl etter
California Bill Would Study Ways To Slash Industrial Heat Process GHGs	May 5, 2024	California Energy & Climate Report		Newsl etter
California Bill Would Study Ways To Slash Industrial Heat Process GHGs	May 3, 2024	Inside EPA's Climate Extra		Newsl etter
Trade Ministry appoints Export Advisory Committees to overlook 24 sectors	October 15, 2021 Friday	The Daily Mirror (Sri Lanka)	Sri Lanka, Democratic Socialist Republic of	News paper
Canada needs 3.5 million more homes than projected to restore affordability, says CMHC	June 23, 2022 Thursday	National Post (f/k/a The Financial Post)(Canada)	Canada	News paper
Canada needs 3.5 million more homes than projected to restore affordability, says CMHC	June 23, 2022 Thursday	Postmedia Breaking News	Canada	News wire
Government of Saskatchewan rejects federal oil and gas emissions cap And Methane 75 regulations	September 26, 2024 Thursday	Maple Creek News	Canada	News paper
Labor, Business Groups: Legislation Risks Biden'S Multi-Billion Dollar Investment In California 'S Hydrogen Future To Fight Climate Change	January 20, 2024 Saturday	MENAFN - Business & Finance News (English)	Middle East	Web Public ation
Comstock Completes Renfuel Investment	April 24, 2024 Wednesday	MENAFN - Press Releases (English)	Middle East	Web Public ation
Building industry one area to dive	September 8, 2022 Thursday	Townsville Bulletin (Australia)	Australia	News paper

LOAN DEFAULTERS AFFECTING PRODUCTION	December 16, 2022 Friday	Times of Swaziland	Swaziland, Kingdom of	News paper
Renewable energy jobs rise	April 9, 2020 Thursday	The Age (Melbourne, Australia)	Australia	News paper
Anticipated Grease Cartridges Market Growth Exceeds 5% Themarketpublicist	October 27, 2023 Friday	Global English (Middle East and North Africa Financial Network)		News wire
EIB Group, ICO and BBVA Sign Agreements to Provide €1.26B	July 4, 2023 Tuesday	Gulf Oil & Gas	Egypt, Arab Republic of	Web Public ation
Completes RenFuel Investment	April 24, 2024 Wednesday	Market News Publishing	Canada	News wire
How To Choose The Right Material For Product Design	August 17, 2024 Saturday	MENAFN - Press Releases (English)	Middle East	Web Public ation
3D printing promises to transform architecture forever - and create forms that blow today's buildings out of the water Large-scale additive manufacturing, like desktop 3D printing, involves building objects one layer at a time. Whether it's clay, concrete or plastic, the print material is extruded in a fluid state and hardens into its final form	April 3, 2023 Monday	Indian Express	India, Republic of	News paper
Can California Tackle Zoning Run Amok in 2022?	April 12, 2022 Tuesday	ContentEngine Think Tank Newswire English		News paper
Exro Announces UL Certification For Cell Drivertm Energy Storage And Launch Of Cellex Energy Inc.	June 27, 2024 Thursday	MENAFN - Press Releases (English)	Middle East	Web Public ation
Housing, fuel price rises see inflation jump to 20-year high	April 28, 2022 Thursday	The Canberra Times	Australia	News paper
3D printing promises to transform architecture forever – and create forms that blow today's buildings out of the water	April 2, 2023 Sunday	Daily News Egypt	Egypt, Arab Republic of	News paper
EIB Group, ICO and BBVA sign agreements to provide (EURO)1.26 billion in new financing to support SMEs and boost energy efficiency	July 4, 2023 Tuesday	Contify Investment News	India, Republic of	Web Public ation
A carbon tax victory and Teck withdraws from Frontier	February 28, 2020 Friday	Sherwood Park News	Canada	News paper
Fuels Targets More Sustainable Aviation Fuel	December 19, 2023 Tuesday	Market News Publishing	Canada	News wire
California Bill To Create New Western Power Grid Operator Faces Defeat	May 3, 2023	EnergyWashington Week		Newsl etter
Global Construction Lifts Market Is Projected To Grow At A CAGR Of 7% By 2030 Report By Exactitude Consultancy	March 12, 2024 Tuesday	MENAFN - Press Releases (English)	Middle East	Web Public ation

Comstock Fuels Targets More Sustainable Aviation Fuel	December 19, 2023 Tuesday	MENAFN - Press Releases (English)	Middle East	Web Publication
NYSERDA: Governor Cuomo Announces 10 "Empire Building Challenge" Partnerships to Decarbonize High-rise Buildings, Combat Climate Change and Support the State's Green Economy	April 21, 2021 Wednesday	Contify Energy News	India, Republic of	Web Publication
California 's Offshore Wind Power Industry Sets Sail	May 5, 2023 Friday	Energy Monitor Worldwide	Jordan, Hashemite Kingdom of	Web Publication
Housing approvals fall to lowest level in 12 years despite Labor's pledge of 1.2m new homes	July 30, 2024 Tuesday	The Guardian (London)	United Kingdom of Great Britain and Northern Ireland	Newspaper
South Korea edges ahead of rivals to build Europe's nuclear reactors	July 21, 2024 Sunday	Business Mirror (Philippines)	Philippines, Republic of the	Newspaper
Crowding out our options to build on	September 10, 2023 Sunday	The Courier Mail/The Sunday Mail (Australia)	Australia	Newspaper
Comstock Executes First Biorefinery Commercial Agreement	December 29, 2023 Friday	Financial Services Monitor Worldwide (English)	Jordan, Hashemite Kingdom of	Web Publication
Pressure builds as housing costs soar	November 8, 2021 Monday	The Advertiser/Sunday Mail (Adelaide, South Australia)	Australia	Newspaper
New equipment loans back $863.6 million note issue from Kubota Credit Owner Trust	July 2, 2024	Asset Securitization Report		Newsletter
A carbon tax victory and teck withdraws from frontier	February 28, 2020 Friday	Sherwood Park News	Canada	Newspaper
Structural cracks in the housing policy Page Two	March 30, 2023 Thursday	Australian Financial Review	Australia	Newspaper
ABS Advances in Technology and Sustainability Discussed by UK Maritime Industry Leaders	June 21, 2022 Tuesday	Gulf Oil & Gas	Egypt, Arab Republic of	Web Publication
Home builders facing the perfect storm	June 18, 2022 Saturday	Western Advocate (ACM)	Australia	Newspaper
Excavator Market To Grow At A Cagr Of 3.2% By 2026 Construction Industry Is Expected To Hold The Largest Share X Herald	July 11, 2023 Tuesday	MENAFN - Market Reports (English)	Middle East	Web Publication
RWE buys 4.2 GW Norfolk offshore wind portfolio in UK	December 22, 2023 Friday	SeeNews Renewables	Denmark, Kingdom of	Newswire

Swedbank Monthly newsletter - Swedbank Macro Focus: Stability and Sustainability - Easing inflation, ebbing growth - Dec 21, 2023 - Latvia	December 21, 2023 Thursday	Emerging Markets Brokers Reports - Central Eastern Europe	Europe	News wire
California Governor Signs 18 Bills To Tackle Housing Affordability Crisis	October 10, 2019 Thursday	CE Noticias Financieras English	Mexico	News paper
Court ruling on development fees applauded by North Bay advocates pushing for local fee reform	April 16, 2024 Tuesday	The Press Democrat, Santa Rosa, Calif.		News paper
Executes First Biorefinery Commercial Agreement	December 28, 2023 Thursday	Market News Publishing	Canada	News wire
Machine Sensor Market Detailed Insights On Upcoming Trends 2023 - 2032	September 5, 2024 Thursday	MENAFN - Press Releases (English)	Middle East	Web Public ation
Comstock Executes First Biorefinery Commercial Agreement	December 28, 2023 Thursday	MENAFN - Press Releases (English)	Middle East	Web Public ation
New home approvals drop placing more pressure on supply	July 30, 2024 Tuesday	The Sydney Morning Herald (Australia) - Online	Australia	Web Public ation
Machine Sensor Market: Innovation And Product Optimization To Boost Growth	January 23, 2024 Tuesday	MENAFN - Press Releases (English)	Middle East	Web Public ation
Heavy Construction Equipment Market to Eyewitness Massive Growth by 2026 | AB Volvo , Rockland, Liebherr	September 21, 2021 Tuesday	MENAFN - Press Releases (English)	Middle East	Web Public ation
Infor agrees to sell its EAM Business to Hexagon AB and form strategic relationship Read Article Infor has entered into an agreement to sell its global EAM (Enterprise Asset Management) business to Hexagon AB (Nasdaq Stockholm: HEXA B), a global leader in digital reality solutions, for approximately $2.75 billion USD in a combination ...	July 8, 2021 Thursday	Indian Express	India, Republic of	News paper
DGAP-News: Nostromo To Work with Anheuser-Busch (AB) InBev to Advance Climate Action Goals Across its Facilities and Develop a Roadmap for Turning its Cooling Infrastructure Into Energy Storage Assets	September 14, 2021 Tuesday	EQS TodayIR	Hong Kong Special Administrative Region of China	Web Public ation
Machine Sensor Market Estimated To Experience A Hike In Growth By 2032	July 23, 2024 Tuesday	MENAFN - Press Releases (English)	Middle East	Web Public ation
Avenir LNG Expanding Marine Terminal Strategy to Decarbonize Swedish Port	August 19, 2022 Friday	Energy Monitor Worldwide	Jordan, Hashemite Kingdom of	Web Public ation
Heimstaden Bostad to receive SEK 3 billion EU financing for the construction of affordable and energy efficient housing	September 30, 2019 Monday	Cision Nordic Companies Press Releases (Scandinavia)	Sweden, Kingdom of	News wire

Heimstaden Bostad to receive SEK 3 billion EU financing for the construction of affordable and energy efficient housing	September 30, 2019 Monday	Cision Nordic Companies Press Releases (Scandinavia)	Sweden, Kingdom of	News wire
Australia's New Gas Strategy Makes for Flawed Foreign Policy	May 15, 2024 Wednesday	Energy Monitor Worldwide	Jordan, Hashemite Kingdom of	Web Public ation
Construction 4.0 Market To Hit Usd 54.5 Bn By 2032, Says Global Market Insights Inc.	August 8, 2023 Tuesday	MENAFN - Press Releases (English)	Middle East	Web Public ation
The Global Market for Cellulose Nanofibers to 2030 Featuring 87 In-depth Company Profiles (Products, Capacities, Production Processes, Target Markets & Commercial Activities)	December 19, 2019 Thursday	Global English (Middle East and North Africa Financial Network)		News wire
INSIGHT: Don't look to China for chemicals demand recovery in 2024	February 14, 2024 Wednesday	Global News + ICIS Chemical Business (ICB)	United Kingdom of Great Britain and Northern Ireland	Web Public ation
DIRTT Welcomes Empire Office To Its Global Partner Network	June 7, 2023 Wednesday	MENAFN - Press Releases (English)	Middle East	Web Public ation
Wisconsin veterans wouldn't have to pay college application fees under new bill	March 5, 2024 Tuesday	University Wire		News paper
European partnership seeks to bolster offshore solar deployment	February 14, 2024 Wednesday	SeeNews Renewables	Denmark, Kingdom of	News wire
Private capital investment	November 11, 2021 Thursday	Fort Saskatchewan Record	Canada	News paper
ABS Grants AIP for HHI 's Floating Storage & Offloading Design	February 11, 2021 Thursday	Gulf Oil & Gas	Egypt, Arab Republic of	Web Public ation
Cambrian Innovation Announces $18M of Dedicated Capital with Partners Spring Lane Capital	November 1, 2019 Friday	Financial Services Monitor Worldwide (English)	Jordan, Hashemite Kingdom of	Web Public ation
Grenada 's sun powers 14 breweries across Europe with a VPPA	October 24, 2022 Monday	CE Noticias Financieras English	Mexico	News paper
Renovate America opens up 2020 PACE pipeline	February 26, 2020	Global Capital Euroweek	United Kingdom of Great Britain and Northern Ireland	News paper
Spring Lane backs Cambrian Innovation	November 1, 2019 Friday	peHUB (Archive Only)		Web Public ation
Black & Veatch Management Consulting Launches New Cybersecurity Maturity Model Readiness Service	July 25, 2021 Sunday	MENAFN - Press Releases (English)	Middle East	Web Public ation

Black & Veatch Management Consulting Launches New Cybersecurity Maturity Model Readiness Service	July 20, 2021 Tuesday	MENAFN - Press Releases (English)	Middle East	Web Public ation
Korean shipbuilding giant makes waves in maritime SMR sector	February 8, 2024	Asia Power Monitor Today	England & Wales	News paper
Polystyvert secures $16M to revolutionize polystyrene recycling, enabling the global deployment of innovative technology for sustainable polystyrene production from plastic waste	July 9, 2024 Tuesday	RWE Resources News	Australia	News paper
Polystyvert secures $16M to revolutionize polystyrene recycling, enabling the global deployment of innovative technology for sustainable polystyrene production from plastic waste	July 9, 2024 Tuesday	RWE Resources News	Australia	News paper
U-turn on net metering: A blow to solarization	June 30, 2024 Sunday	Energy Update	Pakistan, Islamic Republic of	Maga zine
City wants Pure water project to flow again Council votes to remove pro-union terminology from construction contracts	November 6, 2019 Wednesday	The San Diego Union-Tribune		News paper
General Election 2024: What the major parties' manifestos have to say about tech	June 18, 2024 Tuesday	Computer Reseller News UK	United Kingdom of Great Britain and Northern Ireland	Maga zine
US Battery Player Advises Focus on Next-Gen Frontiers	January 10, 2023 Tuesday	Energy Intelligence News		Newsl etter
Northstar Announces First Draw Under $8.75 Million Project Loan Facility From BDC	September 12, 2024 Thursday	MENAFN - Press Releases (English)	Middle East	Web Public ation
Polystyvert Secures $16M to Revolutionize Polystyrene Recycling	July 8, 2024 Monday	Gulf Oil & Gas	Egypt, Arab Republic of	Web Public ation
The Many Unintended Consequences of AB 5	December 11, 2019 Wednesday	ContentEngine Think Tank Newswire English		News paper
Enerpure Inc. Advances Alberta Project By Securing Project Site	September 5, 2023 Tuesday	MENAFN - Press Releases (English)	Middle East	Web Public ation
No more rate rises, battling builders plead	April 4, 2023 Tuesday	The Australian	Australia	News paper
Korean shipbuilder joins maritime SMR project	February 7, 2024 Wednesday	World Nuclear News	England & Wales	News paper
Heliospectra (publ) Interim Report July - September 2021	October 22, 2021 Friday	Cision Nordic Companies Press Releases (Scandinavia)	Sweden, Kingdom of	News wire
Global Nanostructured Coatings, Films and Surfaces Market (2021 to 2031) - Key Drivers, Challenges and Opportunities	July 19, 2021 Monday	MENAFN - Press Releases (English)	Middle East	Web Public ation

Expect to see solar panels along San Diego highways per one of the energy bills Newsom has signed into law	October 10, 2023 Tuesday	The San Diego Union-Tribune		News paper
Expect to see solar panels along San Diego highways per one of the energy bills Newsom has signed into law	October 10, 2023 Tuesday	The San Diego Union-Tribune		News paper
Autodesk Continues to Outperform its Peers	September 9, 2024 Monday	Global Round Up - Sectors	Australia	News wire
Plasterboard Market Is Anticipated To Grow USD 37.34 Billion By 2030 At A CAGR Of 6.81% By Exactitude Consultancy	March 27, 2024 Wednesday	MENAFN - Press Releases (English)	Middle East	Web Public ation
Autodesk Continues to Outperform its Peers	September 3, 2024 Tuesday	Global Round Up - Sectors	Australia	News wire
Translation, interpreting and proofreading	September 16, 2021 Thursday	CE Noticias Financieras English	Mexico	News paper
Romania Food & Drink Profile: Ursus Breweries	September 13, 2024 Friday	Business Monitor News	England & Wales	Maga zine
Mayoral memo | Dubbo building approvals bucking the trend	September 20, 2023 Wednesday	(Dubbo) Daily Liberal	Australia	News paper
Mayoral memo | Dubbo building approvals bucking the trend	September 20, 2023 Wednesday	Mailbox Shopper	Australia	News paper
Autodesk Continues to Outperform its Peers	August 26, 2024 Monday	Global Round Up - Sectors	Australia	News wire
Autodesk Continues to Outperform its Peers	August 26, 2024 Monday	Global Round Up - Sectors	Australia	News wire
Sustainable Mining Solutions Industry Report 2023-2032: Global Market Sees Robust Growth, Driven by Technological Innovations and Favorable Policies	December 23, 2023 Saturday	Basic Materials & Resources Monitor Worldwide	Jordan, Hashemite Kingdom of	Maga zine
Autodesk Continues to Underperform its Peers	August 19, 2024 Monday	Global Round Up - Sectors	Australia	News wire
Autodesk Continues to Underperform its Peers	August 19, 2024 Monday	Global Round Up - Sectors	Australia	News wire
Point-to-point speed camera trial coming to NSW – as it happened	September 7, 2024 Saturday	The Guardian (London)	United Kingdom of Great Britain and Northern Ireland	News paper

Pay rises generate 1100 more jobs: economists	June 1, 2020 Monday	The Australian	Australia	News paper
AIMCo to sell interest in Spanish power producer Eolia	November 15, 2021 Monday	peHUB (Archive Only)		Web Public ation
Expands Global Presence with the Acquisition of Data Center Facility Located in Sweden	November 27, 2023 Monday	Market News Publishing	Canada	News wire
Alberta Recovery Plan	October 9, 2020 Friday	Sherwood Park News	Canada	News paper
Alberta Recovery Plan	October 9, 2020 Friday	Sherwood Park News	Canada	News paper
Blackstone Closes Sale of 42% Stake in Cheniere Energy Partners, L.P.	September 24, 2020 Thursday	Gulf Oil & Gas	Egypt, Arab Republic of	Web Public ation
Gwynedd Council to deliver 1,500 affordable homes	December 8, 2020 Tuesday	North Wales Chronicle	England & Wales	News paper
Builders' burdens locked in for years	May 26, 2022 Thursday	The Australian	Australia	News paper
Lack of housing for workers could hold region back, think tank says	January 23, 2024 Tuesday	Newcastle Herald	Australia	News paper
Keyless Entry System Market is Expected to Surpass Revenues Worth US$ 14.02 Billion by 2033, Persistence Market Research	April 25, 2023 Tuesday	Financial Services Monitor Worldwide (English)	Jordan, Hashemite Kingdom of	Web Public ation
China round-up: Beijing rolls out more stimulus policies	May 27, 2022 Friday	Global Capital Euroweek	United Kingdom of Great Britain and Northern Ireland	News paper
Ancient Swedish Hamlet Holds Lessons for Future of Clean Power	May 15, 2021 Saturday	National Post (f/k/a The Financial Post)(Canada)	Canada	News paper
Ancient Swedish Hamlet Holds Lessons for Future of Clean Power	May 15, 2021 Saturday	Postmedia Breaking News	Canada	News wire
parties' key Solutions for housing	April 9, 2024 Tuesday	The Chronicle (Toowoomba, Queensland)	Australia	News paper
Takeover of telcos possible, but costly for virus-hit PH	July 30, 2020 Thursday	Philippines Daily Inquirer	Philippines, Republic of the	News paper
Mitsubishi Fuso launches the first light-duty Euro V truck in the UAE	December 23, 2020 Wednesday	Contify Automotive News	India, Republic of	Web Public ation

U-turn on net metering	May 20, 2024 Monday	Business Recorder	Pakistan, Islamic Republic of	News paper
Regulatory Approval of Marwayne Pilot Project	October 16, 2020 Friday	Market News Publishing	Canada	News wire
Lockdown drives jobless rate up	October 16, 2020 Friday	The Australian	Australia	News paper
Epiroc interim report Q2 2021	July 20, 2021 Tuesday	Cision Nordic Companies Press Releases (Scandinavia)	Sweden, Kingdom of	News wire
Metalworking Tool Holder Market Is Projected To Witness A Rise In Revenue Of Us$ 1,216.2 Million By 2031	July 10, 2023 Monday	MENAFN - Press Releases (English)	Middle East	Web Public ation
Construction industry banded together to survive COVID-19 | B2B Central	December 4, 2020	South African Food Review	South Africa, Republic of	Maga zine
How the BIBC helped small construction businesses stay afloat, survive Covid-19	November 25, 2020 Wednesday	Bizcommunity.com (South Africa: Other industries)	South Africa, Republic of	Newsl etter
N.J. Amps Up Wind Fight, Overriding Beach Towns Balking at Farms	July 22, 2021 Thursday	Postmedia Breaking News	Canada	News wire
Global Thermal Camera Market to Generate Revenue of $3.82 Billion by 2028 | China, US and India are Top 3 Manufacturers and Exporters of Thermal Camera	September 30, 2022 Friday	Financial Services Monitor Worldwide (English)	Jordan, Hashemite Kingdom of	Web Public ation
Miners' Strategy 2020: Resilience Built Since 2015 To Be Tested By Covid-19	April 23, 2020 Thursday	Business Monitor News	England & Wales	Maga zine
Why Nigeria must promote additive manufacturing technology - RMRDC DG	January 18, 2020 Saturday	Weekly Trust	Nigeria, Federal Republic of	News paper
Geothermal, Oil Drilling Bond Strengthens	March 9, 2023 Thursday	Energy Monitor Worldwide	Jordan, Hashemite Kingdom of	Web Public ation
Monetary, fiscal policy synergy as elixir for macro-economic balance	October 26, 2022 Wednesday	The Sun (Nigeria)	Nigeria, Federal Republic of	News paper
Inpixon to Provide Its CO2 Sensors to Help Identify COVID-19 Infection Risk	February 11, 2021 Thursday	MENAFN - Press Releases (English)	Middle East	Web Public ation
Floating Offshore Wind Buoyant on New Developments, Projects	December 2, 2019 Monday	Energy Monitor Worldwide	Jordan, Hashemite Kingdom of	Web Public ation
Alberta Grievance Arbitration Awards decision: Construction Workers Union, CLAC Local 63 v Brock Canada Field Services Ltd., 2022 CanLII 22228 (AB GAA)	March 31, 2022 Thursday	Baltic Legal Updates	Europe	News wire

Volt-VAR control for distribution networks with high penetration of DGs: An overview	June 2022	The Electricity Journal		Journal
SBAB Bank AB (publ): SBAB Interim Report January-June 2021	July 16, 2021 Friday	EQS TodayIR	Hong Kong Special Administrative Region of China	Web Publication
Petroperu : sustainability of state-owned company at risk due to political interference	May 15, 2022 Sunday	CE Noticias Financieras English	Mexico	Newspaper
ABS reveals $5B increase to renewable investment	October 12, 2022 Wednesday	Energy Monitor Worldwide	Jordan, Hashemite Kingdom of	Web Publication
US M&A Deals: RR Donnelley , Quest Softwar, Fertitta Entertainment	December 6, 2021 Monday	SeeNews Deals	Europe	Wire
Argentina Automotive 18 Mar 24	March 18, 2024	Industry SnapShot		Newsletter
Argentina Automotive 20 Nov 23	November 20, 2023	Industry SnapShot		Newsletter
Argentina Automotive 30 Oct 23	October 30, 2023	Industry SnapShot		Newsletter
India : Year End Review of Ministry of Power (up to November 2023)	January 5, 2024 Friday	Thai News Service	Thailand, Kingdom of	News wire
EY Entrepreneur Of The Year: 2019 Philippines From homeless to home builder	October 3, 2019 Thursday	BusinessWorld	Philippines, Republic of the	Newspaper
Federal Court of Australia Judgment: Hanwha Solutions Corporation v REC Solar Pte Ltd [2023] FCA 1017	September 23, 2023 Saturday	Baltic Legal Updates	Europe	News wire
LATIN AMERICA - ARGENTINA - AUTOMOTIVE - UPDATED 19 JULY 2021	July 19, 2021	Industry SnapShot		Newsletter
Argentina Automotive 1 Apr 24	April 1, 2024	Industry SnapShot		Newsletter
Acceleware Ltd. Reports First Quarter 2021 Financial and	May 27, 2021 Thursday	MENAFN - Press Releases (English)	Middle East	Web Publication
First Quarter 2021 Financial and Operating Results	May 27, 2021 Thursday	Market News Publishing	Canada	News wire
Acceleware Ltd. Reports First Quarter 2021 Financial and Operating Results	May 27, 2021 Thursday	Postmedia Breaking News	Canada	News wire
Acceleware Ltd. Reports First Quarter 2021 Financial and Operating Results	May 27, 2021 Thursday	National Post (f/k/a The Financial Post)(Canada)	Canada	Newspaper
PFR Solar ABS Roundtable 2020/21	January 29, 2021	Power, Finance and Risk		Newsletter

India faces uphill struggle as bridge-builder at G20	September 8, 2023 Friday	FT.com Headlines	England & Wales	Web Publication
Acceleware Ltd. Reports Second Quarter 2021 Financial and Operating Results	August 25, 2021 Wednesday	MENAFN - Press Releases (English)	Middle East	Web Publication
Acceleware Ltd. Reports Second Quarter 2021 Financial and Operating Results	August 25, 2021 Wednesday	Postmedia Breaking News	Canada	Newswire
Acceleware Ltd. Reports Second Quarter 2021 Financial and Operating Results	August 25, 2021 Wednesday	National Post (f/k/a The Financial Post)(Canada)	Canada	Newspaper
Supreme Court of Ireland Decision: Balz & anor v An Bord Pleanala & anor (Unapproved) [2020] IESC 22 (05 May 2020)	May 7, 2020 Thursday	Baltic Legal Updates	Europe	Newswire
England and Wales High Court (Technology and Construction Court) Decision: Munkenbeck and Marshall & Anor v The Vinyl Factory Ltd & Ors [2019] EWHC 3225 (TCC) (02 December 2019)	December 5, 2019 Thursday	Baltic Legal Updates	Europe	Newswire
Smart Energy	May 28, 2021 Friday	Mail & Guardian	South Africa, Republic of	Newspaper
Bertrand de Mazi&res, EIB: 'all of us have to do more in times of crisis'	July 9, 2020	Global Capital Euroweek	United Kingdom of Great Britain and Northern Ireland	Newspaper
Earns $3.7 Million, or $0.40 Per Diluted Share, in Third Quarter 2020; Net Interest Margin Expansion and Improved Efficiency Ratio Generate Record Earnings; Announces Share Repurchase Plan for 10%	October 28, 2020 Wednesday	Market News Publishing	Canada	Newswire
France Banking 9 July 2024_EN	July 9, 2024	Industry SnapShot		Newsletter
France Banking 4 Jun 24	June 4, 2024	Industry SnapShot		Newsletter
